# Dibenzoacridinium derivatives: a new class of G-quadruplex ligands with anti-HIV-1 properties

**DOI:** 10.1039/d5md01178g

**Published:** 2026-07-06

**Authors:** Amani Kabbara, Corinne Buré, Aurore Guédin, Brice Kauffmann, Eric Largy, Julien Marquevielle, Pierre Bonnafous, Zidane Mdarhri, Yann Ferrand, Valérie Gabelica, Frédéric Rosu, Marie-Line Andreola, Céline Olivier, Samir Amrane

**Affiliations:** a ARNA Laboratory, Inserm U1212, CNRS UMR 5320, Université de Bordeaux France samir.amrane@u-bordeaux.fr; b Institut Européen de Chimie et Biologie, Univ. Bordeaux, CNRS, Inserm, IECB US1, UAR 3033 F-33600 Pessac France; c Laboratoire de Chimie et Biologie des Membranes et des Nano-objets, UMR 5248 CNRS, Université de Bordeaux 2 rue Robert Escarpit F-33600 Pessac France; d Univ. Bordeaux, CNRS, Microbiologie Fondamentale et Pathogénicité, UMR 5234 F-33000 Bordeaux France; e Institut des Sciences Moléculaires, UMR 5255 CNRS, Université de Bordeaux 351 Cours de la Libération F-33405 Talence Cedex France celine.olivier@u-bordeaux.fr

## Abstract

G-quadruplexes (G4s) are non-canonical nucleic acid structures formed by guanine-rich sequences that assemble into stacked guanine tetrads. These unusual RNA or DNA structures are implicated in essential regulatory processes in both human and viral genomes. In recent years, compounds that bind and stabilise G4s have emerged as promising antiviral agents against several viruses such as HIV-1, HCV or HPV. We report the design, synthesis, and biophysical characterization of a novel class of G4-stabilizing ligands based on a positively charged dibenzoacridinium (DBA) core. A combination of FRET-melting assays, fluorescence quenching assay (FQA), circular dichroism spectroscopy, native mass spectrometry, NMR titration, and molecular dynamics simulations confirmed that DBA ligands bind selectively to G4s, with thermal stabilization (Δ*T*_m_) values reaching +18 °C and dissociation constants (*K*_d_) as low as 0.14 μM. In cellular assays, DBA4 and DBA5 compounds exhibited antiviral activity against HIV-1, with IC_50_ values of 1 μM and 3 μM, respectively. These results identify DBAs as a new class of G-quadruplex ligands with anti-HIV properties.

## Abstract 2

HIVpro1 sequence is derived from HIV-1 promoter. It forms a non-canonical DNA structure called G-quadruplex (or G4) that is assembled trough the stacking of two guanine tetrads. DBA1 ligand binds to the external tetrad of HIVpro1 G4.

## Graphical abstract

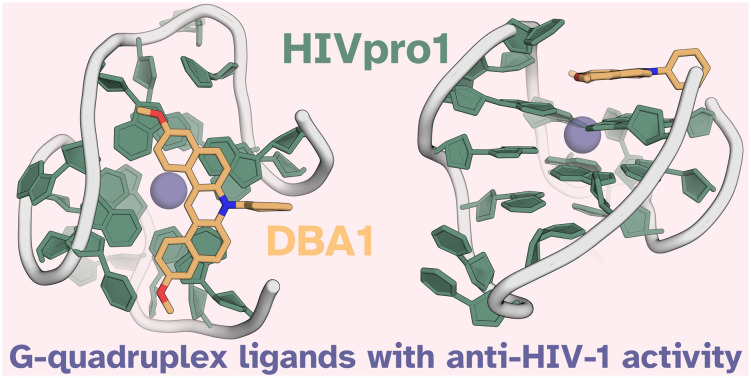

## 1. Introduction

### 1.1 HIV-1: the need for new antiviral strategies

HIV-1 RNA retrovirus infects CD4 memory T cells. This virus is responsible for a deficiency in the immune system that leads to AIDS disease. Over the past 40 years, HIV-1 has become a serious global health issue. As of 2024, approximately 40.8 million people were living with HIV-1, including about 1.4 million children aged 0–14 years. In the same year, around 1.3 million people became newly infected with HIV-1, highlighting ongoing transmission despite global prevention efforts. The development of antiretroviral therapy (ART) since the 1980s has transformed HIV-1 from a fatal diagnosis into a manageable chronic condition. Life expectancy for people living with HIV-1 who achieve viral suppression now approaches that of the general population.^1^ These successes have dramatically reduced AIDS-related mortality worldwide and represent one of the greatest achievements in modern medicine. Despite these advances, significant challenges remain that underscore the continued need for novel therapeutic strategies. First, ART requires lifelong adherence, as treatment interruption leads to rapid viral rebound from latent reservoirs.^2,3^ Second, the emergence of drug-resistant strains threatens long-term treatment success.^4^ Recent surveillance data reveal alarming rates of acquired resistance to non-nucleoside reverse transcriptase inhibitors (NNRTIs) and integrase strand transfer inhibitors (INSTIs), particularly in resource-limited settings where access to resistance testing is constrained.^5^ Third, long-term ART use is associated with various toxicities and comorbidities. Nucleoside reverse transcriptase inhibitors (NRTIs) can cause mitochondrial dysfunction leading to lactic acidosis and peripheral neuropathy^6^ and INSTIs, while generally well-tolerated, have been associated with weight gain and neuropsychiatric effects in some patients.^7^ The search for alternative antiviral strategies that could complement or enhance existing therapies therefore remains crucial from both clinical and public health perspectives. Novel approaches targeting different aspects of the viral lifecycle, including viral nucleic acid structures, may offer opportunities to overcome current limitations and move toward functional cure strategies.

### 1.2 G4 DNA and RNA in viruses

**Fig. 1:**
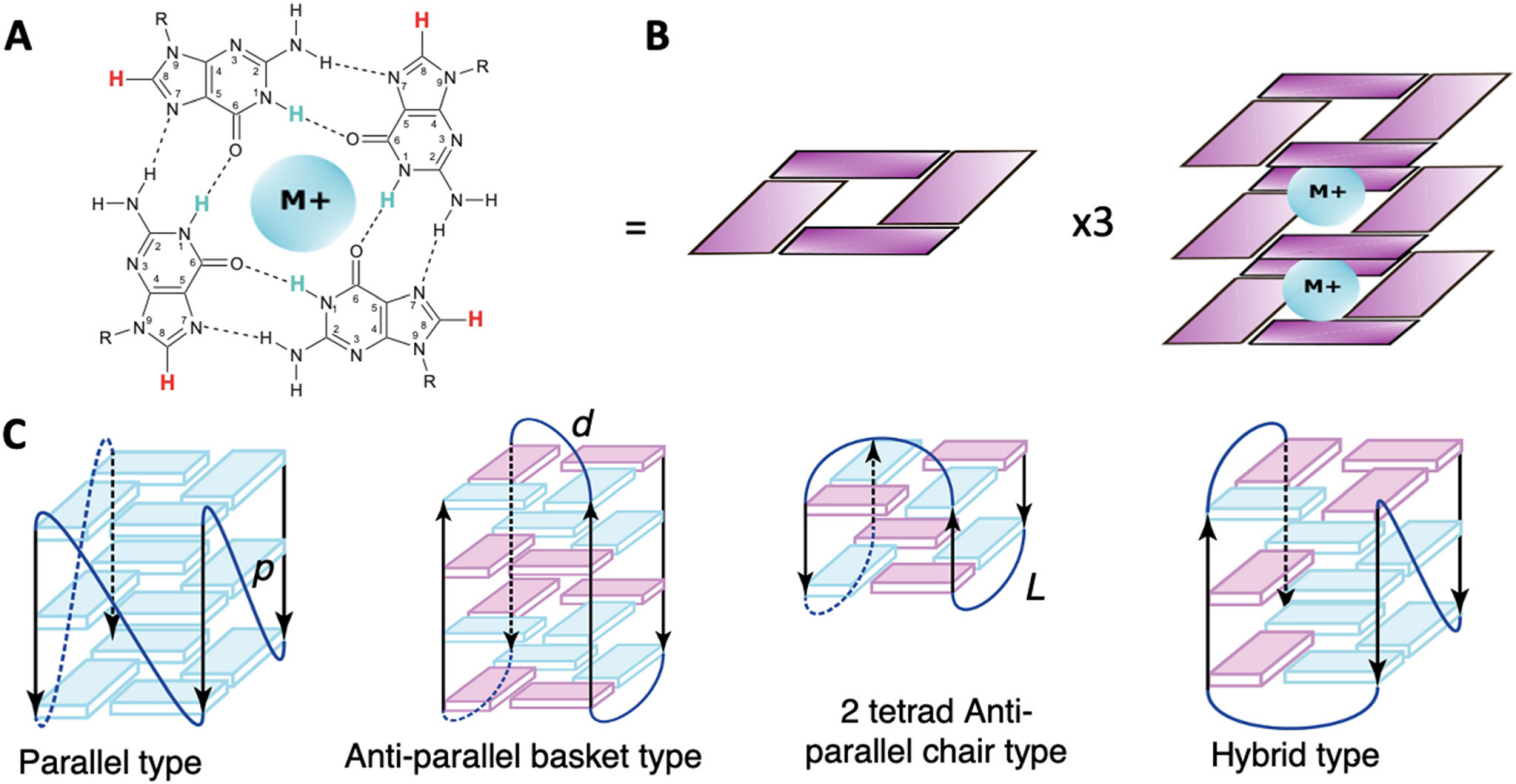
A. G-quartet structure stabilized by a monovalent cation (M^+^ = Na^+^ or K^+^). B. Several quartets (here three) can self-assemble and sandwich cations. C. Schematic representation of various topologies of intramolecular G-quadruplex structures composed of two, three or four tetrads with three different loop types: propeller (p), diagonal (d), and lateral (l). Guanines can adopt either *syn* (purple) or *anti* (cyan) glycosidic bond angles.

G-quadruplexes (or “G4”) are non-canonical nucleic acid structures formed by guanine-rich DNA or RNA sequences. They consist of four strands and are stabilized by the formation of G-tetrads^8^ (Fig. 1): square planar arrangements of four guanines connected by eight hydrogen bonds. Potassium or sodium cations, prevalent in the cellular environment, stabilize G4s through specific electrostatic interactions with the carbonyl groups of the guanines.^9,10^ The stacking of two or more G-tetrads forms the core of the G4 structure, which is composed of guanine columns at each corner.^11^ A growing body of studies demonstrates the involvement of these unique conformations in key biological processes.^12^ Genome-wide bioinformatics studies have revealed the distribution of putative G4-forming sequences (PQS) in the human genome. Depending on the algorithm used, up to 1.5 million PQS can be detected, with approximately two-thirds of human promoters containing at least one PQS and 37% of predicted recombination sites also harbouring a PQS.^13–15^ These correlations strongly suggest biological functions for these G4 structures. Furthermore, *in vivo* studies utilizing fluorescent G4-probes, such as antibodies or ligands, have confirmed the presence of G4s in genomic DNA and cellular RNA.^16–18^ More recently, a new approach called G4access has highlighted the association of G4 formation with open chromatin and transcription.^19^ Gene transcription can also be regulated by the use of small-molecule G4 binders that target G4s located in promoters.^20^

G4s are also present in some microbial pathogens including bacteria^21^ and viruses.^22^ Despite the outstanding ability of viruses to evolve extremely rapidly, which allows the virus to escape the immune response of the infected organism, G4s are conserved in some viruses indicating a biological function. For instance, in hepatitis C virus, it has been proposed that negative RNA synthesis is modulated by G4 formation.^23,24^ RNA G4s were also identified within untranslated regions (UTRs) and open reading frames (ORFs) of the viral RNA of SARS-CoV-2 responsible for Covid-19.^25^

In the particular case of HIV-1, several studies have shown the presence of evolutionarily conserved G4s in the viral genome. Notably, some of these G4s are contained in crucial regulatory elements such as the PPT and cPPT sequences as well as the U3 region.^26^ It has been suggested that recombination and/or genomic dimerization can be achieved *via* the formation of a bimolecular G4 using the cPPT, PPT, and/or U3 sequences of each viral RNA.^27–29^ The same mechanism has also been proposed for a G-rich sequence located at the 5′ end of the gag gene. The NCp7 protein, known to facilitate viral RNA packaging, may play a role in this dimerization.^30^ At the DNA level, the transcription of the HIV-1 viral DNA is regulated by a G-rich region of the viral promoter located in the U3 region. This segment contains the three binding sites of the SP1 transcription factor.^31,32^ Several studies have shown that this particular region can form four types of G4s that may be involved in transcription regulation:^32,33^ i) HIVpro1 forms a two tetrad antiparallel G4,^31^ ii) HIVpro2 forms a three tetrad hybrid G4,^32^ iii) LTR-IV forms a parallel G4^34^ and iv) LTR-III also forms a three tetrad hybrid G4.^35^ As a result, HIV-1 presents several DNA and RNA G4s associated with important functions.

### 1.3 G4s as antiviral targets

In recent years, the unique structural properties of G4s have made them an attractive therapeutic target. Unlike other nucleic acid structures, G4s are fairly compact and polymorphic which suggests that a high degree of selectivity can be achieved when targeting them. Consequently, many laboratories are working on discovering molecules that specifically bind to G4s, offering an alternative to typical protein-targeting strategies.^36,37^ Recent studies have reported the antiviral effects of various G4 ligands from different chemical families, such as naphthalene diimides,^38–40^ acridine,^41^ porphyrins^26,42^ or metallo-supramolecular complexes.^43^ These ligands have been shown to inhibit DNA viruses like herpesviruses (HSV, EBV), monkeypox virus and hepatitis B virus, as well as RNA viruses such as hepatitis C virus or HIV-1 (reviewed here^44^). In the case of HIV-1, targeting and stabilizing viral RNA G4s from the PPT, cPPT and U3 region with porphyrin derivatives can block the initiation of reverse transcription within the first two hours of the HIV-1 viral cycle.^26^ Naphthalene diimides^38^ and acridine derivatives^41^ have also been proposed to inhibit HIV-1 by targeting the viral G4 located in the promoter region. These inhibition mechanisms represent promising new antiviral strategies that target viral RNAs and DNAs. In this context, we present here the synthesis of novel dibenzoacridinium derivatives (DBA) as new G4 ligands. We characterized their binding properties to several DNA and RNA conformations and showed promising antiviral activities against HIV-1.

## 2. Materials and methods

### 2.1 Synthesis of the compounds

All reagents were obtained from commercially available sources and used without further purification. Solvents were dried from appropriate drying agents (sodium for toluene; calcium hydride for dichloromethane) and freshly distilled before use. Dimethylformamide was purified through azeotropic distillation with water and benzene.

^1^H NMR and ^13^C NMR analyses were performed on Bruker Advance 300 and DPX 400 spectrometers. Chemical shift values are given in ppm with reference to solvent residual signals (Fig. S12–S21). HR-MS analyses were performed on a Qstar spectrometer. **All compounds are >95% pure by NMR analysis**.

Synthetic routes (detailed protocols for each compound are provided in SI) follow two general procedures:

**General procedure A** (Buchwald–Hartwig cross-coupling reaction). In dry and degassed toluene were introduced Pd(OAc)_2_ (0.06 equiv.) and P(*t*-Bu)_3_ (0.12 equiv.). After 15 min of stirring, aniline derivative (1 equiv.), bromonaphthalene derivative (4 equiv.) and Cs_2_CO_3_ (3 equiv.) were added successively. The solution was refluxed for three days, cooled down to RT and diluted with CH_2_Cl_2_. The crude mixture was filtered, evaporated to dryness and purified on a silica gel column (cyclohexane/CH_2_Cl_2_ 8 : 2, v/v) to afford the target compounds as coloured powders.

**General procedure B** (synthesis of dibenzoacridinium compounds). In a general procedure, the arylamine precursor (1 eq.) was solubilized in dry DMF and the mixture was cooled down to 0 °C. Phosphorus oxychloride (2 eq.) was added dropwise under continuous stirring. The reaction mixture was allowed to warm up to room temperature and further heated up to 90 °C and stirred for 3 h. After removal of the solvent under vacuum, the crude product was dissolved in a mixture of dichloromethane and methanol (8 : 1). The target compound precipitated as a highly coloured solid by addition of ethyl acetate. The crude product was purified on a silica gel column (CH_2_Cl_2_/MeOH (9 : 1, v/v)) to afford dibenzoacridinium compounds as deeply coloured powders.

### 2.2 Biophysical characterisation

#### 2.2.1 FRET melting assay

Doubly labelled oligonucleotides with fluorescein and TAMRA were purchased lyophilized and RP-cartridge purified from Eurogentec (Kanaka Eurogentec, Seraing, Belgium). FRET melting experiments were run on Stratagene Mx3005P real-time PCR equipment in 96-well plates on the DNA sequences listed Table 1. Experiments were performed in 10 mM lithium cacodylate buffer (pH 7.2) and 10 mM KCl and 90 mM LiCl as previously described.^45,46^ The DNA concentration was 0.2 μM. The stabilization (Δ*T*_1/2_) induced by compounds was calculated as the difference between the *T*_m_ of the nucleic acid alone and that measured with a ligand concentration of 1 μM. Data are presented as averages of three independent measurements, each conducted in duplicate (*λ*_exc_ = 492 nm, *λ*_em_ = 516 nm, *T* interval = 25–95 °C, ramp: 25 °C for 5 min, then 1 °C min^−1^, measurements every 1 °C, 8× magnification of the fluorescence signal). The G4 selectivity of the ligands with respect to the duplex was evaluated by determining the *T*_m_ values in the presence of 0, 3 or 10 μM of the duplex competitor ds26.

**Table 1:** Oligonucleotides

	Name	Sequence 5′ → 3′	Conformation	DNA/RNA	Fluorescent labels
1	LTR4^34^	GGGCGGGACTGGGGAGTGGC	Parallel G4	DNA	
	LTR4-FT	F-TGGGCGGGACTGGGGAGTGGC-T	Parallel G4	DNA	5′Fluorescein/3′TAMRA
2	HIVpro1^31^	TGGCCTGGGCGGGACTGGG	Anti-parallel G4	DNA	
	HIVpro1-FT	F-TGGCCTGGGCGGGACTGGG-T	Anti-parallel G4	DNA	5′Fluorescein/3′TAMRA
3	HIVpro2^32^	AGGGAGGCGTGGCCTGGGCGGG	Hybrid G4	DNA	
	HIVpro2-FT	F-AGGGAGGCGTGGCCTGGGCGGG-T	Hybrid G4	DNA	5′fluorescein/3′TAMRA
4	rLTR4^26^	GGGCGGGACUGGGGAGUGGC	Parallel G4	RNA	
	rLTR4-3′Cy5	GGGCGGGACUGGGGAGUGGC-Cy5	Parallel G4	RNA	3′Cy5
	rLTR4-5′Cy5	Cy5-UGGGCGGGACUGGGGAGUGGC	Parallel G4	RNA	5′Cy5
5	Htel	AGGGTTAGGGTTAGGGTTAGGG	Mixture of G4s	DNA	
	Htel	F-AGGGTTAGGGTTAGGGTTAGGG-T	Mixture of G4s	DNA	5′Fluorescein/3′TAMRA
6	TG4T	TGGGGT	Parallel G4	DNA	
7	G4T4G4	GGGGTTTTGGGG	Anti-parallel G4	DNA	
8	Ds26	CAATCGGATCGAATTGATCCGATTG	Duplex	DNA	
9	DK66	CGCGAATTCGCG	Duplex	DNA	
10	DK100	CGCGGGCCCGCG	Duplex	DNA	

#### 2.2.2 Ligand-induced fluorescence quenching assay

Cy5 labelled oligonucleotides were purchased lyophilized and RP-cartridge purified from Eurogentec (Kanaka Eurogentec, Seraing, Belgium). The ligand-induced fluorescence quenching assay was conducted as previously described.^47^ A Stratagene Mx3005P instrument was used to carry out the assay in 96-well plates. The filters for excitation and emission were set at 635 nm and 665 nm, respectively. The gain was used at 8×. The labeled oligonucleotide was used at 10 nM, and the buffer was 10 mM lithium cacodylate (pH 7.2), 10 mM KCl, and 90 mM LiCl in a final volume of 50 μL. The oligonucleotide was heated at 90 °C for 5 min and put in ice to anneal. The ligand was added over a range of concentrations between 0 and 80 μM. A competitor, ds26, was also added at 3 or 10 μM. Each experimental condition was tested in triplicate except for the ligand alone with the buffer, which was tested in duplicate. The instrument was programmed to heat the plate for 10 min at 25 °C and measure the fluorescence after 1 min for 5 cycles. The fluorescence of each sample was normalized, and the fluorescence of the ligand alone was subtracted.

#### 2.2.3 Circular dichroism spectroscopy

Oligonucleotides were purchased lyophilized and RP-cartridge purified from Eurogentec (Kanaka Eurogentec, Seraing, Belgium). CD experiments were performed with a JASCO J-1500 spectropolarimeter (JASCO, Lisses, France) using quartz cells of 0.2 cm path length. The scans were recorded at 22 °C, from 220 to 370 nm with the following parameters: 1.0 nm data pitch, 2 nm bandwidth, 0.5 s response, 50 nm min^−1^ scanning speed; they are the result of three accumulations.

#### 2.2.4 Mass spectrometry

Oligonucleotides were purchased lyophilized and RP-cartridge purified from Eurogentec (Kanaka Eurogentec, Seraing, Belgium), then buffer exchanged with ammonium acetate (5 M; Sigma-Aldrich, Saint-Quentin-Fallavier, France) using Amicon Ultra (3 K cut-off; Merck Millipore, Cork, Ireland). The concentrations of the initial stock solutions of DNA/RNA (∼1 mM) were measured by UV absorbance at 260 nm. Sample solutions were prepared by diluting the appropriate volume of oligonucleotide stock solutions to reach 10 μM of DNA/RNA and 20 μM of ligand, in 100 mM ammonium acetate. The solutions were incubated for 16 h at 4 °C before analysis by mass spectrometry. The affinity and stoichiometry of binding to oligonucleotides were determined by native electrospray ionization mass spectrometry in the negative ion mode on a Thermo Orbitrap Exactive mass spectrometer calibrated daily, and operated in negative mode, in a 400–3000 *m*/*z* scan range, using the 50 000 resolution setting, with the following tuning parameters: spray voltage: 3.2 kV, capillary temperature: 170 °C, sheath gas: 60, aux gas: 0, heater temperature: 35 °C, tube lens voltage: −175 V, capillary voltage: −17 V, skimmer voltage: −22 V. The syringe injection flow rate was 3 μL min^−1^. These parameters ensured a good signal intensity without disrupting noncovalent complexes, which was verified using d[G4T4G4]_2_ as a control. The presented spectra of all DNA/RNA used in this work (Fig. 5, S9 and S10) result from 8 min accumulations. The abundance of each species, M, M + 1L, M + 2L and M + 3L complexes, where M is a monomer oligonucleotide and L is a ligand, was determined from their peak area signals (*A*), from the sum of the −4, −5 and −6 charge states for DNA and from the sum of the −5, −6 and −7 charge states for RNA, assuming that their response factors are equal. The molar concentrations were then calculated knowing the total amount of oligonucleotide [M]_tot_:
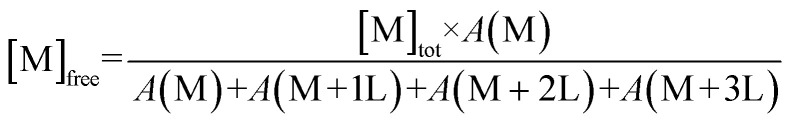

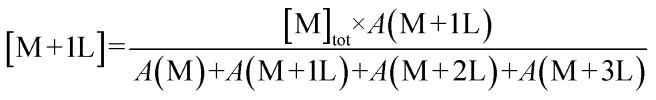

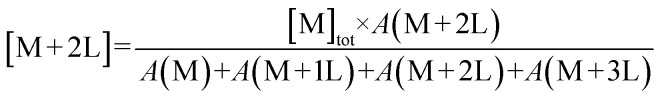

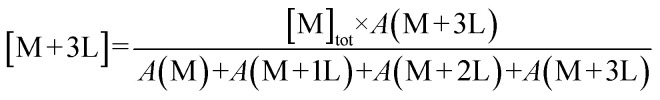
The concentration of the free ligand, [L]_free_, was determined from its total concentration [L]_tot_:[L]_free_ = [L]_tot_ − [M + 1L] − 2[M + 2L] − 3[M + 3L]Apparent consecutive dissociation constants (*K*_d1_, *K*_d2_ and *K*_d3_) were calculated by using the following equations:
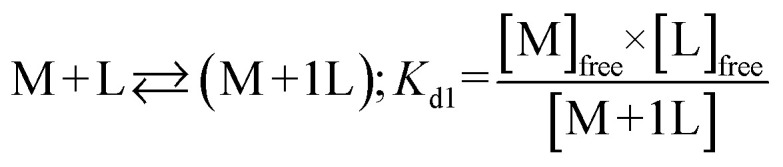

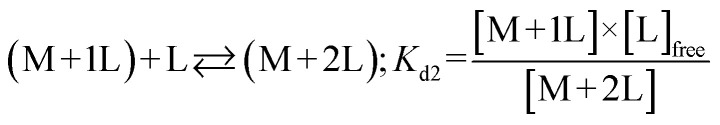

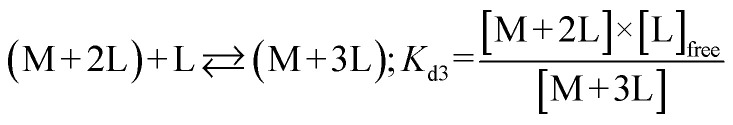


#### 2.2.5 X-ray crystallography

Diffraction data for compound DBA1 were measured on a Bruker microstar X8 PROTEUM rotating anode at the copper kα wavelength. The structure was solved with the dual-space algorithm implemented in SHELXT and refined by the full-matrix least-squares method on *F*^2^ with SHELXL-2014^48^ within Olex2.^49^

#### 2.2.6 NMR experiment

A 700 MHz Bruker spectrometer was used to perform the NMR experiments. Sample strand concentration was around 150 μM dissolved in 20 mM potassium phosphate pH 7 and 70 mM KCl in 90% H_2_O/10% D_2_O in 5 mm tubes. The 1D-^1^H-NMR spectra were recorded using a double pulse field gradient perfect spin echo (zgesgppe) pulse sequence to suppress the water signal. Spectra were recorded with a spectral width of 19 ppm, an acquisition time of 1.2 s and a relaxation delay of 2 s. Experiments were performed at 25 °C.

#### 2.2.7 Docking and molecular dynamics

HIVpro1 was prepared for docking by flipping out T1, C10 and G11, using OpenMM-based molecular dynamics in ChimeraX 1.9.^50–53^ Docking was carried out with LeDock^54^ with the binding site defined by the minimum and maximum *x*, *y*, and *z* coordinates of the C3′ atoms of the G2·G9·G12·G18 tetrad. A total of 200 binding poses were generated and subsequently grouped into seven clusters using a 1 Å RMSD cutoff.

For MD, the geometry of DBA1 was optimized, and its single-point energy calculated using density functional theory with the B3LYP functional and 6-31G(d) basis set, as implemented in Orca 6.0.^55–65^ The resulting structure was converted to the Molden file format and used as input for two-stage restrained electrostatic potential (RESP) charge calculations in Multiwfn.^66,67^ RESP2 charges were then assigned to a charge file with Amber-compatible atom types, generated with antechamber.^68,69^ A prepin file describing the topology and parameters of DBA1 was generated using prepgen, and the corresponding force field parameters were obtained with parmchk2.^70^ The seven docking-derived complexes were prepared for MD in Amber24 using Leap.^70^ DNA was described using the OL21 force field,^71,72^ and DBA1 was parameterized with the files prepared above. Each system was solvated in a truncated octahedral OPC water box, with a 14 Å minimum distance from the solute to the box edge. Potassium and chloride ions (Li/Merz 12–6 set) were added to neutralize the system and reach an ionic strength of 100 mM.^73,74^ Corresponding ion numbers were determined using the SPLIT method.^75^

All minimization steps were performed on double-precision CPU (to avoid numerical overflowing) using pmemd, while MD production runs were executed with pmemd.cuda on an NVIDIA H100 PCIe Tensor Core GPU, hosted on the DOREMI CALI v3 cluster at the *Mésocentre de Calcul Intensif Aquitain* (Université de Bordeaux, France).^76–78^ A robust 10-step minimization and relaxation protocol was used to resolve any close contacts and stabilize system density prior to production.^79^ The final production runs were conducted for 50 ns under NPT conditions (1 atm, 300 K), with the first 10 ns discarded to allow final density equilibration. Simulations employed a Langevin thermostat (collision frequency: 5 ps^−1^), Monte Carlo barostat, a 9 Å non-bonded cutoff, and SHAKE constraints on bonds involving hydrogen. Different random seeds were used across runs to avoid synchronization artifacts.^80^

Trajectory analysis was carried out using cpptraj.^81^ First, the initial 10 ns and all water molecules were stripped from all trajectories, which were concatenated and clustered using the DBSCAN algorithm,^82^ based on the heavy atoms of DBA1, the bound tetrad guanines, and residues T1, C10, and G11. The distance cutoff (0.9 Å) was chosen based on the onset of plateauing observed in a *K*-distance plot generated in R 4.5.1 (Fig. S22A).^83^ Nine clusters were produced accounting for around 70% of the frames. In parallel, the DPeaks algorithm was also used,^84^ and four cluster centers were found by plotting a decision graph (Fig. S22C; distance cut-off: 2, density cut-off: 400). These clusters were very similar to the first 4 clusters obtained with DBSCAN, and were kept for visual analysis in PyMOL 3.1 (Schrödinger, LLC).^85^

### 2.3 Cellular assays

#### 2.3.1 Antiviral measurement

Infections were performed in a BSL-3 laboratory at the UB'L3 platform in Bordeaux (UAR CNRS 3427/Inserm US 005/Univ. Bordeaux). HIV-1 viral suspensions were obtained by coculture of MT4 cells (1.10^6^ ml^−1^) and H9 cells chronically infected by HIV-1Bru isolate (1.10^6^ ml^−1^).^86^ MT4 cells were obtained from the AIDS research and reference reagent program. H9 chronically infected cells were a gift from M. Ventura.^86^ After 48 h, the cell culture was centrifuged and the supernatant clarified by filtration on a 0.45 μm membrane before freezing. HeLa P4 cells used for infection experiments were a kind gift of P. Charneau.^87^ HIV-1Lai was obtained as previously described.^86^ HeLa P4 cells^87^ used for infection experiments were maintained in DMEM medium (Invitrogen) supplemented with 10% inactivated fetal calf serum (FCS) and 1 mg ml^−1^ geneticin (G418, Gibco-BRL). This cell encodes a Tat-inducible β-galactosidase gene driven by the HIV-1 LTR and linked to the expression of the viral Tat protein. HeLa P4 cells were seeded in a 96-well plate containing 10 000 cells per well for 24 h at 37 °C in a 5% CO_2_ atmosphere. After this period, compounds dissolved in DMSO (or DMSO as a solvent control) were added in duplicate 30 minutes prior to infection at various concentrations (50, 25, 12.5, 6.25, 3.1, 1.5, 0.8, 0.4, 0.2, 0.1 μM). The final DMSO concentration is maintained at 0.5%. Infection was carried out at a MOI of 1. The compounds were not removed by washing during the incubation period. After 24 h of culture, β-gal activity was quantified by adding 4-MUG mix (50 mM Tris–HCl, pH 8, 100 mM β-mercaptoethanol, 0.05% Triton X-100, 5 mM 4-MUG) to cells. Fluorescence associated with the reaction product was monitored 24 h after adding the 4-MUG mix using a Cytofluor-II plate reader (Applied Biosystems) with excitation/emission filters at 360/460 nm. The IC_50_ values were calculated using a non-linear regression model (log(agonist) *vs.* variable slope (four parameters)) with GraphPad Prism 9.5.1. The dose–response curves were fitted on a linear concentration scale. Bottom is equal to zero.

#### 2.3.2 Cell viability assays

The cells were seeded at a density of 10^4^ cells per well. After this period, the compounds (or DMSO as a solvent control) were added at various concentrations (50, 25, 12.5, 6.25, 3.1, 1.5, 0.8, 0.4, 0.2, 0.1 μM), maintaining a final DMSO concentration of 0.5%. Following 24 h of treatment, cell viability was assessed using the CellTiter 96® AQueous One Solution Cell Proliferation Assay System (G358A; Promega, Charbonnières-les-bains, France) after a 2 h incubation at 37 °C. Cell viability was evaluated at 492 nm by quantification of the formazan product which is proportional to the number of living cells using an Apollo LB 911 ELISA reader (Berthold technologies GmbH & Co. KG, Bad Wildbad, Germany).

## 3. Results and discussion

### 3.1 Synthesis and characterisation of dibenzoacridinium (DBA) derivatives

**Chart 1:**
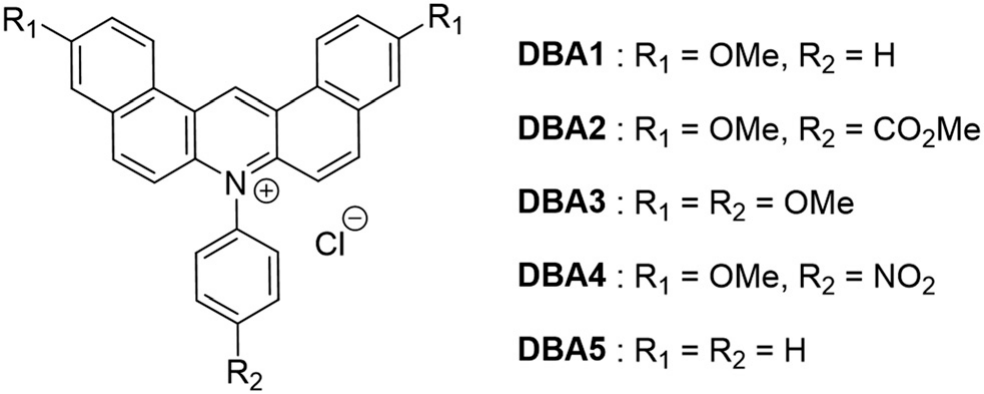
Molecular structures of the dibenzoacridinium derivatives.

**Scheme 1:**
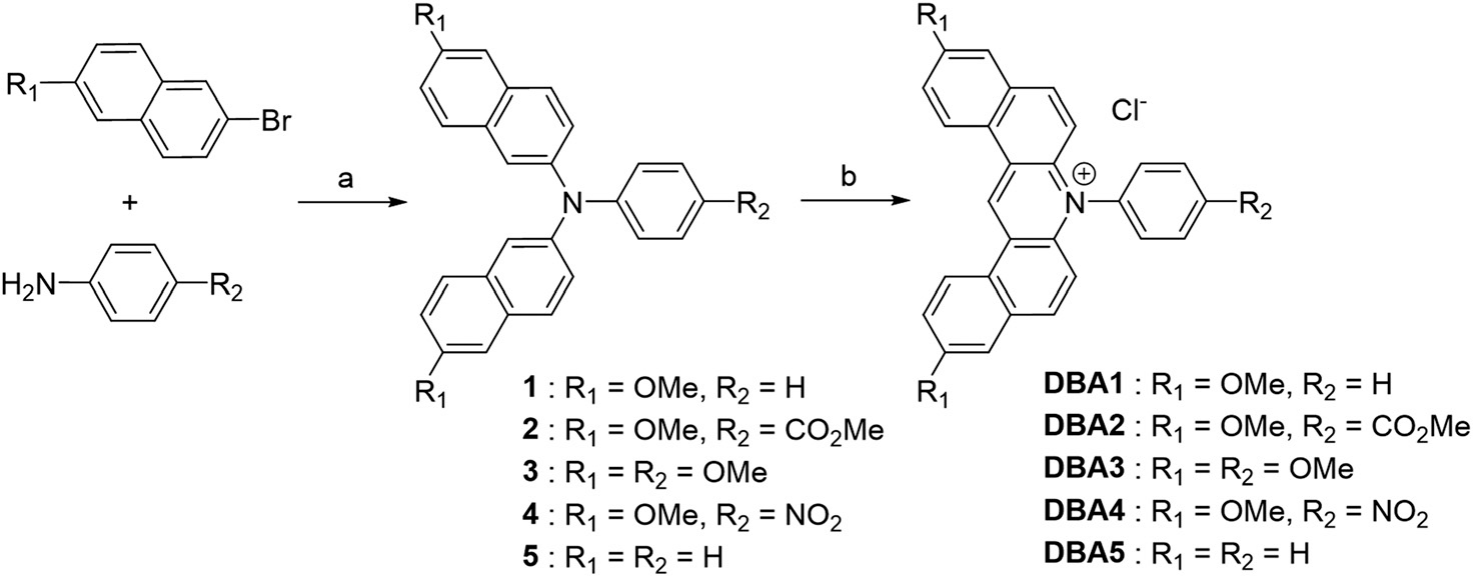
Synthesis pathway to the dibenzoacridinium derivatives. (a) Pd(OAc)_2_, P(*t*Bu)_3_, Cs_2_CO_3_, toluene, 110 °C, 3 days; (b) POCl_3_, DMF, 90 °C, 3 h.

We have synthesized a series of dibenzoacridinium derivatives (DBA1–5, Chart 1). The synthetic route towards the new ligands is illustrated in Scheme 1 (Material and methods and the detailed synthetic routes in the SI section). The dibenzoacridinium core was obtained from triarylamine precursors, which can be prepared in a single step. Specifically, aniline derivatives were reacted with two equivalents of (methoxy)bromonaphthalene in a Buchwald–Hartwig palladium-catalyzed cross-coupling reaction. This process led to *N*,*N*-dinaphthyl-phenylamine precursors 1–5 with high yields ranging from 60% to 87%. Subsequently, these arylamine precursors underwent a Vilsmeier-type reaction using phosphorus oxychloride in *N*,*N*-dimethylformamide (DMF), resulting in the formation of dibenzoacridinium derivatives (DBA1–5) with excellent yields between 61% and 92%. The five new ligands and all the intermediate compounds were fully characterized by ^1^H NMR, ^13^C NMR, UV, CD and HR-MS (Fig. S12–S21).

**Scheme 2:**
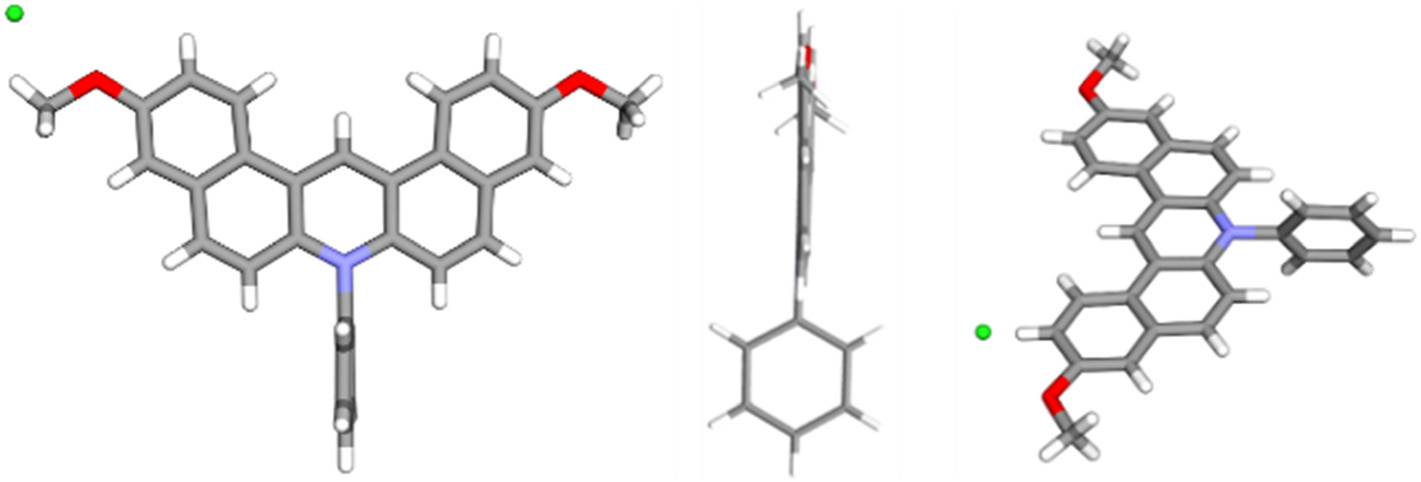
X-ray crystal structure of DBA1 (top, lateral and side views).

Interestingly the ^1^H-NMR spectra of the dibenzoacridinium derivatives show a characteristic peak in the range of 11.1–11.5 ppm. The deshielded singlet peak accounts for the proton at the center of the acridinium core, in *para* position with regard to the nitrogen atom. Single crystals of DBA1 were grown from a dichloromethane/hexane solvent mixture and the crystal structure was solved by X-ray diffraction analysis for further evidence of the new ligands' molecular structure (Scheme 2). As expected, the dibenzoacridinium core is composed of five aromatic rings (*ortho*–*meta*–*ortho* fused) that create an extended planar platform while the phenyl group linked to the nitrogen atom adopts a perpendicular orientation relative to the acridinium plane. The structure also confirms the presence of the chloride counter-anion. UV visible spectra as well as CD spectra of DBA1–5 were recorded and epsilons were calculated (Fig. S1 and Table S1). These derivatives present very interesting features regarding G4 recognition. Notably, the acridinium plane offers a large platform suitable for π–π stacking interactions with G-quartet planes. The perpendicular arrangement of the phenyl potentially provides additional interactions with G4 grooves or loops. At the same time, the phenyl group will also sterically prevent intercalation into canonical B-DNA duplexes thereby conferring G4 selectivity. Furthermore, the permanent positive charge in the center could interact with the carbonyl groups of the tetrad.

To experimentally validate these predicted features, we conducted a comprehensive G4-binding characterization. We first assessed the ability of all five DBA derivatives to bind and stabilize the primary G4 targets of therapeutic interest derived from the HIV-1 genome: LTR4, rLTR4, HIVpro1 and HIVpro2 all from the long terminal repeat region (Table 1), that have already been characterized in previous studies.^26,31,32,34^ The panel was also expanded to include three well-established model G4 sequences representing different topologies:^88^ Htel (human telomeric, polymorphic), TG4T (tetramolecular, parallel), and G4T4G4 (bimolecular, antiparallel). To assess G4 binding specificity, we also studied interactions with three self-complementary DNA duplexes: DS26 (26 bp), DK66, and DK100.^88^ Four complementary biophysical techniques were employed: circular dichroism (CD) spectroscopy to monitor structural changes, FRET-melting assays to quantify thermal stabilization (Δ*T*_m_), fluorescence quenching assays to determine binding affinity, and native mass spectrometry (ESI-MS) to assess binding stoichiometry and dissociation constants.

### 3.2 Analysis of DBA/G4 interaction by circular dichroism spectroscopy

**Fig. 2:**
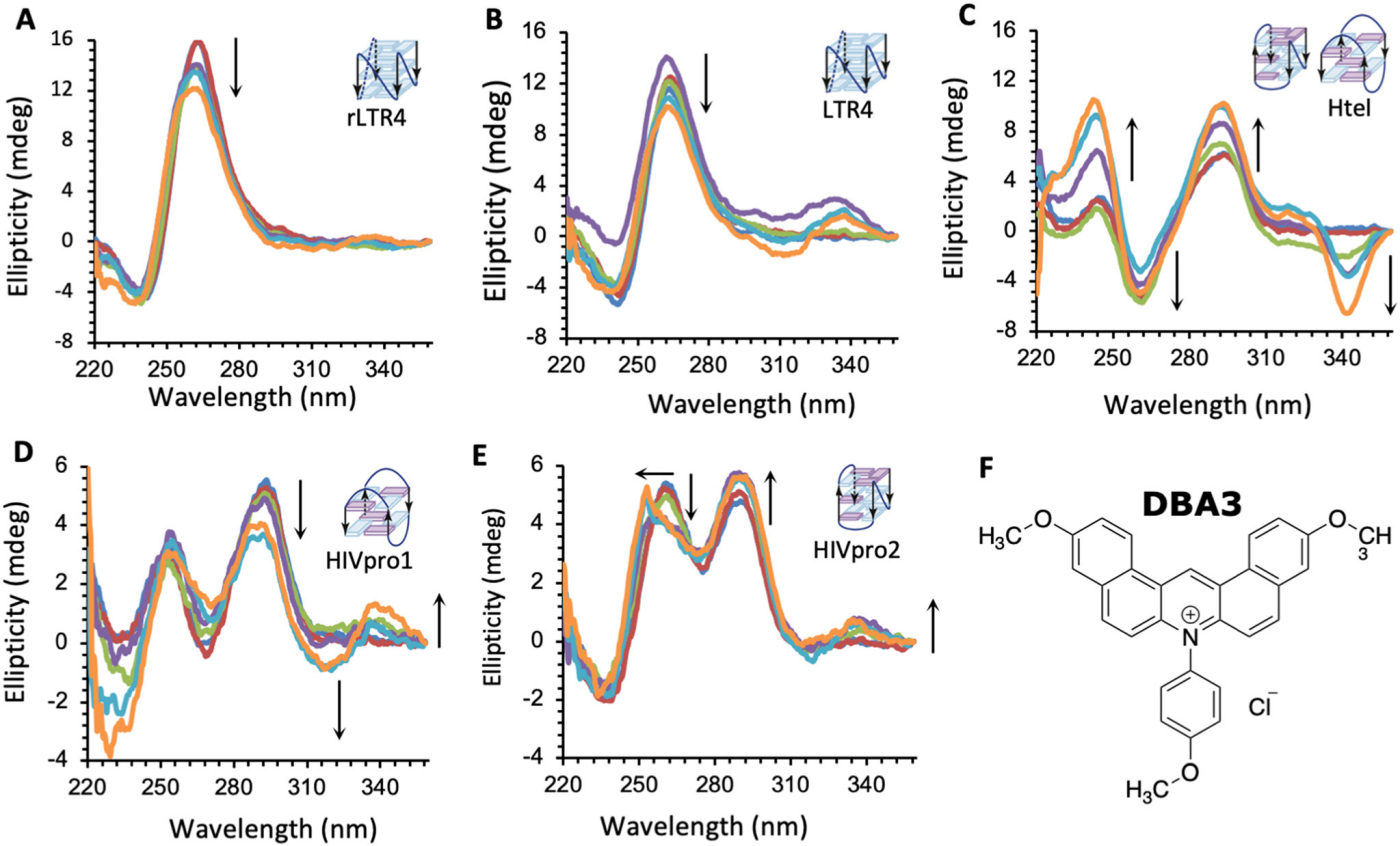
A–E) Titration of 5 different G4 topologies (rLTR4, LTR4, Htel, HIVpro1, HIVpro2) with DBA3. Oligonucleotides were prepared at 2.5 μM in 10 mM potassium cacodylate buffer pH 7.0 and 100 mM KCl (dark blue). DBA3 was added at 0.5 (red), 1 (green), 2 (purple), 3 (light blue) and 4 (orange) equivalents. The incubation time ranges between 1 and 5 minutes depending on starting time of spectra acquisition. The arrows highlight the change in intensity or wavelength of the peaks upon ligand addition. F) Chemical structure of DBA3.

We first explored the effect of the binding of each DBAs on RNA and DNA G4 structures using circular dichroism spectroscopy (CD). CD spectroscopy gives information on the G4 structures^89,90^ but can also reveal the binding effect of small ligands without requiring any labelling. Local conformational changes within the binding sites are induced by the ligands and can be detected through CD signal alterations. We conducted CD titration experiments using various G4 conformations derived from the HIV-1 genome for LTR4, rLTR4, HIVpro1 and HIVpro2 or from the human telomeric sequence (Table 1, Fig. S2 and 2). LTR4 and rLTR4 form parallel G4s with characteristic maxima at 290 nm and minima at 240 nm. HIVpro1 forms a two-tetrad antiparallel G4 with its characteristic maxima at 290 nm and 250 nm and minima at 268 nm and 230 nm. HIVpro2 forms a three tetrad hybrid G4 with its characteristic maxima at 290 nm and 265 nm and minimum at 230 nm. Htel forms a mixture of antiparallel and hybrid G4s. Interestingly, we observed very distinct modifications in CD spectra upon ligand addition depending on the G4 structures as well as the nature of the DBA derivatives. The results obtained for DBA3 are presented in Fig. 2 and the other DBAs are presented in Fig. S3–S6.

In the case of the rLTR4 parallel RNA G4, the binding of all DBAs including DBA3 induced a slight decrease of the CD peak at 263 nm (Fig. 2 and S3–S6). For LTR4 parallel G4, (the DNA version of rLTR4) DBA3 and DBA1 induced this time an increase of the signal at 263 nm upon addition of up to 2 equivalents. Then, DAB3 decreased the signal after addition of the third and fourth equivalents which was also accompanied by a slight broadening of the peak towards the highest energies. Interestingly, a positive induced CD signal around 340 nm was also observed for DBA3. Conversely, DBA2 and DBA5 induced a slight decrease of the CD peak at 263 nm (Fig. S4 and S6) as observed for the RNA G4. No change was detected for DBA4 (Fig. S5). In the case of the antiparallel DNA G4 (HIVpro1), DBA3 addition decreased the signal at 295 nm which was also accompanied by the emergence of a positive induced CD signal around 340 nm. DBA2, DBA4 and DBA5 also induced a decrease at both 245 nm and 295 nm. DBA1 was the only derivative that induced an increase of the signal at the same wavelengths.

For the hybrid DNA G4 (HIVpro2), DBA3, DBA4 and DBA5 induced a decrease at 245 nm and concomitant increase at 295 nm. For DBA3 this was also accompanied by a slight shift of the 245 nm peak as well as an increase of signal after adding 3 and 4 equivalents of DBA3. DBA1 and DBA2 induced a decrease of the signal at 245 nm. In the presence of Htel DNA G4, which is a mixture of different G4 conformations, all five DBAs behaved in a very similar way. Upon addition of the DBAs, a significant increase in the CD signal at both 245 nm and 290 nm is observed. At the same time, a negative induced CD signal emerged at around 320–340 nm after the addition of 1 to 4 equivalents of DBAs.

The different alterations of the CD spectra observed here strongly suggest that the DBA derivatives bind to the G4 structures. However, it is difficult to draw conclusions about the conformational modifications of the G4s induced by ligand binding. Indeed, since DBA derivatives present a broad UV absorbance band between 340 nm and 220 nm (Fig. S1), an induced CD signal coming from the ligand could appear in this region which also overlaps with the CD signature of each G4. Thus, a concomitant conformational change of the G4 and an induced CD from the DBAs could happen at the same time. Indeed, the significant modifications of the CD spectra of Htel G4s in the presence of DBAs very likely result from these two phenomena. However, the induced CD signal can also provide information about the binding mode of the ligands. Głuszyńska *et al.* proposed that a negative ICD, as observed here for Htel G4, may be the result of the stacking of the ligand on the G4 tetrad while a positive induced CD signal, as observed here for LTR4, might be the result of groove binding mode.^91,92^ Nevertheless, it has also been proposed that in the presence of B-DNA, a negative ICD may also result from an intercalating binding mode.^93^

### 3.3 Analysis by FRET-melting assay: DBA derivatives stabilize and bind specifically to G4s

**Fig. 3:**
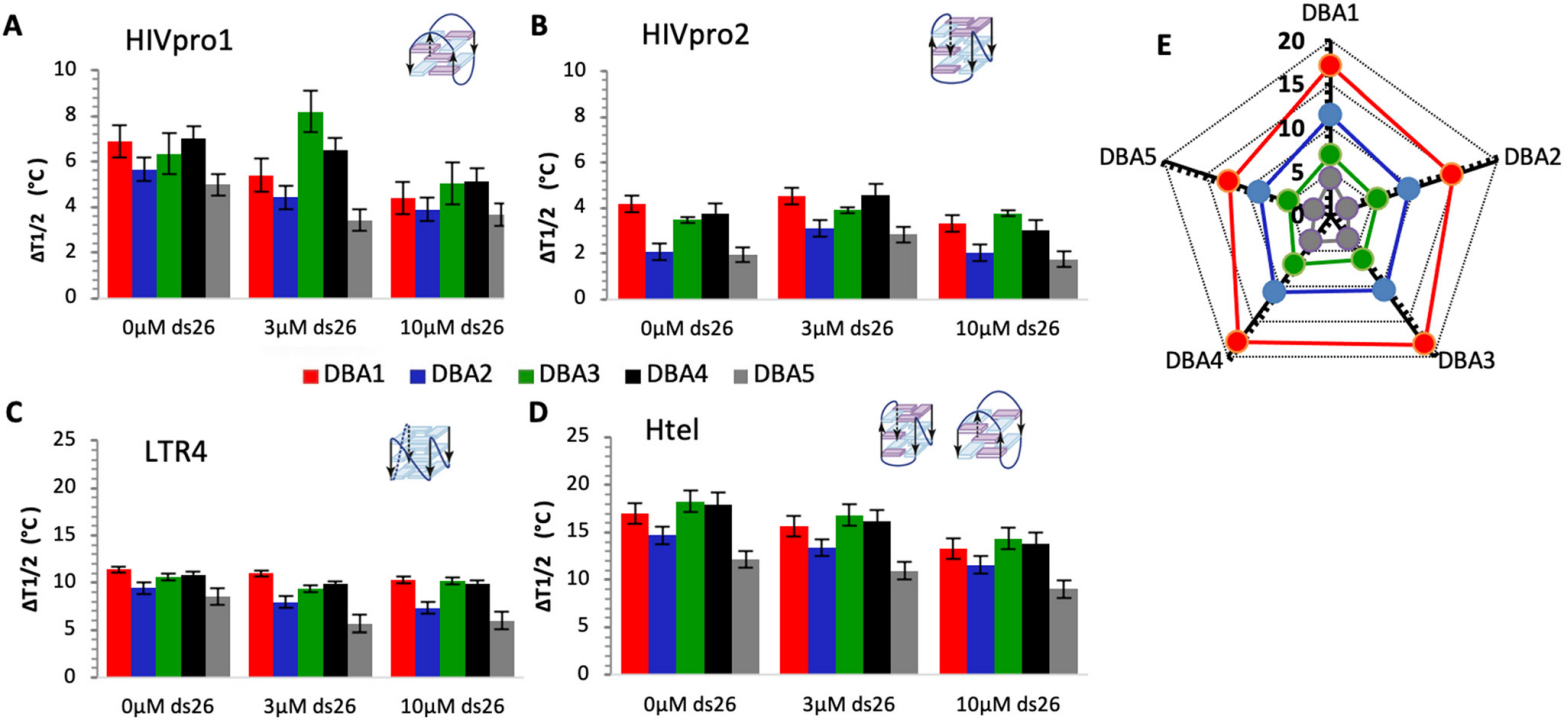
A–D) FRET-melting assays for HIVpro1-FT, HIVpro2-FT, LTR4-FT, and Htel-FT in the presence of DBA1–5 with different duplex competitor concentrations (0 μM, 3 μM and 10 μM ds26). The fluorescently labeled oligonucleotides were dissolved at a concentration of 0.2 μM in 10 mM lithium cacodylate buffer (pH 7.2) with 10 mM KCl and 90 mM LiCl. DBA1–5 were added at a final concentration of 1 μM. E) Radar plot representation that summarizes the FRET-melting results (Δ*T*_1/2_ (°C)) for each ligand in the presence of HIVpro1-FT (green), HIVpro2-FT (grey), LTR4-FT (bleu), and Htel-FT (red). Data are the results of at least three independent experiments in which each data point was performed in duplicate.

In this assay, we performed thermal denaturation experiments by heating and recording the fluorescence of DNA oligonucleotides doubly labelled with fluorescein and TAMRA fluorophores (Fig. 3 and S7). Ligand binding to the G4 stabilises the structure, resulting in an increase in the half-dissociation temperature of the structure (Δ*T*_1/2_). As a first approximation, the higher the Δ*T*_1/2_ (Δ*T*_1/2_ higher), the higher the affinity of the ligand for the target.^45^

We determine the stabilisation effects induced by DBA1–5 on HIVpro1, HIVpro2, LTR4 and Htel DNA G4s (Fig. 3). Overall, in the absence of a duplex competitor, we observed that DBAs better stabilise Htel G4 followed by LTR4 parallel G4 and HIVpro1 anti-parallel structure. The weakest effects were obtained for the HIVpro2 hybrid structure. By comparing DBA1 and DBA5 we could assess the influence of the methoxy group in the R1 and R2 positions. The Δ*T*_1/2_ values for DBA1 range from 4 °C to 17 °C across the four different DNA G4 conformations, indicating a higher affinity in the presence of this group compared to DBA5, where Δ*T*_1/2_ varies between 1.9 °C and 12 °C. Analysing the substitutions on the *N*-phenyl group appears to have minimal effect. For instance, DBA3, featuring an electron-donating substituent, exhibits similar Δ*T*_1/2_ values to DBA4, with an electron-withdrawing group (10.6 °C *vs.* 11 °C with Htel, 3.5 °C *vs.* 3.8 °C with HIVpro2, 6.3 °C *vs.* 7 °C with HIVpro1, and 18 °C for both with LTR4). However, DBA2, bearing an ester moiety on the *N*-phenyl position, demonstrates lower Δ*T*_1/2_ values compared to DBA3 and DBA4 (9.4 °C *vs.* 11 °C with Htel, 2.1 °C *vs.* 3.5 °C with Pro2, 5.6 °C *vs.* 6.3 °C with Pro1, and 15 °C *vs.* 18 °C with LTR4). This discrepancy might be attributed to the larger size of the substitution in DBA2, potentially leading to steric hindrance affecting the interaction with the G4. Thus, in terms of DNA G4 stabilization, the DBA ligands can be ranked as follows: DBA1 > DBA4 > DBA3 > DBA2 > DBA5 (Table S2).

Performing these experiments in the presence of a DNA duplex competitor (ds26) composed of 26 base pairs (Table 1) enables evaluation of the binding selectivity of the ligand for the G4 in competition with the duplex. Interestingly, in all cases the Δ*T*_1/2_ is mostly maintained and diminished by only 0–3 °C although 50 molar equivalents of competitor are used (600 equivalents when comparing the number of base-pairs^26^ with the number of tetrads^3^). The selectivity index indicates the proportion of stabilization maintained in the presence of the competitor (Table S2). Interestingly, this value ranged from 0.8 to 0.9 indicating that these ligands bind predominantly to the G4 structures.

### 3.4 Analysis by fluorescence quenching assay: DBA derivatives bind to RNA G4s with high affinity and specificity

**Fig. 4:**
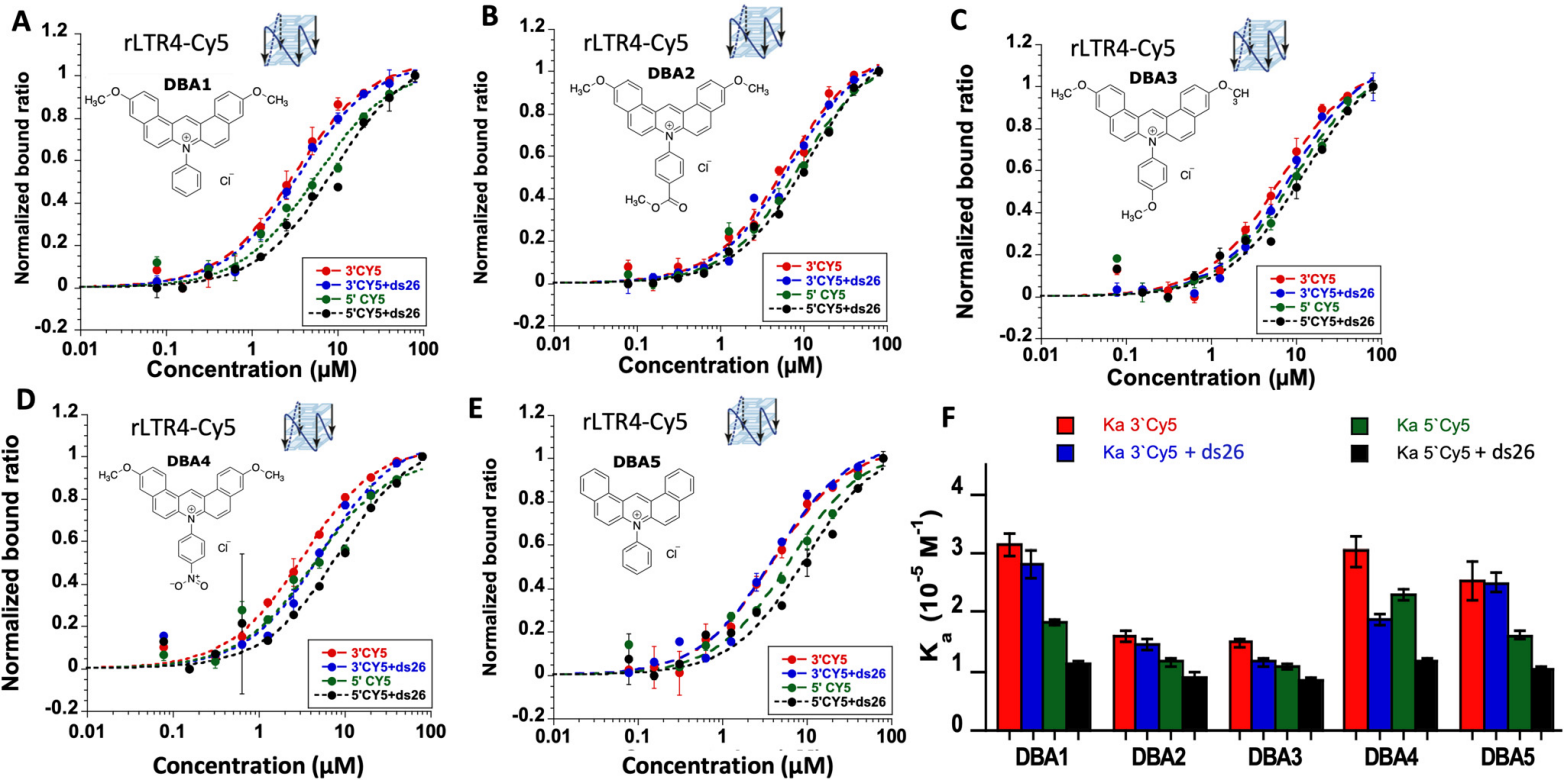
A–E. Results of the fluorescence quenching assay for 3′ and 5′ Cy5 labeled rLTR4 with DBA1–DBA5, in the presence or absence of duplex competitor ds26 at 10 μM. F. Histogram showing the association constant of 3′ and 5′ rLTR4-Cy5 calculated with each of DBA1–DBA5 and how it is affected by the competitor (ds26). The fluorescently labeled oligonucleotides were dissolved at a concentration of 10 nM in 10 mM lithium cacodylate buffer (pH 7.2) with 10 mM KCl and 90 mM LiCl. DBA1–5 were added at a final concentration ranging from 0 to 80 μM. Data are the results of at least three independent experiments in which each data point was performed in duplicate.

We then addressed the binding of the DBA series to the rLTR4 RNA G4. The FRET melting assay was not appropriate for investigating ligand binding to this RNAs due to its high thermal stability (*T*_m_ > 72 °C).^26^ We have therefore used a ligand-induced fluorescence quenching assay (FQA) to analyse the interaction of the DBAs with either 5′ or 3′ fluorescently labelled RNAs (Fig. S8). Since planar G4-ligands generally stack on an accessible G-quartet,^94–96^ we expect the DBAs to also recognize G4s by stacking interactions. To conduct a fluorescence quenching experiment, a CY5 fluorophore is attached on the 3′ or 5′ end of the RNA. Le *et al.* demonstrated that the binding of a ligand to a terminally tagged G4 caused proximal quenching of the dye's fluorescence, which was recovered by adding an excess of non-labelled G4 competitor.^47^ Thus, upon interaction of the ligand with the G-quartet, the ligand induces the quenching of the fluorescence emission. This test enables determination of the apparent association constants (*K*_a_) of each DBA with the 5′ or 3′ quartet of the G4 structure (Fig. 4, Table S3). For instance, DBA1 and DBA4 exhibited the highest affinity towards the RNA G4, with dissociation constants (*K*_d_) ranging from 3.2 μM to 5.5 μM. Notably, there was a discernible binding preference for the G-tetrad at the 3′ end over the 5′ end among all DBAs. A similar difference in G-tetrad binding affinities has previously been observed for TMPyP4 and PhenDC3.^47^

Regarding G4 binding specificity, we performed the FQA in the presence of high excess of ds26 double helix competitor as described in the FRET assay. DBA1–2–3–5 demonstrated notable selectivity towards RNA G4s over competing duplexes, particularly accentuated at the 3′ end. Analysis of the specificity index indicated that DBA4 exhibited the lowest specificity index within the series at 0.6. At the 5′ end, binding was much less selective (specificity index between 0.5 and 0.8) confirming the lower affinity measured without a competitor. Interestingly, while the trend in *K*_a_ values did not precisely align with observations from FRET melting assays, it was notable that the methoxy substitution had minimal impact on *K*_a_ with DBA1 and DBA4 yet maintaining the highest affinity consistent with FRET results. The ranking of the DBA according to their apparent *K*_a_ for RNA G4s is as follows: DBA1 > DBA4 > DBA5 > DBA2 > DBA3 (Table S3).

### 3.5 Analysis by native ESI-MS: DBA derivatives preferentially bind to rLTR4 RNA G4 over DNA G4s

We further analysed the formation of the DBA/G4 complexes by native electrospray ionization mass spectrometry (ESI-MS). We initially attempted to analyze DBA binding to the primary G4 targets from HIV-1 (HIVpro1, HIVpro2, LTR4 and rLTR4 RNA). However, while rLTR4 RNA gave good quality spectra, HIVpro1, HIVpro2 and LTR4 DNAs did not produce sufficiently interpretable signals under ESI-MS conditions (100 mM ammonium acetate), likely due to their structural complexity under these conditions. We therefore selected alternative model G4 structures that exhibit well-defined ESI-MS behaviour: TG4T DNA G4 (parallel topology), the human telomeric Htel DNA G4 (polymorphic), and G4T4G4 DNA G4 (antiparallel topology). These sequences span the major G4 topological classes and provide a representative assessment of DBA binding across diverse G4 architectures. We also included two DNA duplex controls (DK100 and DK66) to evaluate binding selectivity. This ESI-MS approach enables direct determination of binding stoichiometry, quantification of each species in solution, and calculation of dissociation constants (*K*_d_) from the relative abundances of free and bound forms.^88,97^

**Fig. 5:**
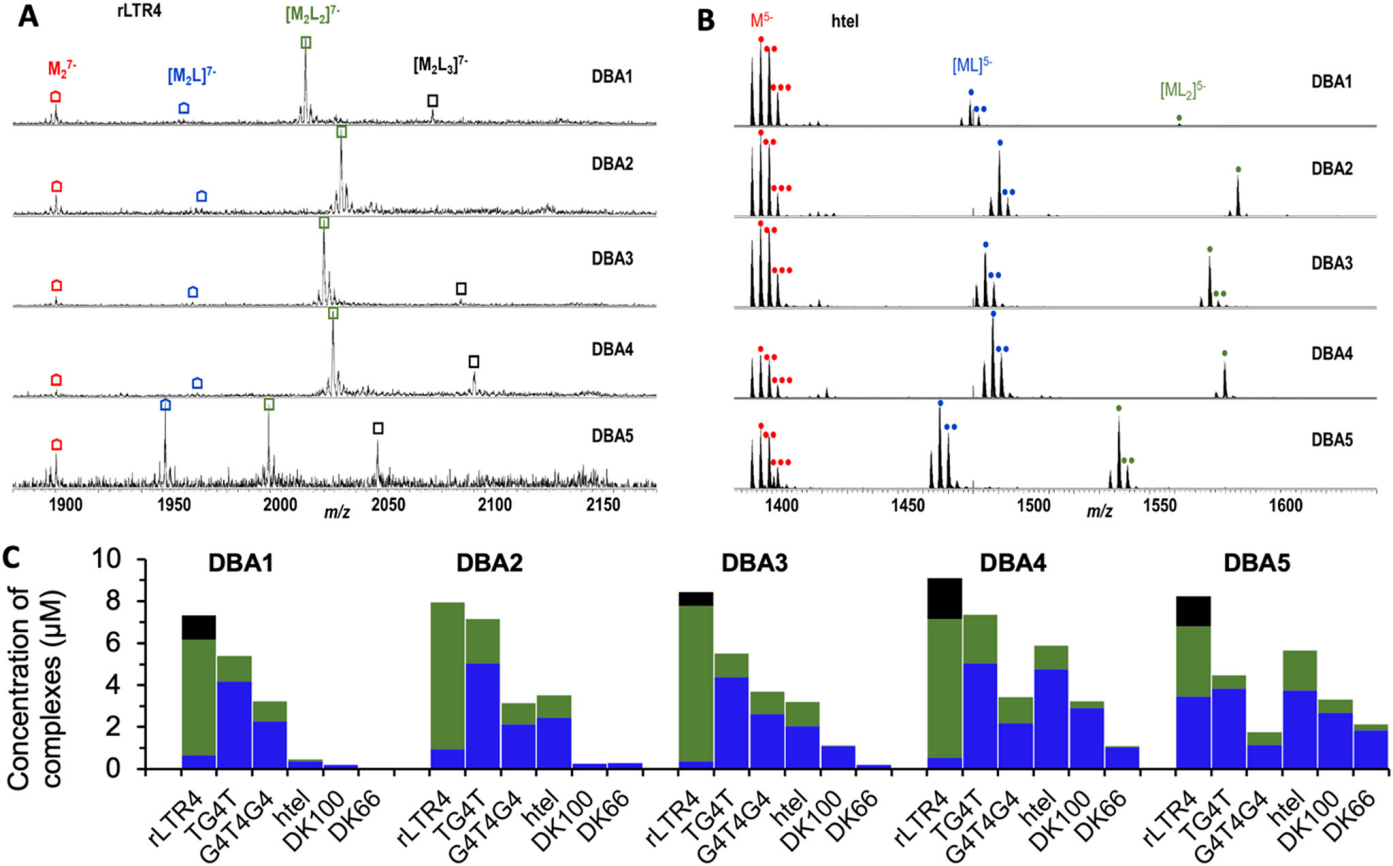
A) Native electrospray mass spectra of complexes of rLTR4 and (B) Htel at 10 μM with DBA1–5 (20 μM) in 100 mM ammonium acetate, highlighting the −7 and −5 charge state, respectively: M = oligonucleotide monomer, L = ligand. For rLTR4, the most abundant ammonium stoichiometry is in green (M2L2) or black (M2L3) with □ (4 NH_4_^+^) and red (M2) or blue (M2L) with 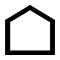 (5 NH_4_^+^). For Htel, the most abundant ammonium stoichiometry is labelled in red (M), blue (ML) and green (ML2) with • (1 NH_4_^+^), •• (2 NH_4_^+^) and ••• (3 NH_4_^+^). C) Concentrations of 1 : 1 (blue), 1 : 2 (green) and 1 : 3 (black) complexes of all six oligonucleotides in the presence of DBA1–5 ligands in 100 mM ammonium acetate.

In the ESI-MS conditions, the rLTR4 G4 (10 μM) forms a dimeric structure. In the presence of two equivalents (20 μM) of DBA1 and DBA3–4–5, we detected rLTR4/DBA complexes with 1 : 1, 1 : 2 and 1 : 3 stoichiometries, while only 1 : 1 and 1 : 2 complexes were observed for DBA2 (Fig. 5A). Among the six DNA and RNA tested here (Fig. 5B, S9 and S10) only rLTR4 presented 1 : 3 G4/DBA complexes. In all cases but one, the 1 : 1 stoichiometry represents the major species in solution while for rLTR4 it is the 1 : 2 (green). Indeed, concerning the Htel monomeric G4 structure DBA1–5 are able to form 1 : 1 and a 1 : 2 complexes while no 1 : 3 complexes were detected (Fig. 5B). Interestingly, for DBA/Htel complexes the major G4 conformation is the one containing one ammonium, *i.e.* composed of two tetrads, while the two or three ammonium conformations are the most represented for the free Htel G4. For TG4T tetramolecular G4 or G4T4G4 bimolecular G4 only 1 : 1 and 1 : 2 complexes were detected (Fig. S9A and S10A). Notably, when comparing the amounts of G4/DBA complexes formed in each solution (Fig. 5C), we can observe a preferential binding of the DBAs for the parallel rLTR4 and TG4T G4s while the antiparallel G4T4G4 and the polymorphic Htel presented globally much lower amounts of complexes. Very interestingly, almost no complexes were detected for DBA1–3 when incubated with DK66 or DK100 duplexes highlighting the high binding specificity of these compounds (Fig. S11). Conversely, significant amounts of duplex/DBA4–5 complexes were detected suggesting a lower G4 binding specificity for these two derivatives. The calculation of the dissociation constant *K*_d_ confirmed an overall preferential binding of the DBAs toward rLTR4 RNA with *K*_d2s_ ranging from 0.14 μM for DBA3 to 10 μM for DBA5. The weakest binding was obtained for the G4T4G4 antiparallel G4 and Htel sequence with *K*_d1s_ ranging from 11 μM for to 500 μM (Table 2). Notably, the *K*_ds_ for DK66 and DK100 duplexes ranged from 40 μM to 4.8 mM confirming the weak binding properties of the DBAs for the duplex structures. In the case of rLTR4, the ranking of the DBA according to their *K*_d2_ is as follows: DBA3 > DBA1 = DBA4 > DBA2 > DBA5.

**Table 2:** Native mass spectrometry analysis

	*K*_d_ (μM)	DBA1	DBA2	DBA3	DBA4	DBA5
rLTR4	*K*_d1_	20.20	10.91	12.18	10.38	4.99
	*K*_d2_	0.55	0.69	0.14	0.50	10.03
	*K*_d3_	22.78	*	30.97	21.29	1.47
TG4T	*K*_d1_	14.69	6.09	13.62	5.42	21.57
	*K*_d2_	45.97	25.93	52.19	22.66	89.36
G4T4G4	*K*_d1_	47.13	51.17	36.88	46.16	128.56
	*K*_d2_	38.63	33.63	37.02	26.61	33.43
Htel	*K*_d1_	497.43	41.05	52.44	11.21	14.47
	*K*_d2_	102.74	35.29	27.25	55.57	24.66
DK100	*K*_d1_	1027.52	833.14	155.63	38.42	40.04
	*K*_d2_	*	*	617.56	141.80	67.70
DK66	*K*_d1_	4833.59	711.55	1071.92	165.13	75.39
	*K*_d2_	*	*	*	308.22	117.48

As a result, DBAs bind to G4s with high affinity and specificity. Indeed, FRET-melting competition assays and fluorescence quenching assays (FQA) were conducted in the presence of high excess of unlabeled duplex DNA (ds26) as a competitor. In both cases, the stabilization or displacement effects of DBA ligands on G4 targets were only slightly affected by the presence of duplex DNA, indicating a high level of selectivity for G4 structures and weak binding to classical Watson–Crick duplex forms. Strikingly, mass spectrometry data indicate that DBA4 and DBA5 bind to double-stranded DNA, whereas duplex competition experiments in FRET suggest significant G4 selectivity. These discrepancies between native MS and FRET-melting results may arise from differences in the experimental protocols. In particular, DNA concentration differs substantially (10 μM in MS *versus* 0.2 μM in FRET-melting), as does ligand concentration (20 μM in MS *versus* 1 μM in FRET-melting). The higher DNA and ligand concentrations used in MS experiments may favour lower-affinity or nonspecific binding events that are less apparent under lower-concentration conditions of the FRET-melting assay. In addition, the ionic environment differs between the two techniques, with MS experiments performed in ammonium acetate buffer and FRET assays in KCl-containing buffer. Such differences may also influence G4 folding equilibria and ligand binding behaviour. Nevertheless, the affinity differences remain significant: for rLTR4 G4, *K*_d2_ values are 0.5 μM for DBA4 and 10 μM for DBA5, whereas binding to the DK100 duplex is markedly weaker (165 μM and 75 μM, respectively). Thus, despite methodological differences, MS data still indicate a clear preference for G4 over duplex DNA. These data also revealed that the affinity of DBA1 for G4 structures is approximately 1000 times higher than for duplex DNA, confirming the weak interactions with non-G4 nucleic acids.

Overall, DBA derivatives behave as broad G4 ligands capable of recognizing multiple G4 topologies, whether of viral or cellular origin, a characteristic typical of first-generation planar cationic scaffolds that will require further structural optimization to enhance viral selectivity. They nevertheless exhibit a marked selectivity over duplex DNA, as reflected by significantly stronger stabilization and binding affinities for G4 structures.

### 3.6 Analysis by NMR chemical shift perturbation and molecular docking

**Fig. 6:**
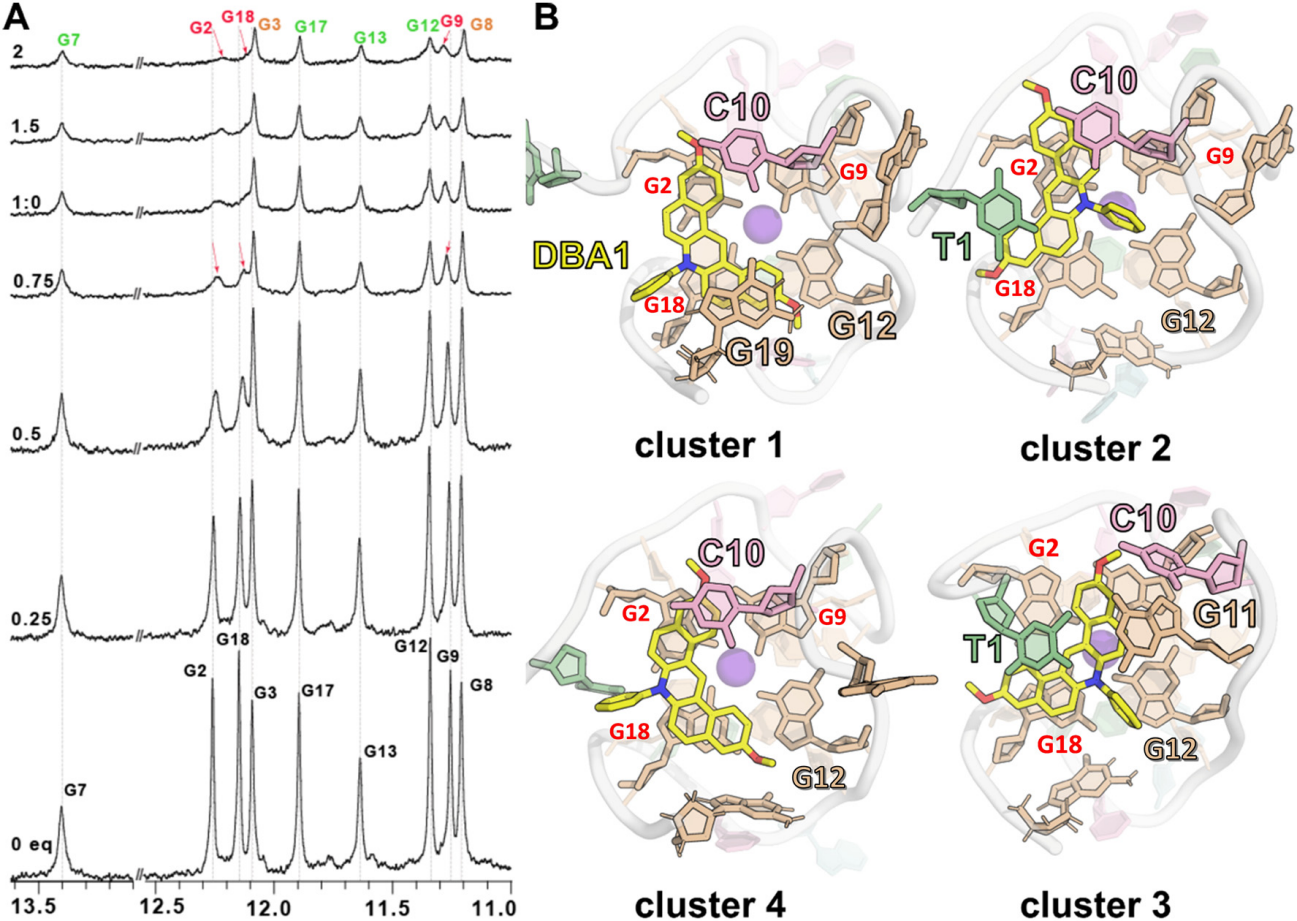
A. 1D-^1^H-NMR titration of HIVpro1 G4 with DBA1. Overlay of imino proton spectra (11.0–13.5 ppm) recorded upon incremental addition of DBA1 to the folded HIVpro1 G4 dissolved at a concentration of 150 μM in a 90% H_2_O/10% D_2_O buffer composed of 20 mM potassium phosphate and 70 mM KCl. Spectra correspond to molar ratios of DBA1 : G4 from 0 to 2.0 equivalents, as indicated on the left. Experiments were recorded at 25 °C. Guanine imino resonances are labeled according to their position in the G4-forming sequence. B. Top view of the binding site obtained by a combination of docking and unrestrained molecular dynamics, from which four representative structures were extracted. DBA1 is shown in yellow and colored by heteroatom, guanines in brown, cytosines in pink, thymines in green and adenines in blue, the backbone is depicted as a white ribbon, and K^+^ cations as purple spheres.

We then investigated the G4 recognition mode of the DBA1 ligand to the HIVpro1 G4 structure (Fig. 6). The HIVpro1 sequence adopts a well-defined, antiparallel two-tetrad G4 structure stabilized by a Watson–Crick (WC) base pair involving G7 and C4 residues.^31^ The G7–C4 base pairs stacks on the upper tetrad that is composed of G17, G13, G3, and G8, while the bottom tetrad includes G2, G9, G12 and G18. In order to determine the binding site of DBA1, we performed 1D-^1^H NMR titration experiments monitoring the imino proton region (11.0–13.5 ppm) (Fig. 6A). Upon gradual addition of DBA1 (0 to 2.0 molar equivalents), a general decrease in peak intensities accompanied the titration, likely due to intermediate chemical exchange on the NMR timescale or increased conformational flexibility induced by ligand binding. Yet, clear and progressive chemical shift perturbations (CSPs) were observed for specific guanine residues. Notably, G2 and G18—components of the bottom tetrad—exhibited significant upfield shifts starting at low ligand concentrations (≥0.5 eq.) accompanied with a complete disappearing of the peaks at 2 eq. while G9 showed a clear downfield shift. G2, G18 and G9 are the most affected residues suggesting that the binding occurs directly at this tetrad interface. Interestingly, G12 belongs to the same tetrad but is not affected by the interaction suggesting that the binding region is restrained to G2, G18 and G9 residues. Perturbations only slightly extended to G3 and G8 (from the top tetrad), consistent with a conformational response propagating through the G4 scaffold while no perturbation was observed for G13 and G17 (from the top tetrad) as well as G7 involved in the base-pair. Saturation was achieved around 1.5–2.0 equivalents, indicating a specific, high-affinity interaction. As a result, DBA1 selectively binds the bottom tetrad of the HIVpro1 G4 and induces local rearrangements around G2, G18 and G9 residues.

We next combined molecular docking and molecular dynamics (MD) simulations to investigate the potential binding modes of DBA1 to HIVpro1 (Fig. 6B). NMR titration data suggest that DBA interacts with the G2·G9·G12·G18 quartet; however, its binding orientation remains unclear. Docking allows the exploration of diverse binding geometries on the tetrad, however, because of the presence of the loop residues, this region is not readily accessible for ligand binding. Thus, based on the HIVpro1 structure,^31^ we i) exposed the bottom G2·G9·G12·G18 tetrad by flipping sterically hindering residues using OpenMM-based dynamics,^53^ ii) docked DBA1 onto the resulting structure, with no bias regarding its position on the tetrad, generating a diverse ensemble of binding poses grouped into seven clusters, and iii) subjected the top-scoring pose from each cluster to MD simulations using Amber.^79^ Each of the seven initial complexes underwent a 50 ns production run, allowing binding-site residues to establish interactions with DBA1 that are not captured by docking alone. The resulting trajectories were then clustered based on binding-site geometry, yielding four representative models that reflect the most probable binding modes.

The simulations revealed two distinct binding orientations of DBA1 across four major conformational clusters (Fig. 6B). In the first orientation (clusters 1: 32% of trajectory frames and cluster 4: 8%), the crescent-shaped DBA1 adopts a geometry that maximizes π–π stacking with the G-tetrad plane. The planar acridinium core aligns closely with guanines G2 and G18, while the perpendicular phenyl substituent occupies the groove formed by both HIVpro1 termini. G9 and G12 remain largely unengaged. The two clusters differ in DBA1's lateral positioning along the tetrad: in cluster 1, DBA1 shifts toward G12, enabling additional stacking with the 3′-terminal G19, whereas in cluster 4, DBA1 shifts toward G9, promoting stacking with the loop residue C10. In the second orientation, clusters 2 (32%) and 3 (28%) exhibit an inverted binding mode in which DBA1 is flipped ∼180° relative to orientation 1. The positively charged acridinium nitrogen is now positioned directly above the central ion channel, resulting in reduced π–π stacking with the tetrad; DBA1 engages only weakly with either G2 (cluster 2) or G18 (cluster 3). Instead, this orientation promotes stacking interactions with the 5′-terminal T1 residues. In cluster 2, DBA1 also stacks with C10, while in cluster 3, C10 adopts an alternative conformation, stacking on G11, which repositions atop DBA1 to form a well-structured lateral loop.

The binding geometries observed in cluster 1 and 4, featuring extensive tetrad stacking and groove positioning of the phenyl group, are strongly supported by the NMR titration experiments. The chemical shifts of G2 and G18 are substantially perturbed upon DBA1 binding, consistent with their direct π–π stacking interactions with the acridinium core. In contrast, G9 exhibits only weak chemical shift changes and G12 remains essentially unaffected, confirming minimal engagement with these residues as predicted by these binding modes.

### 3.7 Assessment of HIV-1 replication inhibition by DBAs

**Fig. 7:**
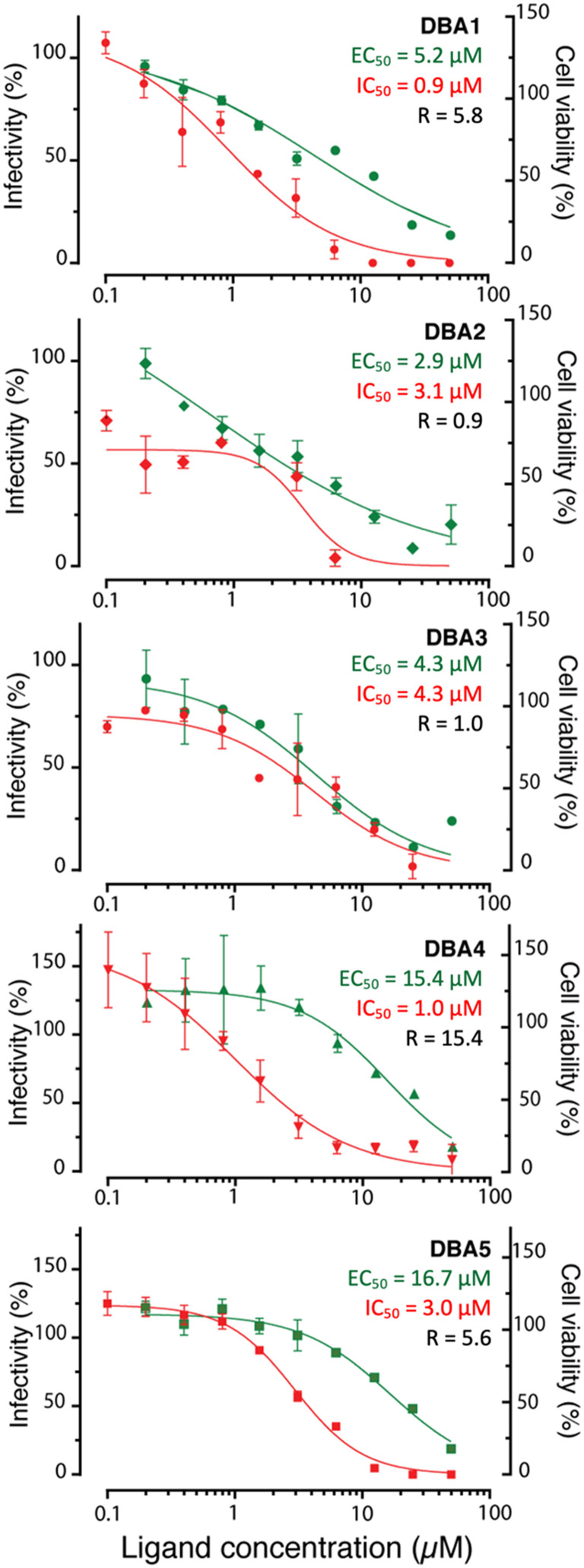
Antiviral effect of DBA1–5 derivatives. Red curves show infectivity inhibition (left axis) as a function of DBA concentration. Infection was performed with native HIV-1 in a BSL3 laboratory. The G4 ligands were added to the cells 30 minutes before infection. IC_50_ (half maximal inhibition concentration) values are provided in red. Green curves show the cell viability rate (right axis) as a function of DBA concentration. Increasing concentrations of DBA were added to the cells for 24 h, then cell viability was evaluated by quantification of the formazan product. EC_50_ (half maximal effective concentration) values for cell viability are provided in green. *R* = (EC_50_/IC_50_). Data are the results of at least three independent experiments each performed in duplicate.

Several G4 ligands, including porphyrins,^26^ have previously been shown to inhibit HIV-1 replication. We therefore anticipated that DBAs may also have anti-HIV-1 properties. In order to evaluate their antiviral effects, DBAs were tested in a dose–response experiment with native HIV-1 virus in a BSL-3 laboratory (Fig. 7) which allowed measurement of antiviral IC_50_ values for DBA1–5. HeLa P4 cells encoding a Tat-inducible β-galactosidase reporter gene were used for this assay. The expression rate of the reporter gene is directly correlated to the level of expression of the native viral Tat protein produced by the virus. In parallel, we also evaluated their effects on HeLa P4 cell viability which allowed the measurement of cytotoxic EC_50_ values for each ligand.

All of the tested compounds were able to reduce the HIV-1 infectivity with apparent IC_50_ ranging from 0.9 μM (DBA1) to 4.3 μM (DBA3) (Fig. 7). However, at the same time DBAs also showed cytotoxic effects on the HeLa P4 cells with EC_50_ ranging from 2.9 μM to 16.7 μM. In the particular case of DBA3 the cell viability and viral infectivity curves are concomitant with a ratio between the EC_50_ of cell viability and IC_50_ of inhibition equal to 1. The observed viral inhibition may actually be related to cell toxicity effects. IC_50_ is not measurable for DBA2 as a clear upper plateau near 100% is not reached, and a sigmoidal transition between the upper and lower plateau is not well-defined. In addition, cytotoxicity occurs within the same concentration range. For DBA1, 2 and 3, the IC_50_ values must be interpreted with caution as the inhibition curves do not meet the criteria expected for an antiviral response curve, and the cytotoxicity overlaps with antiviral activity. For these derivatives, the antiviral effect cannot be clearly separated from toxicity, and their biological profile likely reflects a strong contribution of nonspecific cellular effects.

In contrast, for DBA4 and 5, these ratios EC_50_/IC_50_ range between 5.6 and 15.4 clearly demonstrating a viral inhibition that is unrelated to cell toxicity. Interestingly, antiviral IC_50_ values for DBA4 and 5 (1 and 3 μM) are clearly separated from cytotoxic EC_50_ values (15.4 and 16.7 μM). Thus, antiviral inhibition occurs at concentrations below those inducing substantial loss of cell viability. Indeed, at 1.56 μM of DBA4 and DBA5, corresponding to 100% cell viability, viral infectivity was still reduced by approximately 20% and 40%, respectively. Although the magnitude of inhibition at this concentration is moderate, these data demonstrate that antiviral activity is detectable in the absence of measurable toxicity. This supports the conclusion that the observed antiviral effects cannot be solely attributed to nonspecific cytotoxicity. It is also important to note that the duration of infection is short: 24 hours, and that the data presented are valid only under the conditions of the experiment. The HeLa P4 reporter model is based on LTR expression following integration, rather than on the measurement of viral RNA produced in the supernatant, which would require a longer incubation period to allow for the production of new viral particles. Consequently, the infection can be measured shortly after infection. This allows for a shorter exposure time to the molecules. However, in our conditions, a fairly high MOI is required in order to obtain a detectable signal. Nevertheless, a longer exposure time could also provide access to additional information regarding cytotoxicity or antiviral activity.

G4-targeting ligands are also known to exhibit antiproliferative activities due to their ability to interact with G4 structures present in cellular genomes (reviewed here^98^). In particular, stabilization of cellular G4s—such as those located at telomeres or within oncogene promoters—can alter cell proliferation and viability. This is well illustrated by several archetypal G4 ligands: BRACO-19,^99^ an acridine derivative that binds telomeric G4s and induces telomere dysfunction and cellular senescence; TMPyP4,^100^ which inhibits telomerase activity and downregulates oncogenes such as c-MYC, hTERT, and KRAS; and RHPS4,^101^ which induces telomere damage and apoptosis in multiple cancer models. In this context, DBA derivatives behave as broad G4 ligands capable of recognizing multiple G4 topologies from both viral and cellular origins, while maintaining a clear selectivity over duplex DNA. While DBAs are not able to discriminate between cellular and viral G4s, the observed antiviral effect may be partly explained by the higher accessibility or availability of viral G4 structures during active viral replication compared to cellular G4s. Consistently, DBA1, 2 and 3 display overlapping cytotoxic and antiviral effects, preventing a clear distinction between the two. In contrast, DBA4 and DBA5 retain measurable antiviral activity at concentrations where cell viability remains high or unaffected (*e.g.*, up to 100% viability at 1.56 μM with residual antiviral effects), indicating that their activity cannot be solely attributed to cytotoxicity. Nevertheless, partial modulation of cellular permissiveness to infection through interactions with host G4s cannot be excluded. Altogether, these results highlight both the potential and the current limitations of this first-generation scaffold and underscore the need for further optimization to enhance viral selectivity and improve the therapeutic window.

### 3.8 Effects of R1 and R2 substitutions on the biophysical and biological properties of DBAs

**Fig. 8:**
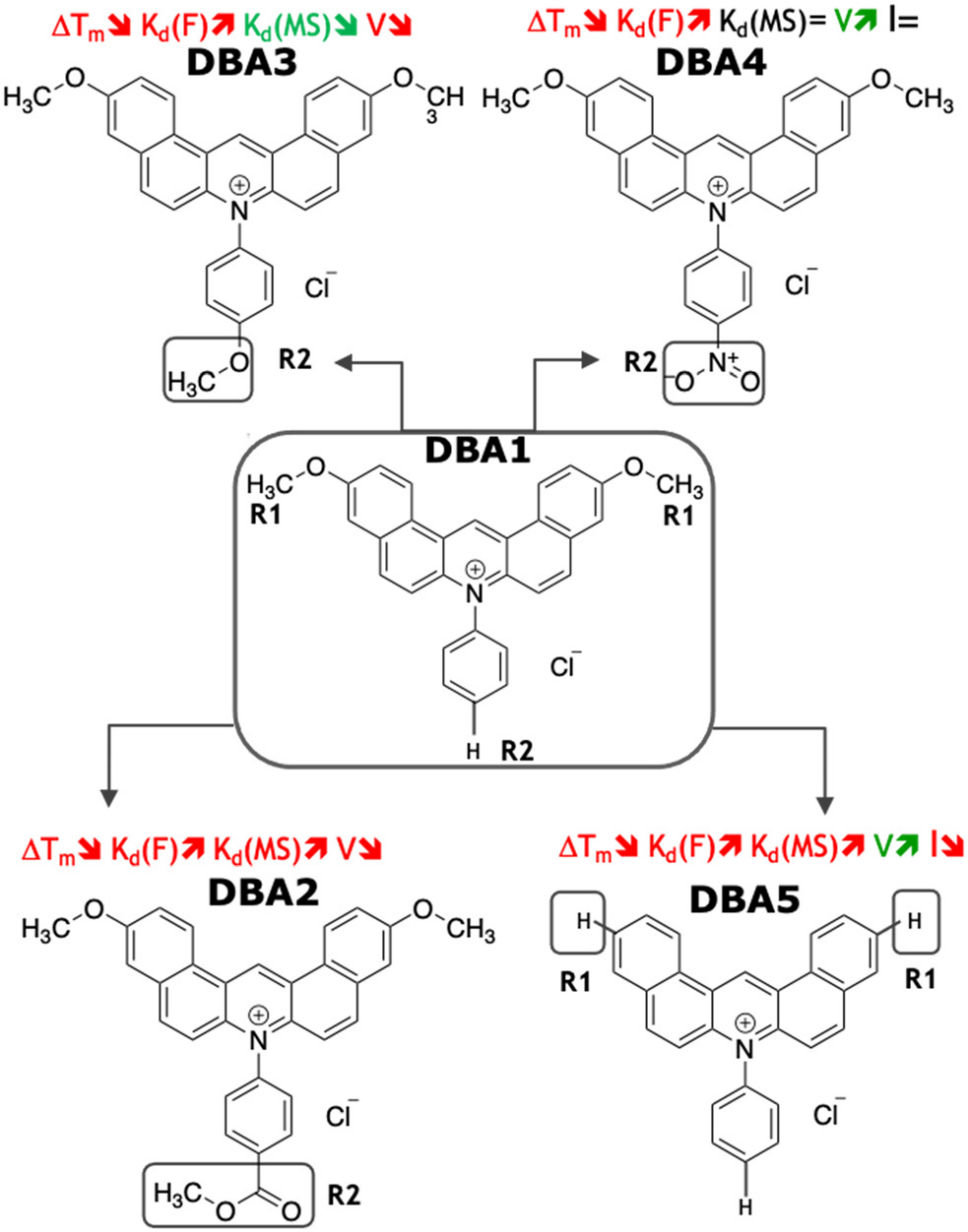
Summary of the effects of R1 or R2 substitutions as compared to DBA1 derivative. Positive effects are in green and negative effects in red. Increase (↗), decrease (↘) or no effect (=) on thermal stabilisation (Δ*T*_m_), *K*_d_ measured by FQA (*K*_d_(F)), *K*_d_ measured by ESI-MS (*K*_d_(MS)), cell viability (*V*), and HIV-1 inhibition (*I*).

We have assessed here the biophysical and biological properties of the DBA derivatives as G4 binders and anti-HIV molecules. Based on these results, we were able to identify a number of effects for each pharmacological modulation of this family of compounds (Fig. 8).

Concerning the G4 binding properties, and taking DBA1 as a reference, we found that all R2 or R1 substitutions had detrimental effects as measured by FRET melting, FQA or ESI-MS. Indeed, in DBA5, the removal of the OMe groups in the R1 position decreases the Δ*T*_1/2_ and increases the *K*_d_ values. Similarly, the –NO_2_ (DBA4), –OMe (DBA3) or –CO_2_Me (DBA2) substitutions in R2 positions had similar effects, although a slight discrepancy between the *K*_d_ from FQA and MS can be observed. Interestingly, we also observed that a given group can have opposite effects when added either in R1 or R2 positions. This is the case for OMe groups that have positive effects in R1 and negative effects on R2 as observed for DBA1, DBA3 and DBA5.

Concerning the biological properties, when comparing with DBA1, adding NO_2_ in R2 (DBA4) or removing the –OMe group in R1 (DBA5) did clearly increased cell viability (EC_50_ around 15 μM) while the other substitutions had detrimental effects on cell viability (EC_50_ around 3–4 μM). The addition at position R2 of a –NO_2_ had no significant effects on HIV-1 inhibition with IC_50_ around 1 μM. However, the addition of the –OMe group in the R1 position did slightly increase inhibition when comparing DBA1 and DBA5.

To assess the relationship between biophysical parameters and antiviral activity, we focused on DBA1, DBA4, and DBA5, as DBA2 and DBA3 could not be reliably analysed due to overlapping cytotoxic and antiviral effects. DBA1 and DBA4 display comparable profiles, with similar antiviral potency (IC_50_ = 0.9 and 1 μM) and strong LTR4 stabilization (Δ*T*_m_ = +11.5 °C and +11 °C). In contrast, DBA5 exhibits weaker G4 stabilization (Δ*T*_m_ = +5 °C) and reduced antiviral activity (IC_50_ = 3 μM). For these three compounds, the biophysical and antiviral data correlate well within this non-cytotoxic subset, supporting a relationship between G4 interaction strength and antiviral potency. However, additional factors, such as cellular uptake or intracellular distribution, likely modulate antiviral potency.

**Fig. 9:**
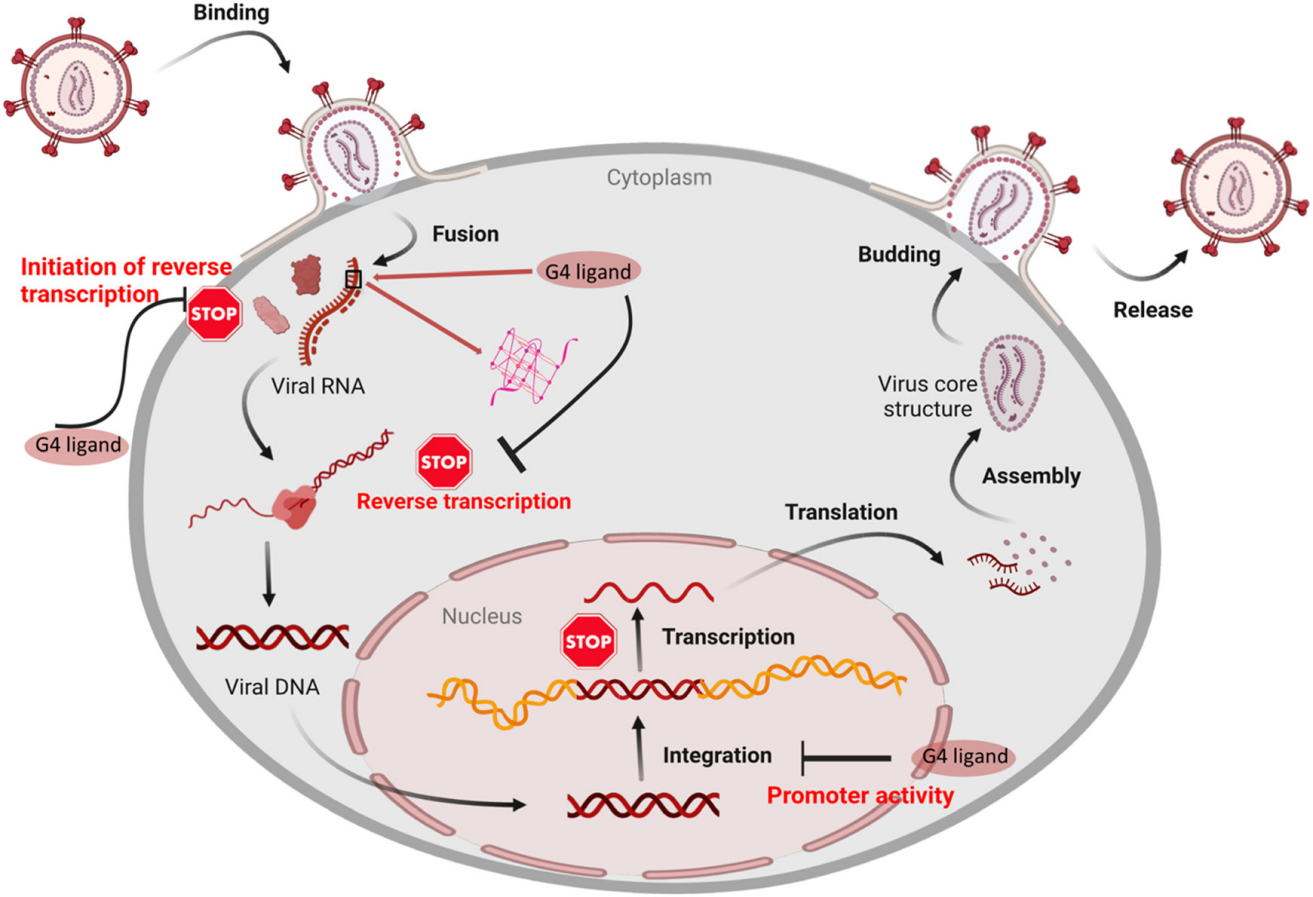
Potential HIV-1 inhibition mechanisms. Schematic representation of the HIV replication cycle and the inhibitory action of G4 ligands. The virus first binds to the host cell and fuses with its membrane. The viral RNA undergoes reverse transcription to form viral DNA and integrates into the host genome. Transcription of viral genes occurs, followed by translation and assembly of viral proteins into new virus particles. These particles bud off and are released to infect other cells. G4 ligands inhibit key stages of the HIV-1 replication cycle. They block the initiation and progression of reverse transcription, preventing viral RNA from converting into DNA. Additionally, G4 ligands suppress promoter activity within the nucleus, reducing the transcription of viral genes. By targeting these stages, G4 ligands disrupt the formation and release of new HIV particles.

Porphyrins,^26,42^ naphthalene diimides^38^ and Braco19^41^ G4 ligands have also been shown to inhibit HIV-1 replication with different inhibition mechanisms (Fig. 9). The works on naphthalene diimides and Braco19 suggested that G4 ligands acted predominantly during the reverse transcription step with a minor contribution in the post-integration stages. In a recent study, we found that the formation of HIV-1 viral G4s (from the cPPT, PPT or U3 regions) occurs during the early stages of the viral cycle. We showed that porphyrins inhibit HIV-1 by preventing the initiation of reverse transcription within the first hour post-infection.^26^ The mechanism of action of G4 ligands against HIV-1 may also depend on the chemical nature of the molecule. As a result, DBAs may share a similar mechanism involving the early stages of HIV-1 infection.

## 4. Conclusion

To our knowledge, this is the first report identifying dibenzoacridinium (DBA)-based compounds as selective G4 ligands. While a few structurally related scaffolds have been previously studied—such as BRACO-19^102^ a known G4 ligand with an acridine core—it lacks the permanent central positive charge that characterizes the DBA core. The closest structural analog, an acridinium betain derivative,^103^ also bears a permanent positive charge on the nitrogen atom, but lacks the additional phenyl substitution and side-chain diversity found in our DBA ligands. Other derivatives such as dibenzo-naphtho-thebenidinium (DBNT)^104^ share a similar extended aromatic core and cationic center, but have been studied for their self-assembling properties in aqueous solutions, not for G4 recognition. Neither acridinium betain nor DBNT derivatives have been proposed or validated as G4-binding agents. RHPS4 is also closely related to DBA derivatives. It is a rigid planar tetracyclic indoloquinoline that binds G4s with high affinity but suffers from well-documented poor selectivity with strong off-target interactions notably with duplex DNA. Our results demonstrate that DBA compounds bind G4 structures with high affinity (*K*_d_ as low as 0.14 μM determined by ESI-MS) and induce strong thermal stabilization (Δ*T*_m_ up to +18 °C determined by FRET melting assay), as confirmed by multiple biophysical approaches. In cellular assays, the most active compounds (DBA4, 5) exhibited antiviral activity against HIV-1 with IC_50_ values ranging from 1 μM to 3 μM. Altogether, this first generation of DBAs defines a new class of G4-ligands combining a modular aromatic core and a permanent positive charge. Further structural optimization aimed at improving antiviral potency and cellular viability could pave the way toward the development of potent antiviral therapeutics.

## Author contributions

AK: investigation, formal analysis, writing original draft, review & editing. Performed chemical synthesis, biophysical characterization (CD, FRET-melting, fluorescence quenching assay), data analysis and interpretation. CB: investigation, formal analysis, writing – review & editing. Conducted native ESI-MS experiments and data analysis. AG: investigation, formal analysis. Performed FRET-melting, fluorescence quenching assay. BK: investigation, formal analysis. Performed X-ray crystallography and structure determination of DBA1. EL: Investigation, formal analysis, writing – review & editing. Conducted molecular dynamics simulations and computational analysis. JM: investigation, formal analysis. Performed NMR spectroscopy experiments and structural analysis. PB: investigation, formal analysis. Performed NMR structural analysis. ZM: investigation, formal analysis. Performed NMR spectroscopy experiments. YF: conceptualization, funding acquisition. FR: supervision and conceptualization of native ESI-MS experiments and data analysis. VG: supervision and conceptualization of native ESI-MS experiments and data analysis. MLA: conceptualization, funding acquisition, writing – review & editing. Investigation: conducted cell-based assays/antiviral activity assays. CO: conceptualization, supervision, funding acquisition, investigation, formal analysis, writing – review & editing. Investigation: performed chemical synthesis. SA: conceptualization, supervision, project administration, funding acquisition, investigation, formal analysis, writing – original draft, writing – review & editing. Designed and supervised the overall study. Investigation: NMR spectroscopy experiments and structural analysis. All authors have read and approved the final manuscript.

## Conflicts of interest

None declared.

## Supplementary Material

MD-017-D5MD01178G-s001

MD-017-D5MD01178G-s002

## Data Availability

Supplementary information (SI): with additional experimental data for synthetic routes (detailed protocols), MS spectra, CD and melting profiles (see https://doi.org/10.1039/d5md01178g). Data from docking, quantum mechanics and molecular dynamics experiments are deposited on Zenodo: https://doi.org/10.5281/zenodo.18785696. CCDC 2419961 contains the supplementary crystallographic data for this paper.^105^
