# Supplementary material for: Dibenzoacridinium derivatives: a new class of G-quadruplex ligands with anti-HIV-1 properties
Source: RSC Med Chem. 2026 Jul 6;17(7):3426–46. doi: 10.1039/d5md01178g (PMC13334477; doi:10.1039/d5md01178g)
Supplement: MD-017-D5MD01178G-s001 [file MD-017-D5MD01178G-s001.pdf]

Supplementary Information (SI) for RSC Medicinal Chemistry.
This journal is © The Royal Society of Chemistry 2026

Supplementary Information

Dibenzoacridinium derivatives : A new class of G-Quadruplex ligands with anti-HIV-1 properties

Amani Kabbara,[1] Corinne Buré,[2] Aurore Guédin[1], Brice Kauffmann,[2] Eric Largy,[1] Julien Marquevielle[1], Pierre Bonnafous[1], Zidane Mdarhri[1,&], Yann Ferrand,[3] Valérie Gabelica[1,§], Frédéric Rosu[2,§], Marie-Line Andreola[4] , Céline Olivier,[*,5] and Samir Amrane[*,1]

[1] ARNA Laboratory, INSERM U1212, CNRS UMR 5320, IECB, Université de Bordeaux, France.

[2] Institut Européen de Chimie et Biologie, Univ. Bordeaux, CNRS, INSERM, IECB, US1, UAR 3033, F-33600 Pessac, France.

[3] Laboratoire de Chimie et Biologie des Membranes et des Nano-objets, UMR 5248 CNRS, Université de Bordeaux, 2 rue Robert Escarpit, F-33600 Pessac, France.

[4] Laboratoire de Microbiologie Fondamentale et Pathogénicité, UMR 5234 CNRS, Université de Bordeaux, 146 Rue Léo Saignat, F-33076 Bordeaux, France.

[5] Institut des Sciences Moléculaires, UMR 5255 CNRS, Université de Bordeaux, 351 Cours de la Libération, F-33405 Talence Cedex, France.

[§]Present address : School of Pharmaceutical Sciences, University of Geneva, CMU, 1-Rue Michel Servet, 1211 Geneva 4, Switzerland.

[&] Present address : Department of Chemistry, University of Liverpool, Liverpool L69 7ZD, U.K.

[*]Corresponding authors

Supplementary Materiel and Methods

Synthetic routes for DBA1-5 synthesis

1 Materials and methods

All reagents were obtained from commercially available sources and used without further purification. Solvents were dried from appropriate drying agents (sodium for toluene; calcium hydride for dichloromethane) and freshly distilled before use. Dimethylformamide was purified through azeotropic distillation with water and benzene.

^{1}H NMR and ^{13}C NMR analyses were performed on Bruker Avance 300 and DPX 400 spectrometers. Chemical shift values are given in ppm with reference to solvent residual signals. HR-MS analyses were performed on a Qstar spectrometer. **All compounds are >95% pure by NMR analysis**

2 Synthetic routes

General procedure A (Buchwald-Hartwig cross-coupling reaction). In dry and degassed toluene were introduced $Pd(OAc)_2$ (0.06 equiv.) and P(*t*-Bu)$_3$ (0.12 equiv.). After 15 min of stirring, aniline derivative (1 equiv.), bromonaphthalene derivative (4 equiv.) and Cs_2CO_3 (3 equiv.) were added successively. The solution was refluxed three days, cooled down to RT and diluted with CH_2Cl_2. The crude mixture was filtered, evaporated to dryness and purified on silica gel column (cyclohexane/CH_2Cl_2 8:2, v/v) to afford the target compounds as coloured powders.

Synthesis of 1. General procedure **A** was applied using toluene (100 mL), $Pd(OAc)_2$ (148 mg, 0.66 mmol, 6%), P(*t*-Bu)$_3$ (0.32 mL, 1.3 mmol, 12%), aniline (1 mL, 11 mmol, 1 equiv.), 2-bromo-6-methoxynaphthalene (10.4 g, 44 mmol, 4 equiv.) and Cs_2CO_3 (10.7 g, 33 mmol, 3 equiv.) to afford **1** as a pale yellow powder (2.7 g, 6.6 mmol, 60 % yield). ^{1}H NMR (300 MHz, $CDCl_3$): δ 7.76 (d, 2H, $^{3}J_{H-H}$= 9.0 Hz), 7.63 (d, 2H, $^{3}J_{H-H}$= 9.0 Hz), 7.41 (s, 2H), 7.28-7.20 (m, 6H), 7.09-7.01 (m, 5H), 3.84 (s, 6H). ^{13}C NMR (75 MHz, $CDCl_3$): δ 157.6, 132.9, 129.8, 129.6, 129.3, 129.1, 127.4, 126.6, 126.3, 123.4, 119.6, 118.4, 116.2, 106.0, 55.2. HR-MS ESI+ (m/z): 405.1729 $[M+H]^+$ (calcd. 405.1729 for $[C_{28}H_{23}NO_2]^+$).

Synthesis of 2. General procedure **A** was applied using toluene (100 mL), $Pd(OAc)_2$ (164 mg, 0.7 mmol, 6%), P(*t*-Bu)$_3$ (0.35 mL, 1.5 mmol, 12%), *p*-anisidine (1.5 g, 12.2 mmol, 1 equiv.), 2-bromo-6-methoxynaphthalene (11.5 g, 48.8 mmol, 4 equiv.) and Cs_2CO_3 (11.9 g, 36.6 mmol, 3 equiv.) to afford **3** as a pale yellow powder (3.1 g, 7.1 mmol, 58 % yield). ^{1}H NMR (300 MHz, $CDCl_3$):
δ 7.72 (d, 2H, $^{3}J_{H-H}$= 9.0 Hz), 7.58 (d, 2H, $^{3}J_{H-H}$= 9.0 Hz), 7.29 (d, 2H, $^{4}J_{H-H}$= 2.3 Hz), 7.24 (d, 2H, $^{4}J_{H-H}$= 2.3 Hz), 7.18 (dd, 2H, $^{3}J_{H-H}$= 8.8 Hz, $^{4}J_{H-H}$= 2.2 Hz), 7.06 (d, 2H, $^{3}J_{H-H}$= 8.9 Hz), 7.05 (d, 2H, $^{3}J_{H-H}$= 9.0 Hz), 6.93 (d, 2H, $^{3}J_{H-H}$= 9.0 Hz), 3.83 (s, 6H), 3.75 (s, 3H). ^{13}C NMR (75 MHz, $CDCl_3$): δ 156.8, 156.0, 144.3, 141.3, 130.8, 129.9, 128.4, 127.7, 126.7,

124.7, 119.6, 118.9, 114.8, 106.0, 55.6, 55.4. HR-MS ESI+ (m/z): 435.1831 $[M+H]^+$ (calcd. 435.1834 for $[C_{29}H_{25}NO_3]^+$).

Synthesis of 3. General procedure **A** was applied using toluene (100 mL), $Pd(OAc)_2$ (135 mg, 0.6 mmol, 6%), P(*t*-Bu)$_3$ (0.3 mL, 1.2 mmol, 12%), methyl 4-aminobenzoate (1.5 g, 10 mmol, 1 equiv.), 2-bromo-6-methoxynaphthalene (9.5 g, 40 mmol, 4 equiv.) and Cs_2CO_3 (9.7 g, 30 mmol, 3 equiv.) to afford **2** as an orange powder (2.8 g, 6.1 mmol, 61 % yield). ^{1}H NMR (300 MHz, $CDCl_3$): δ 7.83 (d, 2H, $^3J_{H\text{-}H}$= 9.1 Hz), 7.80 (d, 2H, $^3J_{H\text{-}H}$= 9.0 Hz), 7.71 (d, 2H, $^3J_{H\text{-}H}$= 9.1 Hz), 7.62 (d, 2H, $^4J_{H\text{-}H}$= 2.2 Hz), 7.32-7.28 (m, 4H), 7.12 (dd, 2H, $^3J_{H\text{-}H}$= 9.0 Hz, $^4J_{H\text{-}H}$= 2.5 Hz), 6.93 (d, 2H, $^3J_{H\text{-}H}$= 9.0 Hz), 3.86 (s, 6H), 3.78 (s, 3H). ^{13}C NMR (75 MHz, $CDCl_3$): δ 165.9, 157.1, 151.9, 141.5, 131.9, 130.8, 129.3, 128.8, 128.5, 125.7, 123.4, 120.7, 119.0, 118.5, 106.0, 55.2, 51.6. HR-MS ESI+ (m/z): 486.1655 $[M+Na]^+$ (calcd. 486.1676 for $[C_{30}H_{25}NO_4Na]^+$).

Synthesis of 4. General procedure **A** was applied using toluene (100 mL), $Pd(OAc)_2$ (135 mg, 0.6 mmol, 6%), P(*t*-Bu)$_3$ (0.3 mL, 1.2 mmol, 12%), 4-nitroaniline (1.4 g, 10 mmol, 1 equiv.), 2-bromo-6-methoxynaphthalene (9.5 g, 40 mmol, 4 equiv.) and Cs_2CO_3 (9.7 g, 30 mmol, 3 equiv.) to afford **4** as a red powder (3.6 g, 8.0 mmol, 80 % yield). ^{1}H NMR (300 MHz, $CDCl_3$): δ 8.07 (d, 2H, $^3J_{H\text{-}H}$= 9.4Hz), 7.90 (d, 2H, $^3J_{H\text{-}H}$= 8.9 Hz), 7.78 (d, 2H, $^3J_{H\text{-}H}$= 8.9 Hz), 7.77 (d, 2H, $^4J_{H\text{-}H}$= 2.2 Hz), 7.38 (dd, 2H, $^3J_{H\text{-}H}$= 8.9 Hz, $^4J_{H\text{-}H}$= 2.3 Hz), 7.36 (d, 2H, $^4J_{H\text{-}H}$= 2.6 Hz), 7.16 (dd, 2H, $^3J_{H\text{-}H}$= 9.0 Hz, $^4J_{H\text{-}H}$= 2.5 Hz), 6.89 (d, 2H, $^3J_{H\text{-}H}$= 9.4 Hz), 3.87 (s, 6H). ^{13}C NMR (75 MHz, $CDCl_3$): δ 157.5, 153.6, 140.5, 138.9, 132.5, 129.2, 129.0, 128.8, 125.9, 125.7, 124.5, 119.2, 116.9, 106.0, 55.2. HR-MS ESI+ (m/z): 473.1485 $[M+Na]^+$ (calcd. 473.1472 for $[C_{28}H_{22}N_2O_4Na]^+$).

Synthesis of 5. General procedure **A** was applied using toluene (100 mL), $Pd(OAc)_2$ (135 mg, 0.6 mmol, 6%), P(*t*-Bu)$_3$ (0.3 mL, 1.2 mmol, 12%), aniline (0.93 g, 10 mmol, 1 equiv.), 2-bromo-naphthalene (8.3 g, 40 mmol, 4 equiv.) and Cs_2CO_3 (9.7 g, 30 mmol, 3 equiv.) to afford **5** as a pale yellow powder (3 g, 8.7 mmol, 87 % yield). ^{1}H NMR (300 MHz, $CDCl_3$): δ 7.80 (d, 2H, $^3J_{H\text{-}H}$= 7.1 Hz), 7.77 (d, 2H, $^3J_{H\text{-}H}$= 9.1 Hz), 7.61 (d, 2H, $^3J_{H\text{-}H}$= 7.1 Hz), 7.50 (d, 2H, $^4J_{H\text{-}H}$= 2.2 Hz), 7.46-7.29 (m, 8H), 7.21 (d, 2H, $^3J_{H\text{-}H}$= 8.7 Hz), 7.11 (t, 1H, $^3J_{H\text{-}H}$= 7.2 Hz). ^{13}C NMR (75 MHz, $CDCl_3$): δ 147.8, 145.5, 134.5, 130.3, 129.4, 129.0, 127.7, 127.1, 126.4, 124.8, 124.7, 124.6, 123.2, 120.6. HR-MS ESI+ (m/z): 345.1514 $[M+H]^+$ (calcd. 345.1517 for $[C_{26}H_{19}N]^+$).

General procedure B (synthesis of dibenzoacridinium compounds). In a general procedure, arylamine precursor (1 eq.) was solubilized in dry DMF and the mixture was cooled down to 0°C. Phosphorus oxychloride (2 eq.) was added dropwise under continuous stirring. The reaction mixture was allowed to warm up to room temperature and further heated up to 90°C and stirred for 3 H. After removal of the solvent under vacuum, the crude product was dissolved in a mixture of dichloromethane and methanol (8:1). The target compound precipitated as a highly coloured solid by addition of ethyl acetate. The crude product was purified on silica gel column (CH_2Cl_2/MeOH (9:1, v/v)) to afford dibenzoacridinium compounds deeply coloured powders.

Synthesis of DBA1. General procedure **B** was applied using precursor **1** (2 g, 5 mmol, 1 eq.), dry DMF (30 mL) and $POCl_3$ (0.9 mL, 10 mmol, 2 eq.) to afford **DBA1** as a yellow powder (1.8 g, 4.1 mmol, 82 % yield). ^1H NMR (300 MHz, dmso-d6): δ 11.22 (s, 1H), 9.66 (d, 2H, $^3J_{H-H}$= 9.4 Hz), 8.50 (d, 2H, $^3J_{H-H}$= 9.8 Hz), 7.98-7.96 (m, 3H), 7.88-7.85 (m, 2H), 7.77 (d, 2H, $^4J_{H-H}$= 2.6 Hz), 7.70 (dd, 2H, $^3J_{H-H}$= 9.4 Hz, $^4J_{H-H}$= 2.6 Hz), 7.32 (d, 2H, $^3J_{H-H}$= 9.8 Hz), 3.99 (s, 6H). ^{13}C NMR (75 MHz, dmso-d6): δ 160.1, 139.9, 139.2, 137.8, 135.4, 132.0, 131.6, 131.3, 127.8, 126.7, 125.0, 122.1, 120.2, 117.5, 115.0, 110.1, 55.7. HR-MS ESI+ (m/z): 416.1503 $[M]^+$ (calcd. 416.1645 for $[C_{29}H_{22}NO_2]^+$).

Synthesis of DBA2. General procedure **B** was applied using precursor of **2** (2.3 g, 5 mmol, 1 eq.), DMF (30 mL) and $POCl_3$ (0.9 mL, 10 mmol, 2 eq.) to afford **DBA3** as a yellow powder (1.7 g, 3.4 mmol, 68 % yield). ^1H NMR (300 MHz, dmso-d6): δ 11.23 (s, 1H), 9.67 (d, 2H, $^3J_{H-H}$= 9.3 Hz), 8.51 (d, 2H, $^3J_{H-H}$= 8.6 Hz), 8.48 (d, 2H, $^3J_{H-H}$= 9.5 Hz), 8.04 (d, 2H, $^3J_{H-H}$= 8.6 Hz), 7.79 (d, 2H, $^4J_{H-H}$= 2.6 Hz), 7.70 (dd, 2H, $^3J_{H-H}$= 9.2 Hz, $^4J_{H-H}$= 2.6 Hz), 7.36 (d, 2H, $^3J_{H-H}$= 9.7 Hz), 4.02 (s, 3H), 3.99 (s, 6H). ^{13}C NMR (75 MHz, dmso-d6): δ 165.4, 160.2, 156.2, 141.5, 139.8, 139.4, 132.5, 132.2, 132.1, 128.6, 126.8, 125.0, 122.1, 120.3, 117.6, 110.3, 55.8, 52.8. HR-MS ESI+ (m/z): 474.1694 $[M]^+$ (calcd. 474.1705 for $[C_{31}H_{24}NO_4]^+$).

Synthesis of DBA3. General procedure **B** was applied using precursor **3** (2.1 g, 5 mmol, 1 eq.), DMF (30 mL) and $POCl_3$ (0.9 mL, 10 mmol, 2 eq.) to afford **DBA2** as a yellow powder (1.4 g, 3.0 mmol, 61 % yield). ^1H NMR (300 MHz, dmso-d6): δ 11.12 (s, 1H), 9.61 (d, 2H, $^3J_{H-H}$= 9.3 Hz), 8.47 (d, 2H, $^3J_{H-H}$= 9.7 Hz), 7.78 (d, 2H, $^3J_{H-H}$= 9.0 Hz), 7.73 (d, 2H, $^4J_{H-H}$= 2.7 Hz), 7.64 (dd, 2H, $^3J_{H-H}$= 9.2 Hz, $^4J_{H-H}$= 2.6 Hz), 7.49 (d, 2H, $^3J_{H-H}$= 9.0 Hz), 7.38 (d, 2H, $^3J_{H-H}$= 9.7 Hz), 4.00 (s, 3H), 3.97 (s, 6H). ^{13}C NMR (75 MHz, dmso-d6): δ 161.0, 160.1, 140.4, 139.1, 135.3, 132.0, 130.3, 129.1, 126.8, 125.0, 122.1, 120.2, 117.7, 116.3, 110.1, 55.9, 55.8. HR-MS ESI+ (m/z): 446.1761 $[M]^+$ (calcd. 446.1751 for $[C_{30}H_{24}NO_3]^+$).

Synthesis of DBA4. General procedure **B** was applied using precursor **4** (2.2 g, 5 mmol, 1 eq.), DMF (30 mL) and $POCl_3$ (0.9 mL, 10 mmol, 2 eq.) to afford **DBA4** as an orange powder (1.9 g, 3.9 mmol, 78 % yield). ^1H NMR (300 MHz, dmso-d6): δ 11.33 (s, 1H), 9.71 (d, 2H, $^3J_{H-H}$= 9.3 Hz), 8.83 (d, 2H, $^3J_{H-H}$= 9.0 Hz), 8.52 (d, 2H, $^3J_{H-H}$= 9.6 Hz), 8.19 (d, 2H, $^3J_{H-H}$= 9.0 Hz), 7.86 (d, 2H, $^4J_{H-H}$= 2.6 Hz), 7.76 (dd, 2H, $^3J_{H-H}$= 9.2 Hz, $^4J_{H-H}$= 2.6 Hz), 7.44 (d, 2H, $^3J_{H-H}$= 9.6 Hz), 4.02 (s, 6H). ^{13}C NMR (75 MHz, dmso-d6): δ 160.3, 149.3, 142.8, 139.8, 139.5, 136.1, 132.1, 129.9, 126.8, 125.1, 122.1, 120.5, 117.6, 110.3, 55.8. HR-MS ESI+ (m/z): 461.1496 $[M]^+$ (calcd. 461.1496 for $[C_{29}H_{21}N_2O_4]^+$).

Synthesis of DBA5. General procedure **B** was applied using precursor **5** (1.38 g, 4 mmol, 1 eq.), dry DMF (20 mL) and $POCl_3$ (0.73 mL, 8 mmol, 2 eq.) to afford **DBA5** as a yellow powder (1.4 g, 3.6 mmol, 92 % yield). ^1H NMR (300 MHz, dmso-d6): δ 11.55 (s, 1H), 9.85 (d, 2H, $^3J_{H-H}$= 8.3 Hz), 8.64 (d, 2H, $^3J_{H-H}$= 9.6 Hz), 8.34 (d, 2H, $^3J_{H-H}$= 7.3 Hz), 8.19 (t, 2H, $^3J_{H-H}$= 7.2 Hz), 8.06-7.97 (m, 5H), 7.89 (m, 2H), 7.37 (d, 2H, $^3J_{H-H}$= 9.6 Hz). ^{13}C NMR (75 MHz, dmso- d6): δ 141.6, 140.2, 137.8, 137.3, 131.7, 131.3, 130.5, 130.1, 129.9, 129.8, 128.3, 127.8, 125.1, 124.8, 117.11. HR-MS ESI+ (m/z): 356.1451 $[M]^+$ (calcd. 356.1439 for $[C_{27}H_{18}N]^+$).

Supplementary Figures and tables

Table of content

Table S1: Molecular weights and molar extinction coefficients (ε) for DBA compounds

Table S2: FRET melting assay

Table S3: Fluorescence Quenching assay

Figure S1: UV absorbance and Circular Dichroism spectra recorded in parallel

Figure S2: Circular Dichroism (CD) spectra of five distinct G4 conformations

Figure S3: CD spectra of various oligonucleotides titrated with DBA1.

Figure S4: CD spectra of various oligonucleotides titrated with DBA2.

Figure S5: CD spectra of various oligonucleotides titrated with DBA4.

Figure S6: CD spectra of various oligonucleotides titrated with DBA5.

Figure S7: FRET melting assay principle

Figure S8: Principle of the Fluorescence Quenching Assay (FQA).

Figure S9: Native electrospray mass spectra of complexes of rLTR4, Htel and TG4T

Figure S10: Native electrospray mass spectra of complexes of G4T4G4, DK66 and DK100

Figure S11: Radar plot representation of ESI-MS results

Figure S22: Molecular Docking and Molecular Dynamics

Table S4: Molecular formulae and corresponding SMILES formulae of the DBA compounds

Figure S12: ^{1}H NMR spectrum of **DBA1** (dmso-d6)

Figure S13: ^{13}C NMR spectrum of **DBA1** (dmso-d6)

Figure S14: ^{1}H NMR spectrum of **DBA2** (dmso-d6)

Figure S15: ^{13}C NMR spectrum of **DBA2** (dmso-d6)

Figure S16: ^{1}H NMR spectrum of **DBA3**(dmso-d6)

Figure S17: ^{13}C NMR spectrum of **DBA3** (dmso-d6)

Figure S18: ^{1}H NMR spectrum of **DBA4** (dmso-d6)

Figure S19: ^{13}C NMR spectrum of **DBA4** (dmso-d6)

Figure S20: ^{1}H NMR spectrum of **DBA5**(dmso-d6)

Figure S21: ^{13}C NMR spectrum of **DBA5** (dmso-d6)

Table S1: Molecular weights and molar extinction coefficients (ε) for dibenzoacridinium compounds

Name	M.W (g.mol^{-1})	Wavelengths	ε (M^{-1}.cm^{-1})
**DBA1**	416.49945	320 nm	44635
		450 nm	16792
**DBA2**	474.53545	320 nm	45672
		450 nm	17293
**DBA3**	446.52545	320 nm	49078
		450 nm	18109
**DBA4**	461.49645	320 nm	57672
		450 nm	24396
**DBA5**	356.44745	300 nm	48252
		415 nm	19222
		440 nm	25929

Table S2: FRET melting assay

	$\Delta T_{1/2}$ (°C) LTR4		$\Delta T_{1/2}$(°C) HIVpro2		$\Delta T_{1/2}$(°C) HIVpro1		$\Delta T_{1/2}$(°C) Htel		
	No ds26	+10µM ds26	No ds26	+10µM ds26	No ds26	+10µM ds26	No ds26	+10µM ds26	Spec Index [a]
**DBA1**	11±0.2	10±0.5	4.2±1.4	3.3±0.4	6.9±0.04	4.4±0.7	17±0.2	13±0.5	0.8
**DBA2**	9.4±0.3	7.3±0.4	2.1±0.2	2.0±0.4	5.6±0.3	3.9±0.2	15±0.1	12±0.6	0.8
**DBA3**	10.6±0.7	10.2±0.3	3.5±0.2	3.8±0.6	6.3±0.7	5.0±0.1	18±1.7	14±0.1	0.9
**DBA4**	11±0.5	10±0.7	3.8±0.3	3.0±0.8	7.0±2.0	5.1±0.5	18±0.2	14±0.3	0.8
**DBA5**	8.5±0.1	6.0±1.3	1.9±0.1	1.7±0.9	5.0±0.03	3.6±0.04	12.2±0.1	9.0±0.1	0.8

[a] Specificity Index: Average of the four individual ratios calculated as follows ($\Delta T_{1/2}$ (+10µM ds26))/($\Delta T_{1/2}$ (No ds26)).

Table S3: Fluorescence Quenching assay

	rLTR4-3'Cy5 FQA					rLTR4-5'Cy5 FQA				
	K_d (10^{-6}.M) No ds26	K_d (10^{-6}.M) +10µM ds26	K_a (10^5M) No ds26	K_a (10^5M) +10µM ds26	Spec Index [a]	K_d (10^{-6}.M) No ds26	K_d (10^{-6}.M) +10µM ds26	K_a (10^5M) No ds26	K_a (10^5M) +10µM ds26	Spec Index [a]
DBA-1	3.2±0.5	3.3±0.4	3.1±0.2	2.8±0.23	0.9	5.5±1.5	8.9±1.8	1.8±0.07	1.1±0.06	0.6
DBA-2	6.7±1.5	8.6±1.3	1.5±0.06	1.2±0.08	0.8	9.3±2.9	11.6±3.8	1.1±0.03	0.9±0.03	0.8
DBA-3	6.2±1.0	6.9±1.1	1.6±0.11	1.5±0.09	0.9	8.7±1.6	11.1±1.4	1.2±0.06	0.9±0.07	0.75
DBA-4	3.3±0.4	5.3±1.1	3.0±0.24	1.9±0.1	0.6	4.4±1.0	8.4±2.1	2.3±0.1	1.2±0.05	0.5
DBA-5	4.0±0.3	4.0±0.6	2.5±0.31	2.5±0.2	1.0	6.2±1.4	9.5±2.6	1.6±0.07	1.1±0.04	0.6

[a] Specificity Index: (K_a (+10µM ds26))/(K_a (No ds26))

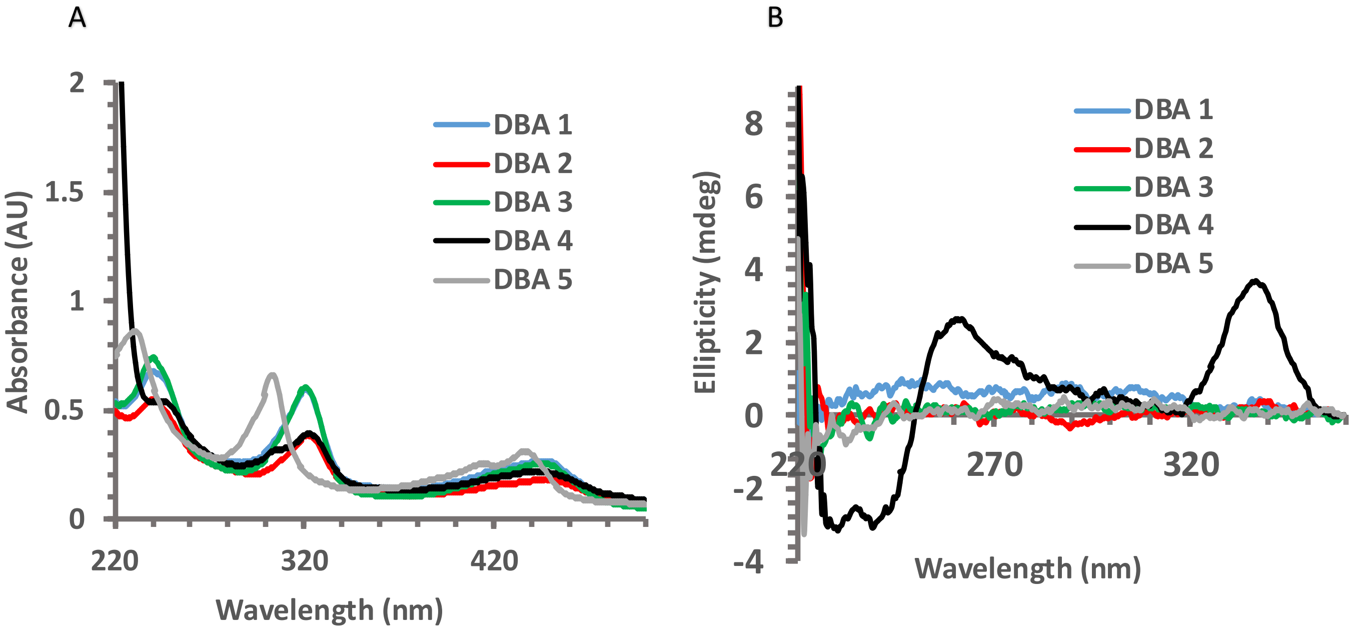

Figure S1: UV absorbance and Circular Dichroism spectra recorded in parallel. (A) UV-Visible spectra of the five DBAs (DBA1-DBA5) recorded at a concentration of 1 µM. (B) CD spectra of DBA1-DBA5, illustrating the chiroptical properties of each compound.

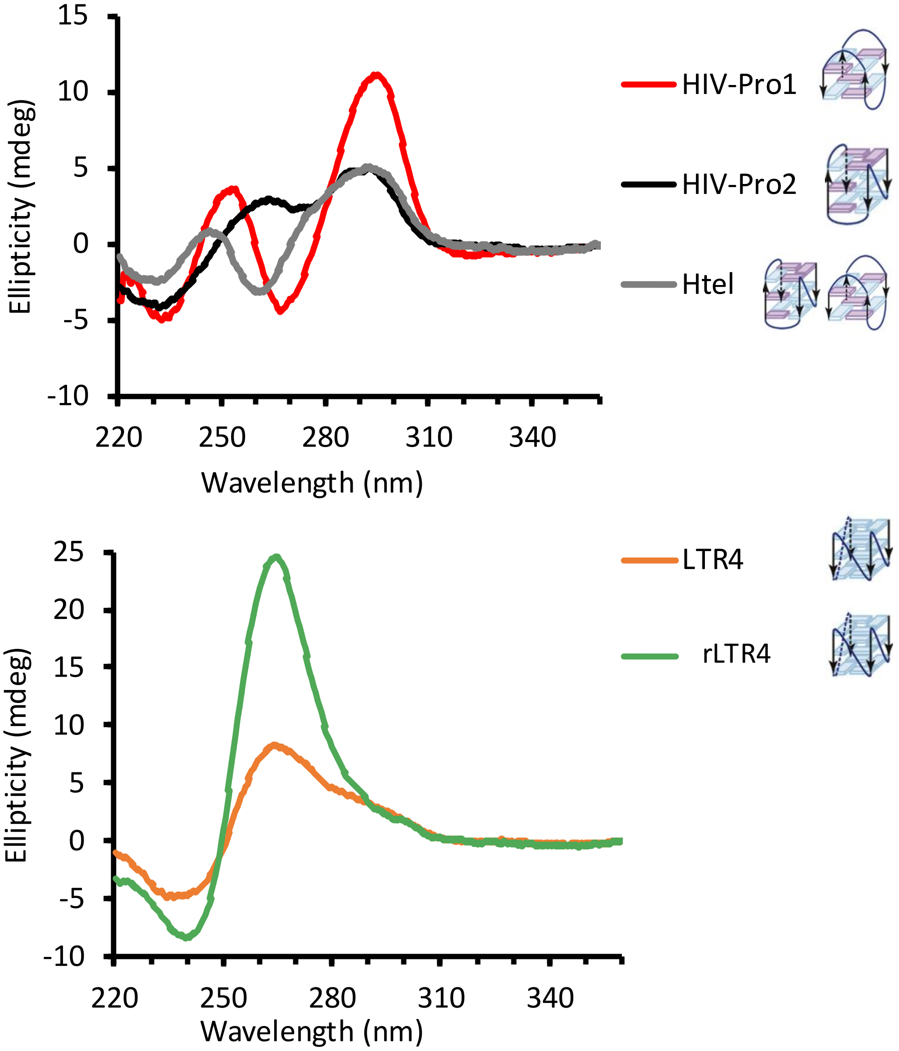

Figure S2: Circular Dichroism (CD) spectra of five distinct G4 conformations. Measurements were recorded at strand concentrations of 2.5 µM in 10 mM potassium cacodylate buffer (pH 7.0) and 100 mM KCl. HIVpro1 forms a two tetrads antiparallel G4 with its characteristic maxima at 290 nm and 250 nm and minima at 268 nm and 230 nm. HIVpro2 forms a three tetrads hybrid G4 with its characteristic maxima at 290 nm and 265 nm and minimum at 230 nm. Htel forms a mixture of antiparallel and hybrid G4s. LTR4 and rLTR4 form parallel G4s with characteristic maxima at 260 nm and minima at 240 nm.

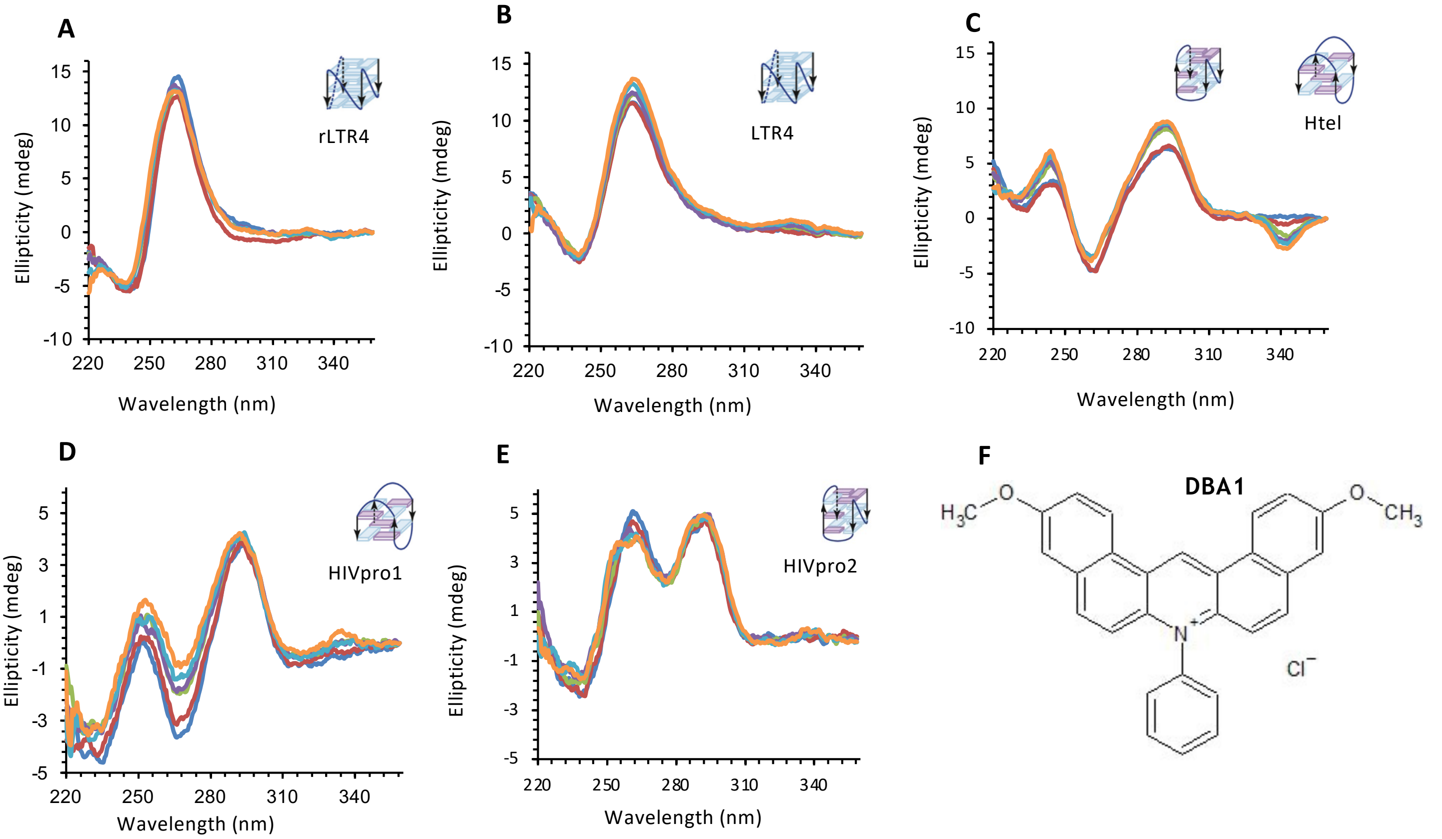

Figure S3: : **A-E)** Titration of 5 different G4 topologies (rLTR4, LTR4, Htel, HIVpro1, HIVpro2) with DBA1. Oligonucleotides were prepared at 2.5 µM in 10 mM potassium Cacodylate buffer pH 7.0 and 100 mM KCl (Dark Blue). DBA1 was added at 0.5 (red), 1 (green), 2 (purple), 3 (light blue) and 4 (orange) equivalents. The incubation time ranges between 1-5 minutes depending on starting time of spectra acquisition. The arrows highlight the change in intensity or wavelength of the peaks upon ligand addition. **F)** Chemical structure of DBA1

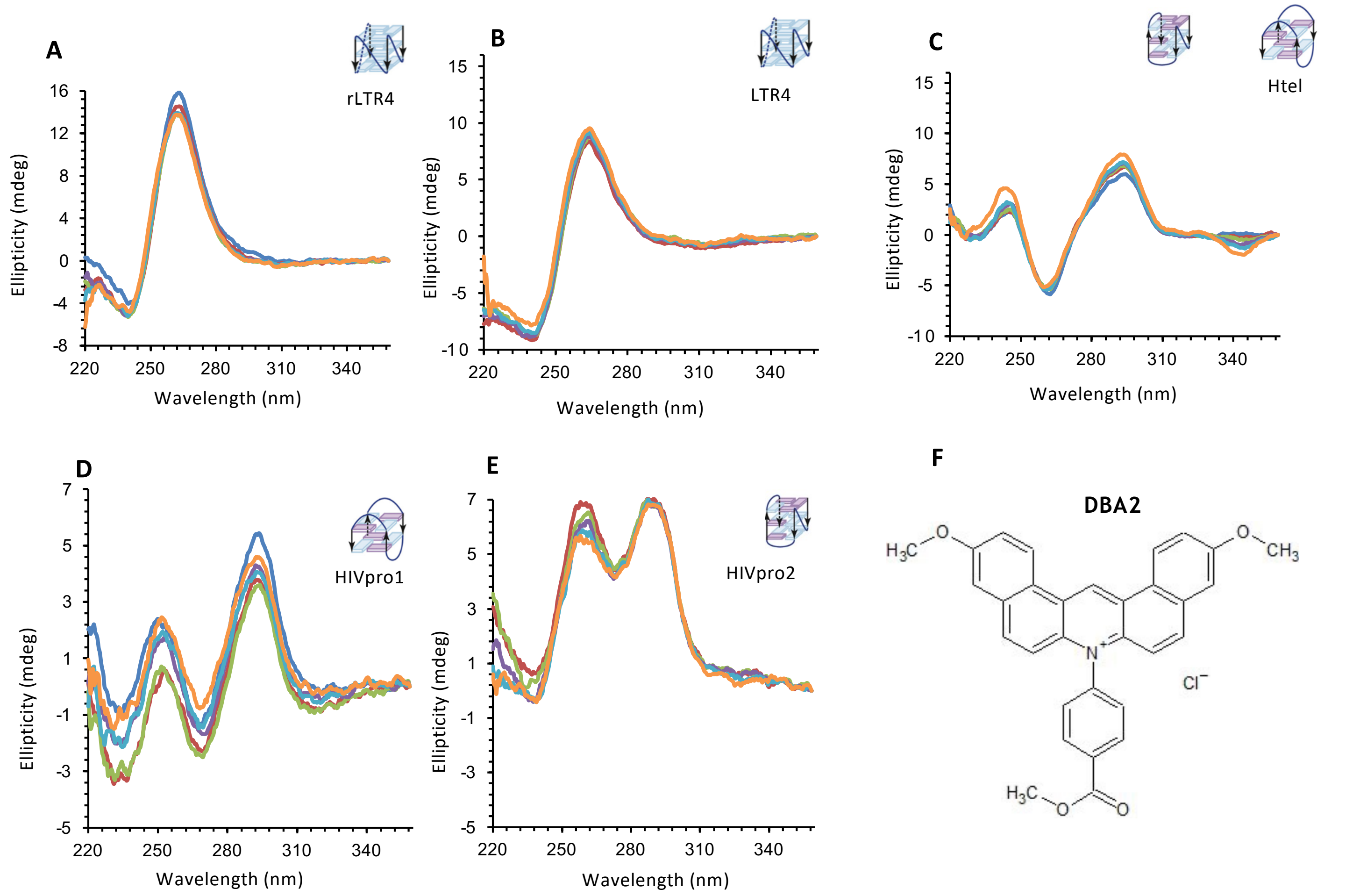

Figure S4: : **A-E)** Titration of 5 different G4 topologies (rLTR4, LTR4, Htel, HIVpro1, HIVpro2) with DBA2. Oligonucleotides were prepared at 2.5 µM in 10 mM potassium Cacodylate buffer pH 7.0 and 100 mM KCl (Dark Blue). DBA2 was added at 0.5 (red), 1 (green), 2 (purple), 3 (light blue) and 4 (orange) equivalents. The incubation time ranges between 1-5 minutes depending on starting time of spectra acquisition. The arrows highlight the change in intensity or wavelength of the peaks upon ligand addition. **F)** Chemical structure of DBA2

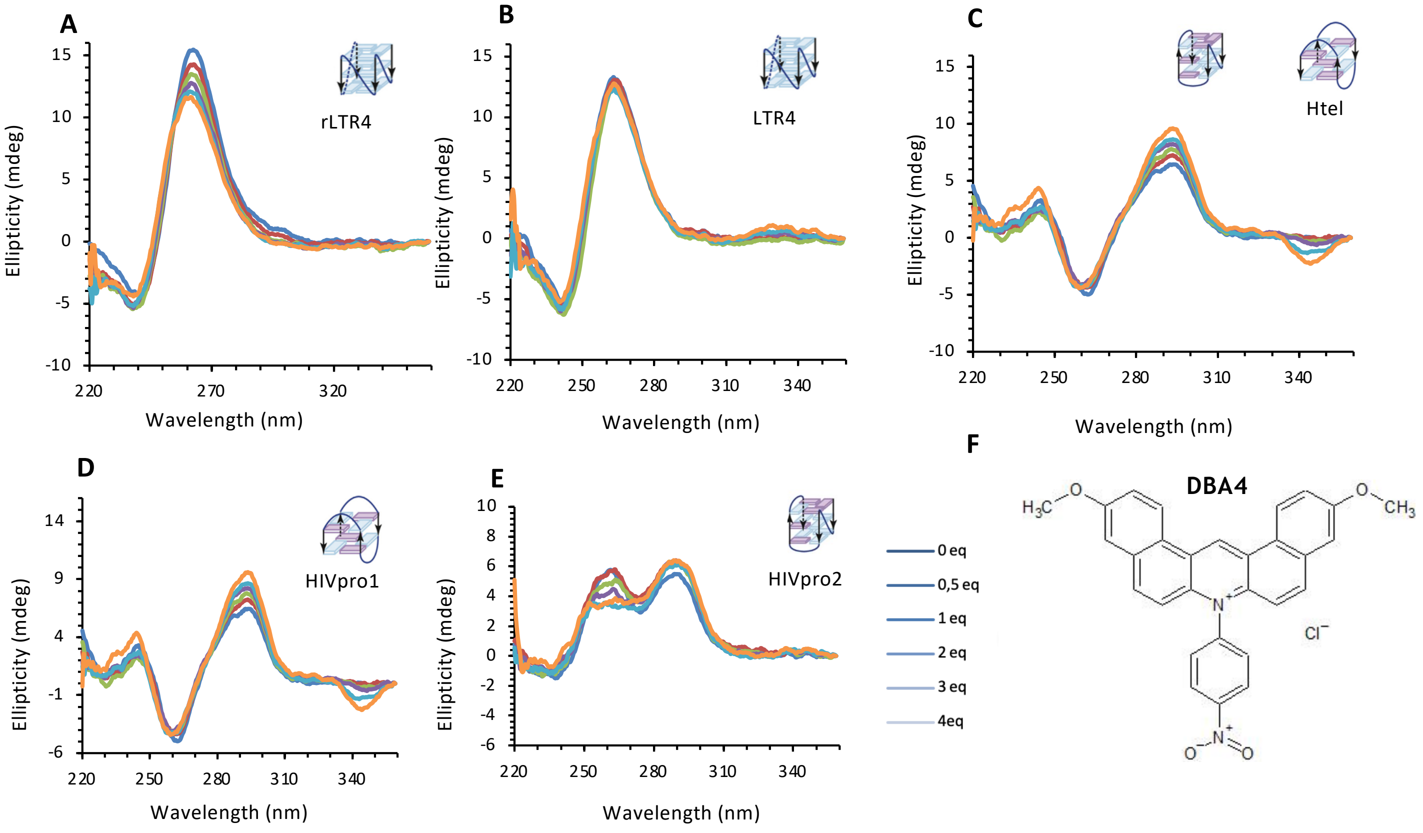

Figure S5: **: A-E)** Titration of 5 different G4 topologies (rLTR4, LTR4, Htel, HIVpro1, HIVpro2) with DBA4. Oligonucleotides were prepared at 2.5 µM in 10 mM potassium Cacodylate buffer pH 7.0 and 100 mM KCl (Dark Blue). DBA4 was added at 0.5 (red), 1 (green), 2 (purple), 3 (light blue) and 4 (orange) equivalents. The incubation time ranges between 1-5 minutes depending on starting time of spectra acquisition. The arrows highlight the change in intensity or wavelength of the peaks upon ligand addition. **F)** Chemical structure of DBA4

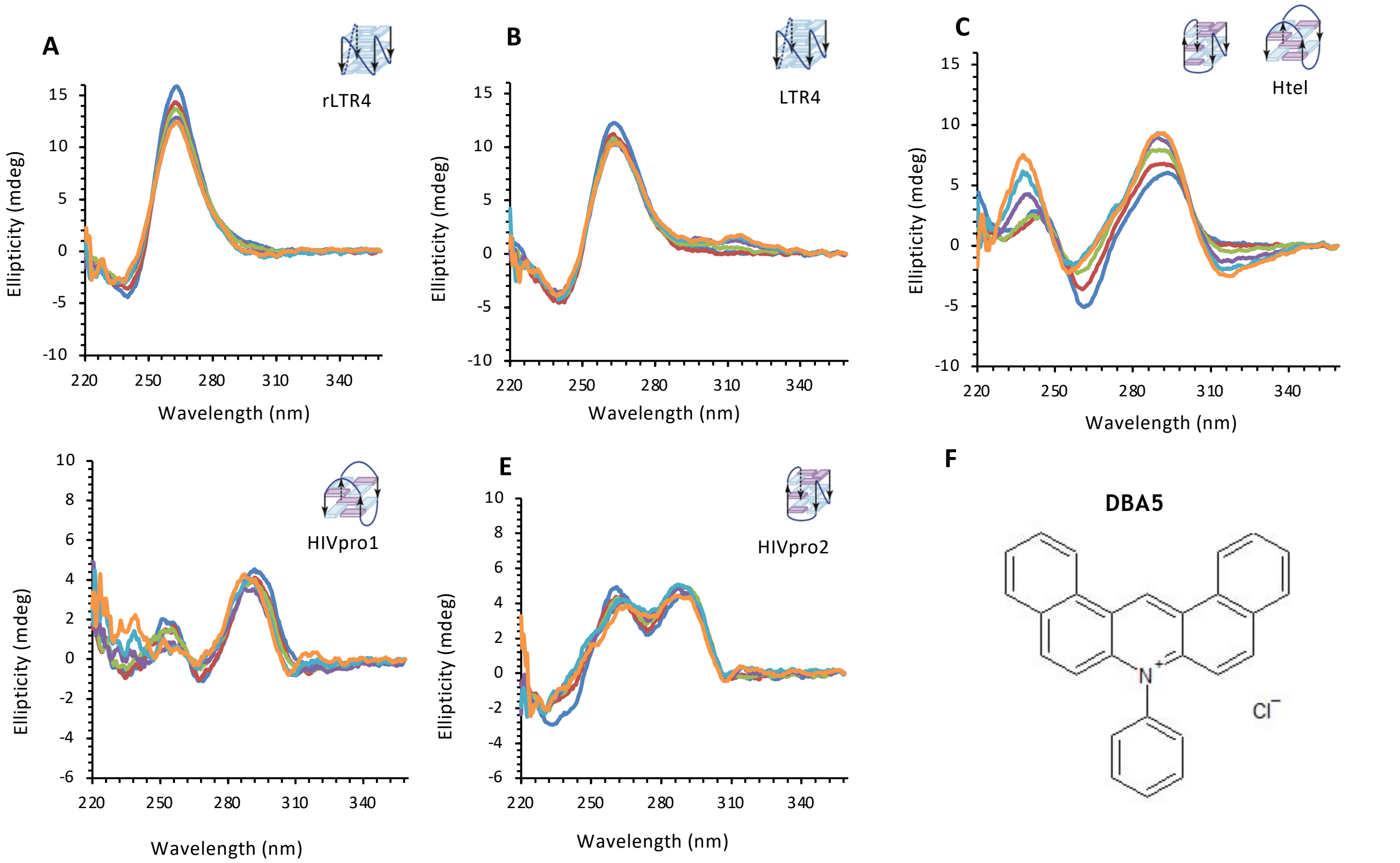

Figure S6: **: A-E)** Titration of 5 different G4 topologies (rLTR4, LTR4, Htel, HIVpro1, HIVpro2) with DBA5. Oligonucleotides were prepared at 2.5 µM in 10 mM potassium Cacodylate buffer pH 7.0 and 100 mM KCl (Dark Blue). DBA5 was added at 0.5 (red), 1 (green), 2 (purple), 3 (light blue) and 4 (orange) equivalents. The incubation time ranges between 1-5 minutes depending on starting time of spectra acquisition. The arrows highlight the change in intensity or wavelength of the peaks upon ligand addition. **F)** Chemical structure of DBA5.

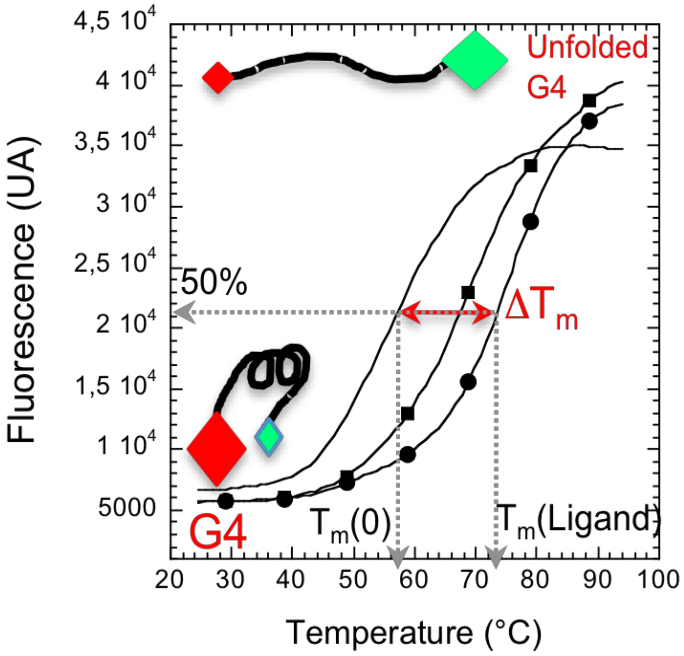

Figure S7: FRET melting assay principle.

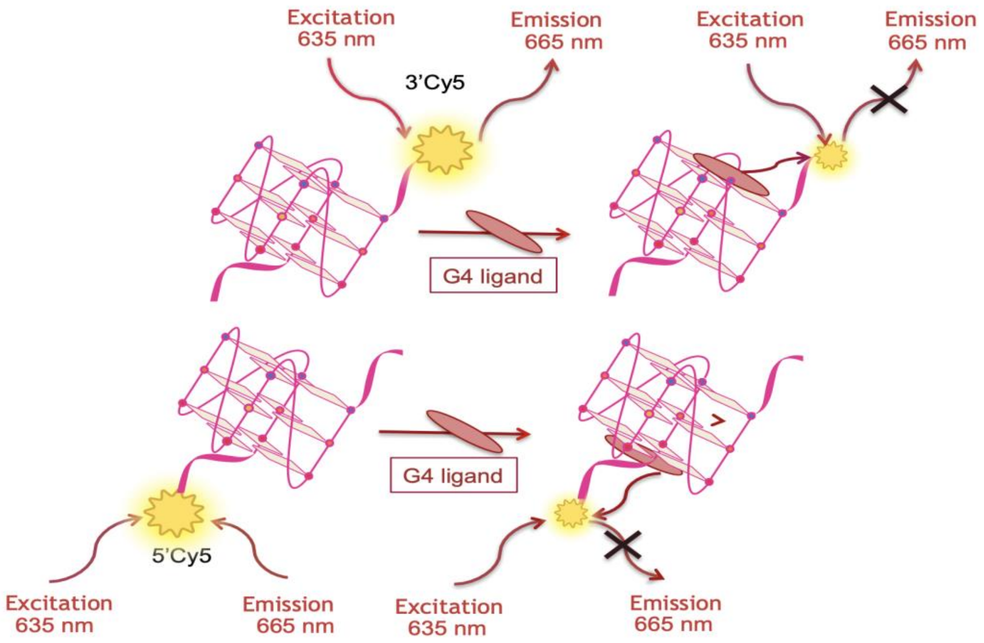

Figure S8: Principle of the Fluorescence Quenching Assay (FQA). Fluorescently labeled RNA with Cy5 at either 3' or 5' extremit**y.** The fluorescently labeled oligonucleotides were dissolved at a concentration of 10 nM in 10 mM lithium cacodylate buffer (pH 7.2) with 10 mM KCl and 90 mM LiCl. In this exemple the G4 Ligand is added at a final concentration ranging from 0 to 10 µM.

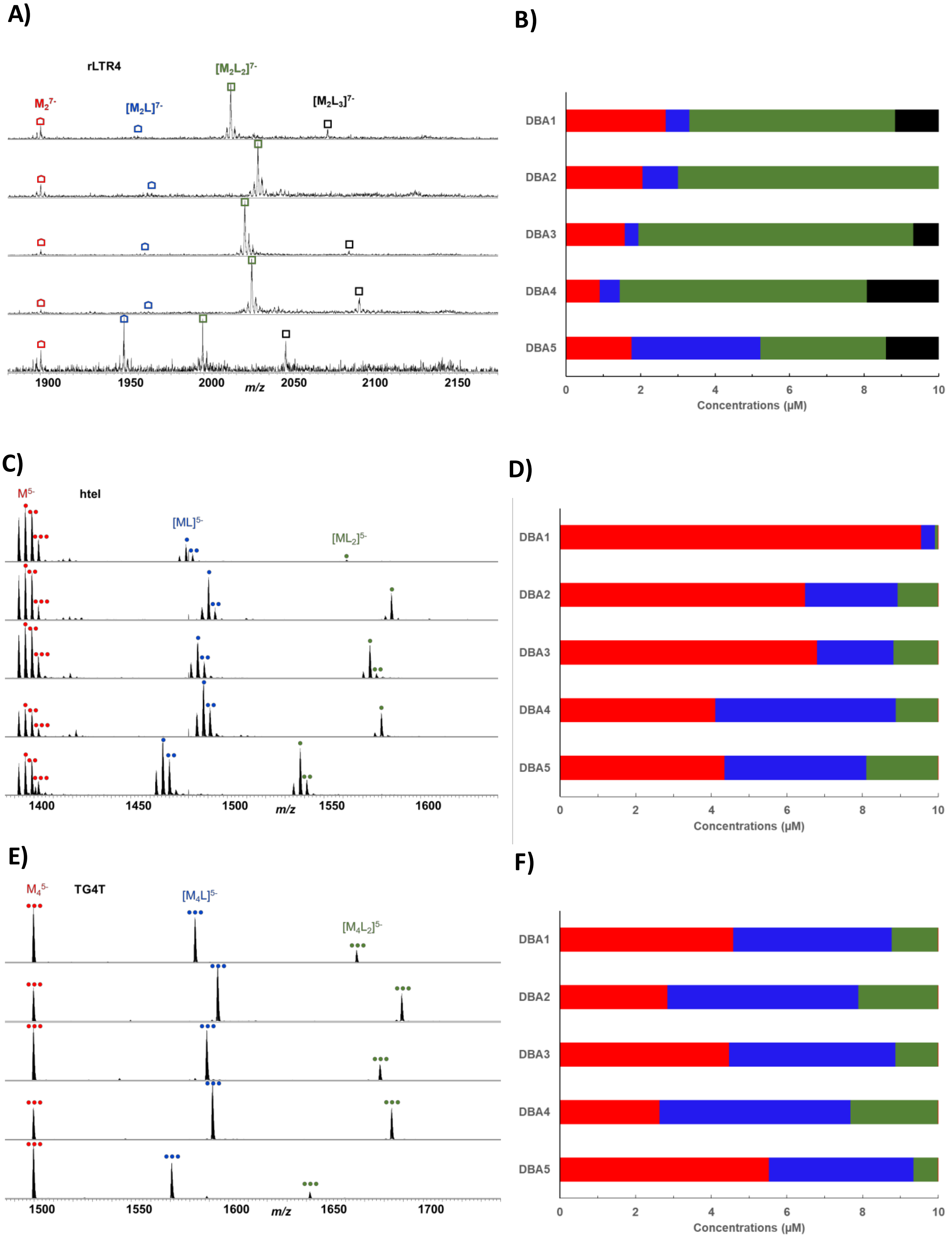

Figure S9: A,C,E) Native electrospray mass spectra of complexes of rLTR4 (A), Htel (C) and TG4T (E) at 10 µM with DBA1-5 (20 µM) in 100 mM ammonium acetate with M = oligonucleotide monomer and L = ligand. For rLTR4, the 7- charge state is highlighted and the most abundant ammonium stoichiometry is labelled with □ (4 NH_4^+) and ⌂ (5 NH_4^+). For Htel and TG4T, The most abundant ammonium stoichiometry is labelled with • (1 NH4+), •• (2 NH4+) and ••• (3 NH4+). **B,D,F)** Concentrations of unbound oligonucleotide (red) or 1:1 (blue), 1:2 (green) and 1:3 (black) complexes of all six oligonucleotides in the presence of DBA1-5 ligands in 100 mM ammonium acetate.

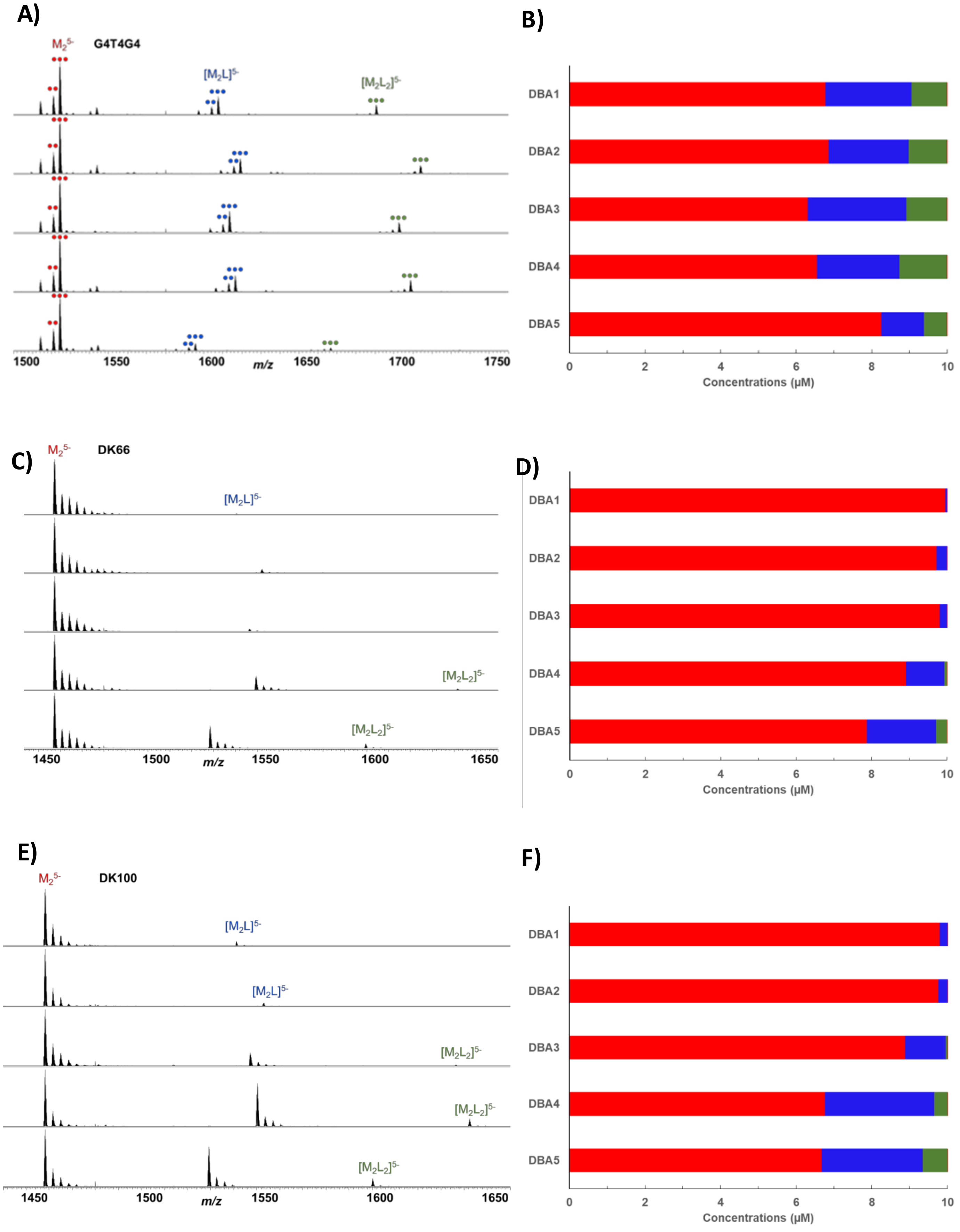

Figure S10: A,C,E) Native electrospray mass spectra of complexes of G4T4G4 (A), DK66 (C) and DK100 (E) at 10 µM with DBA1-5 (20 µM) in 100 mM ammonium acetate with M = oligonucleotide monomer and L = ligand. The most abundant ammonium stoichiometry is labelled with • (1 NH4+), •• (2 NH4+) and ••• (3 NH4+). **B,D,F)** Concentrations of unbound oligonucleotide (red) or 1:1 (blue), 1:2 (green) and 1:3 (black) complexes of all six oligonucleotides in the presence of DBA1-5 ligands in 100 mM ammonium acetate.

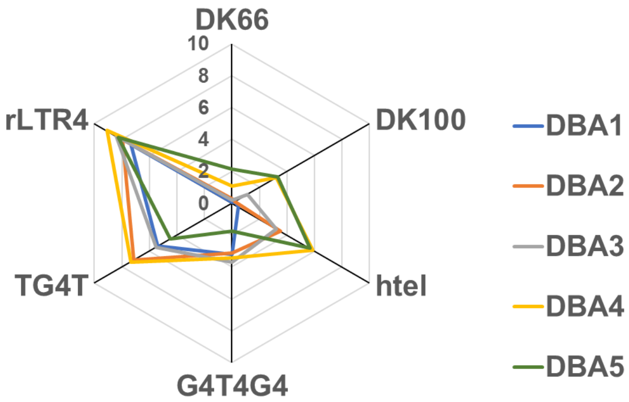

Figure S11: Radar plot representation highlighting the binding specificity profile of each DBA1-5 ligand. Concentration of bound ligand (µM) as determined by native ESI-MS (Figure S9-10) after incubation of DBA1-5 (20 µM) in the presence of various oligonucleotides (10µM) in 100 mM ammonium acetate.

Table S4: Molecular formulae and corresponding SMILES formulae of the dibenzoacridinium compounds

**DBA1**	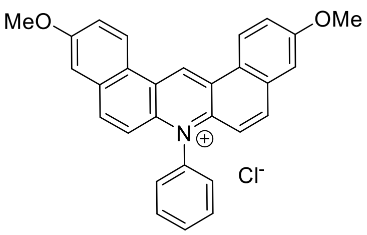	COc6ccc1c(ccc4c1cc3c2ccc(OC)cc2ccc3[n+]4c5ccccc5)c6Cl-
**DBA2**	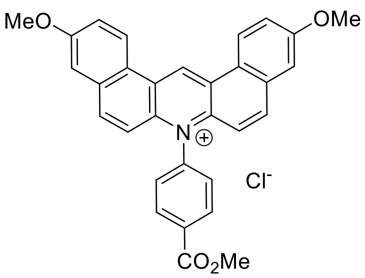	COC(=O)c6ccc([n+]5c2ccc1cc(OC)ccc1c2cc4c3ccc(OC)cc3ccc45)cc6Cl-
**DBA3**	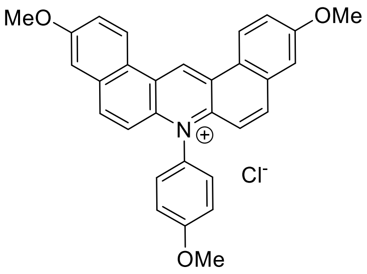	COc6ccc([n+]5c2ccc1cc(OC)ccc1c2cc4c3ccc(OC)cc3ccc45)cc6Cl-
**DBA4**	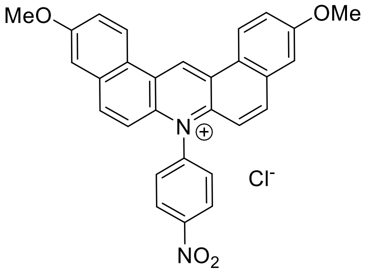	COc6ccc1c(ccc4c1cc3c2ccc(OC)cc2ccc3[n+]4c5ccc([N+](=O)=O)cc5)c6Cl-
**DBA5**	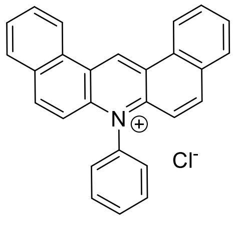	c6ccc([n+]5c2ccc1ccccc1c2cc4c3ccccc3ccc45)cc6Cl-

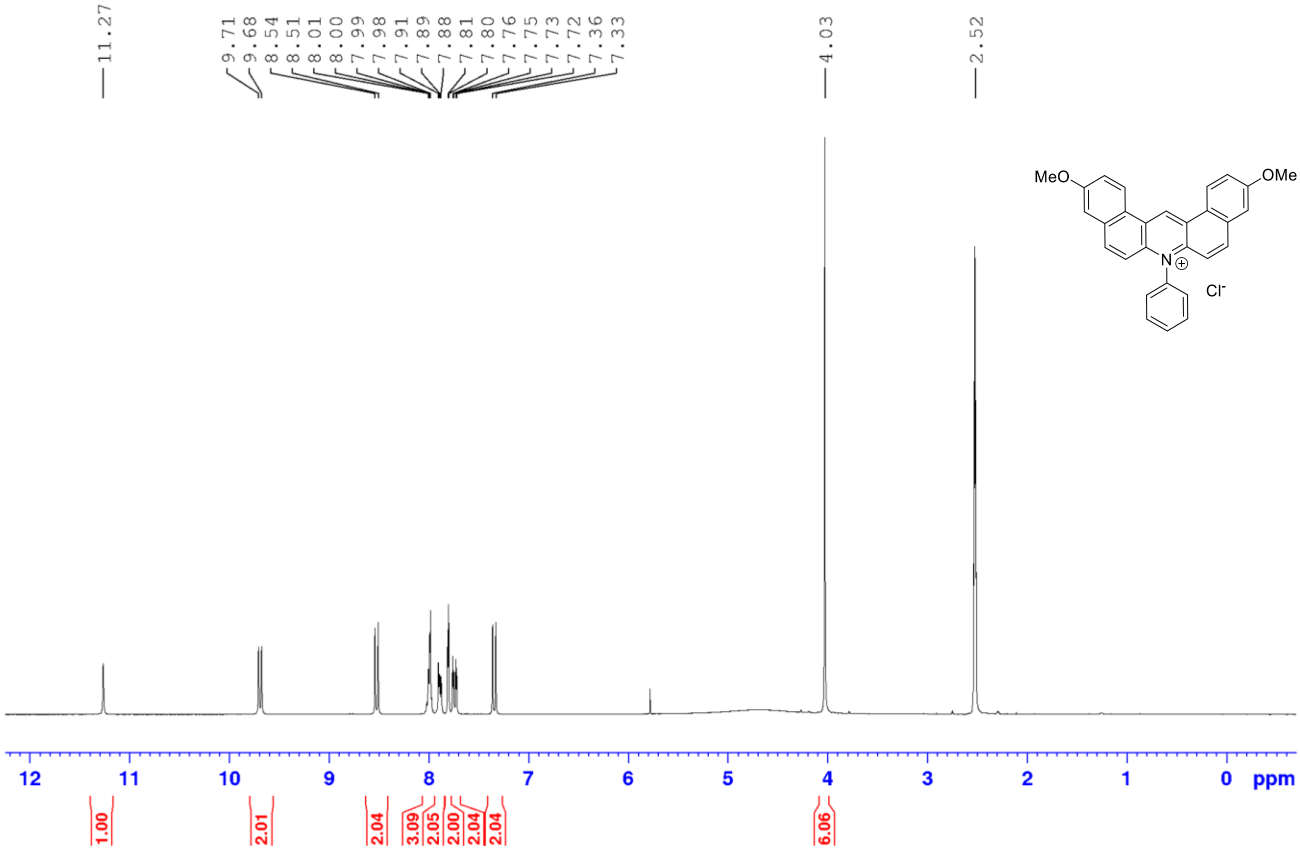

Figure S12: ^{1}H NMR spectrum of **DBA1** (dmso-d6)

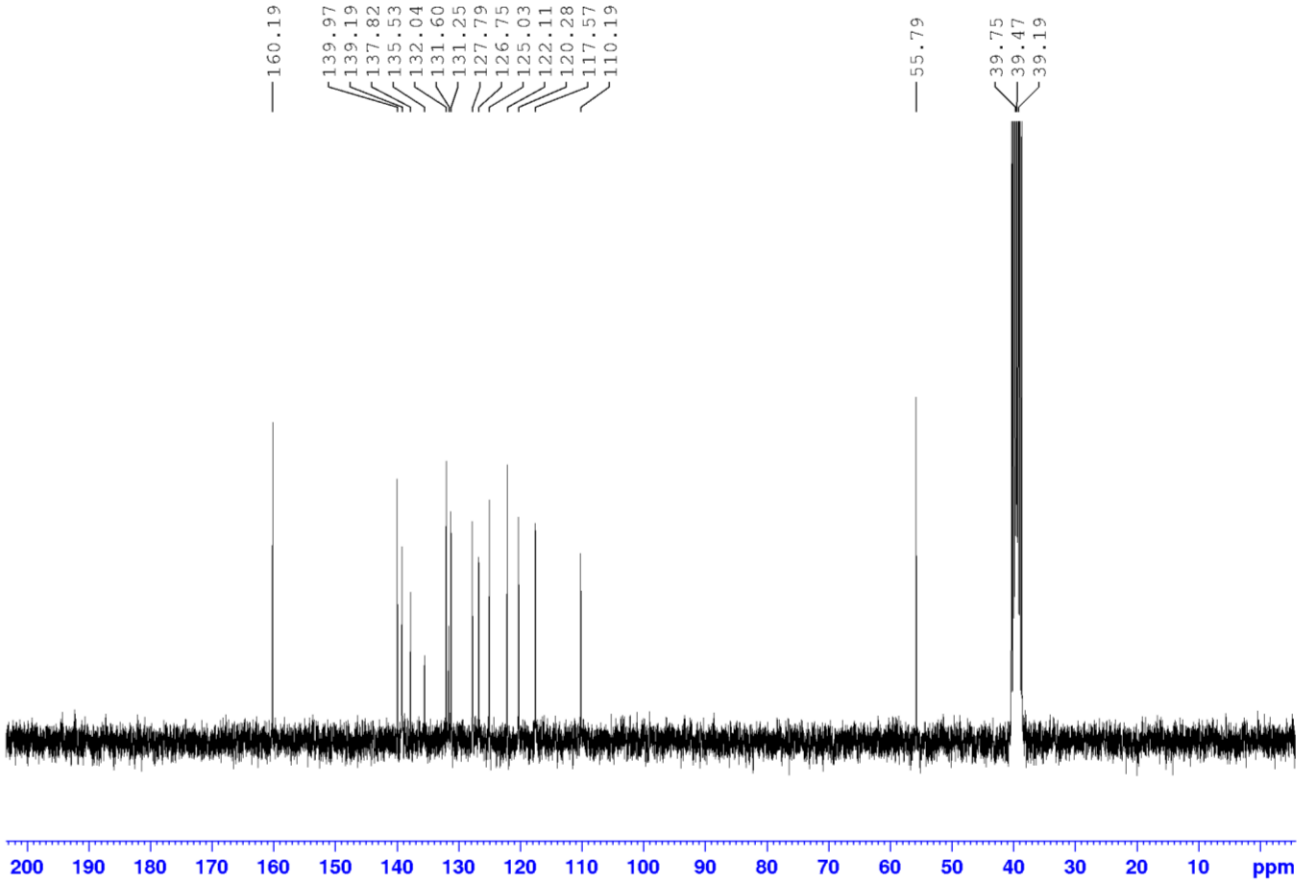

Figure S13: ^{13}C NMR spectrum of **DBA1** (dmso-d6)

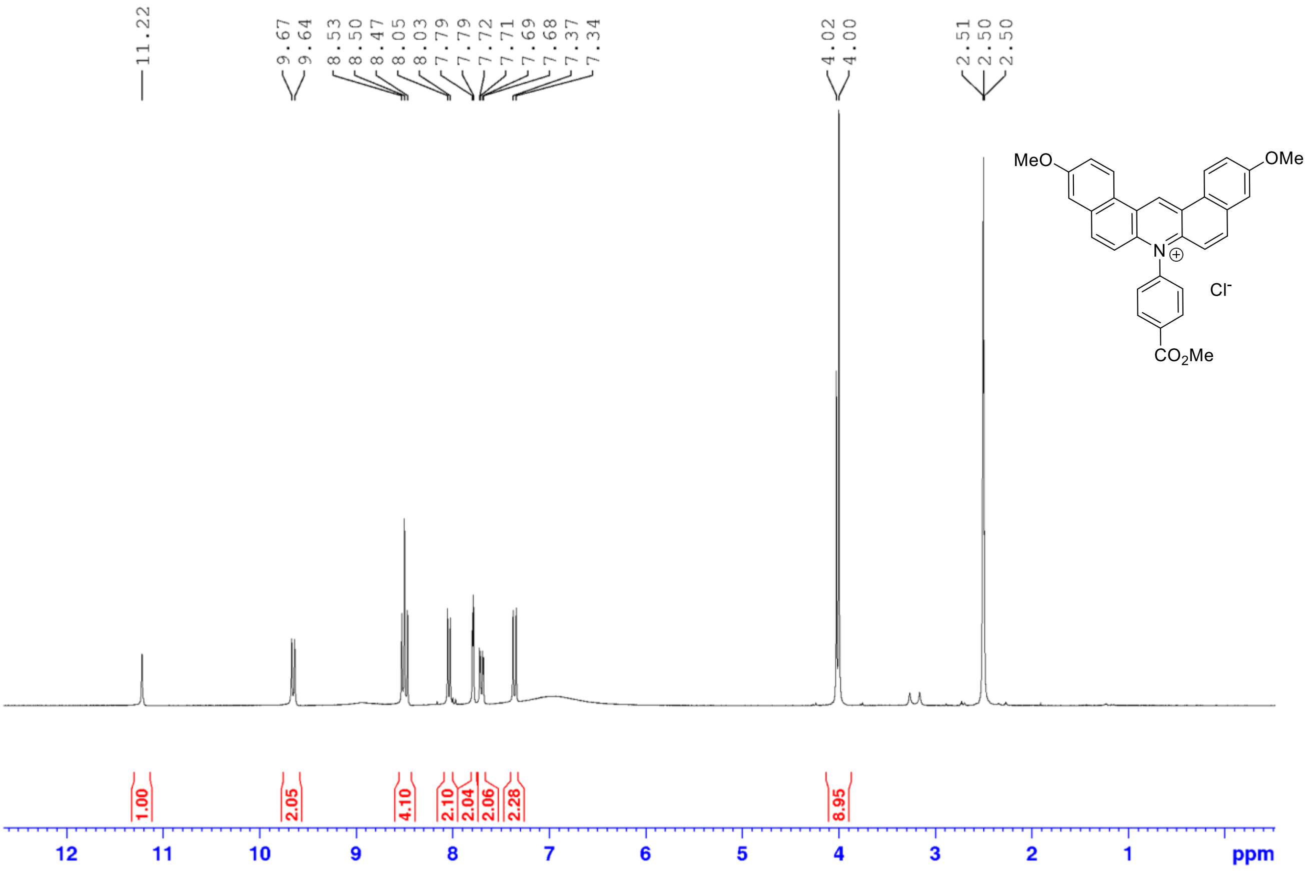

Figure S14: ^{1}H NMR spectrum of **DBA2** (dmso-d6)

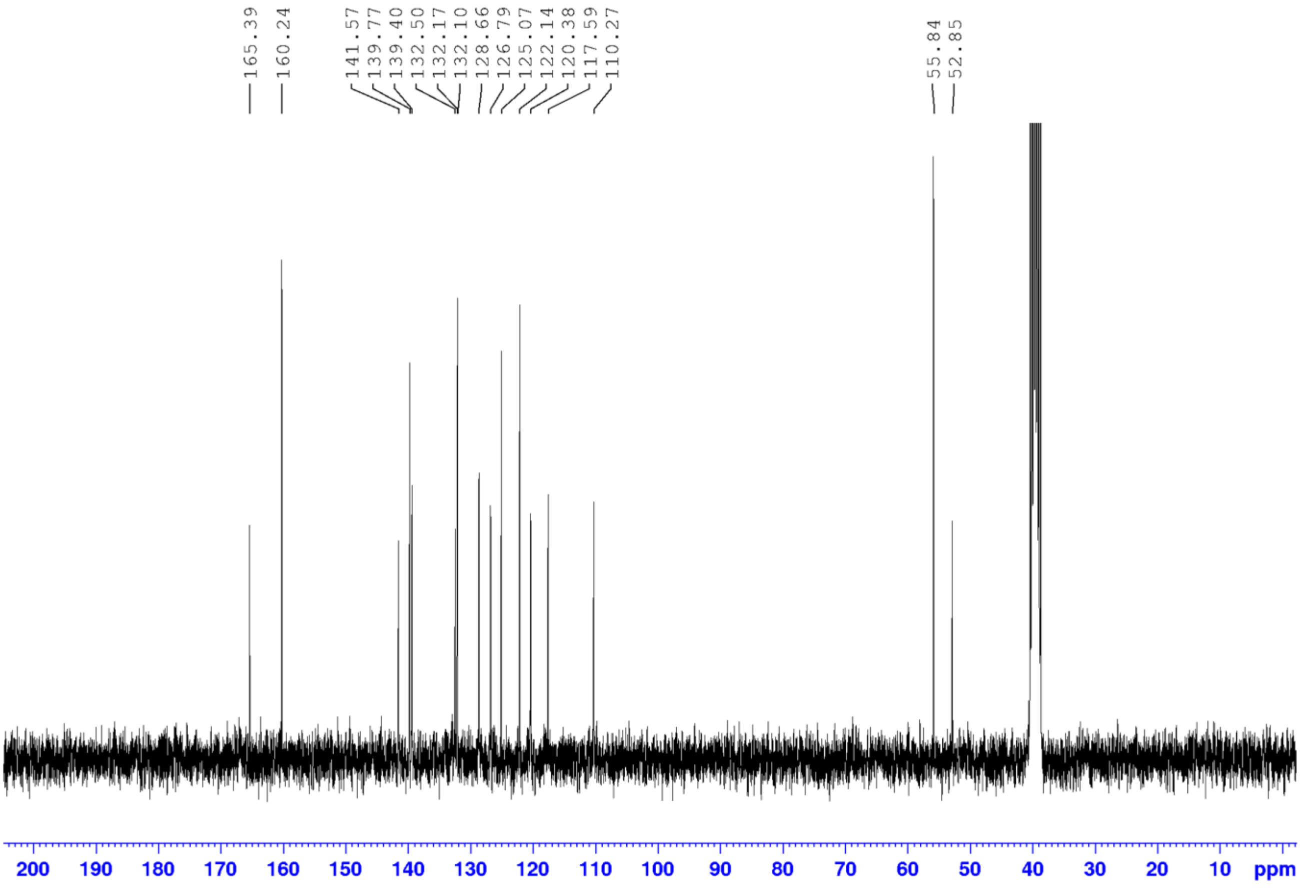

Figure S15: ^{13}C NMR spectrum of **DBA2** (dmso-d6)

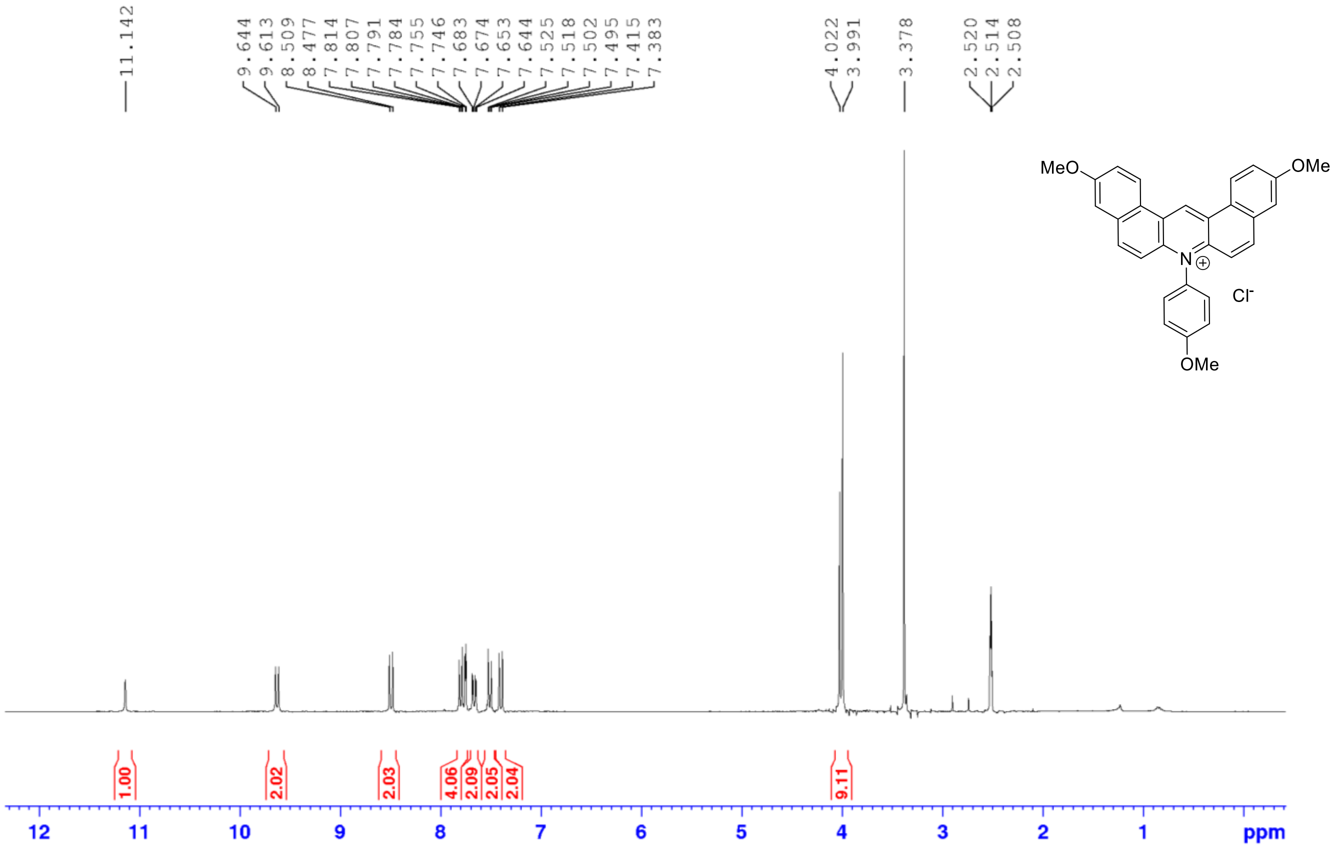

Figure S16: ^{1}H NMR spectrum of **DBA3** (dmso-d6)

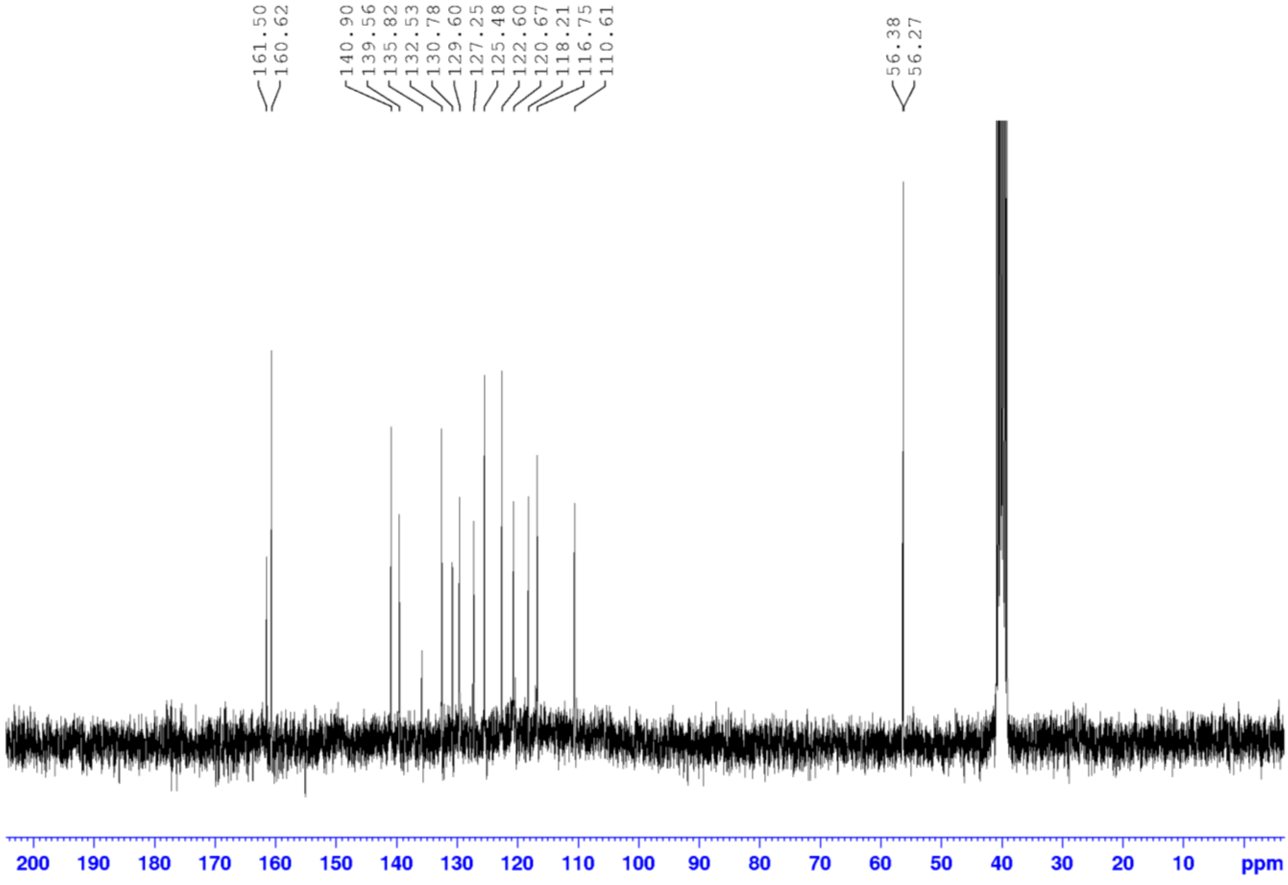

Figure S17: ^{13}C NMR spectrum of **DBA3** (dmso-d6)

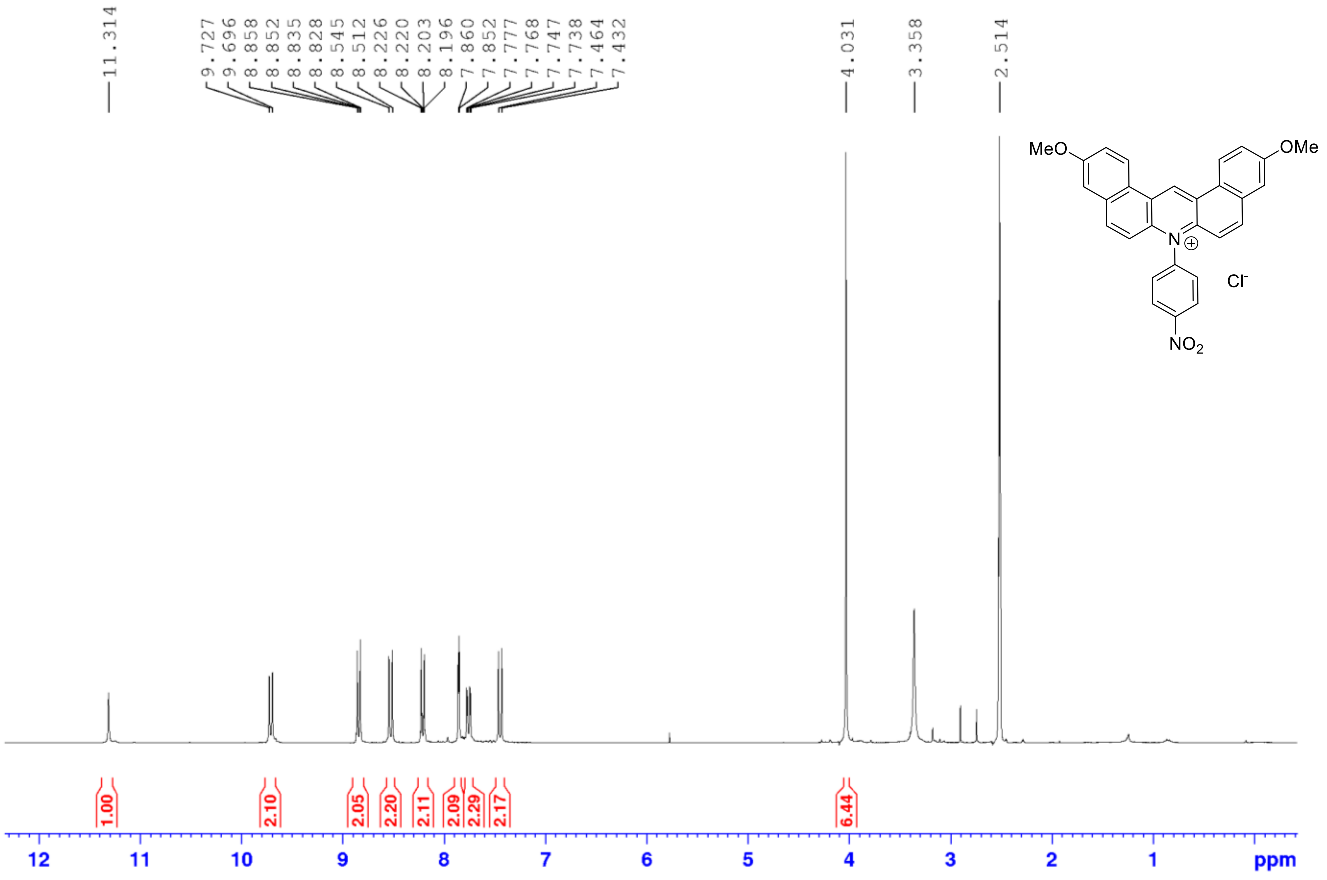

Figure S18: ^{1}H NMR spectrum of **DBA4** (dmso-d6)

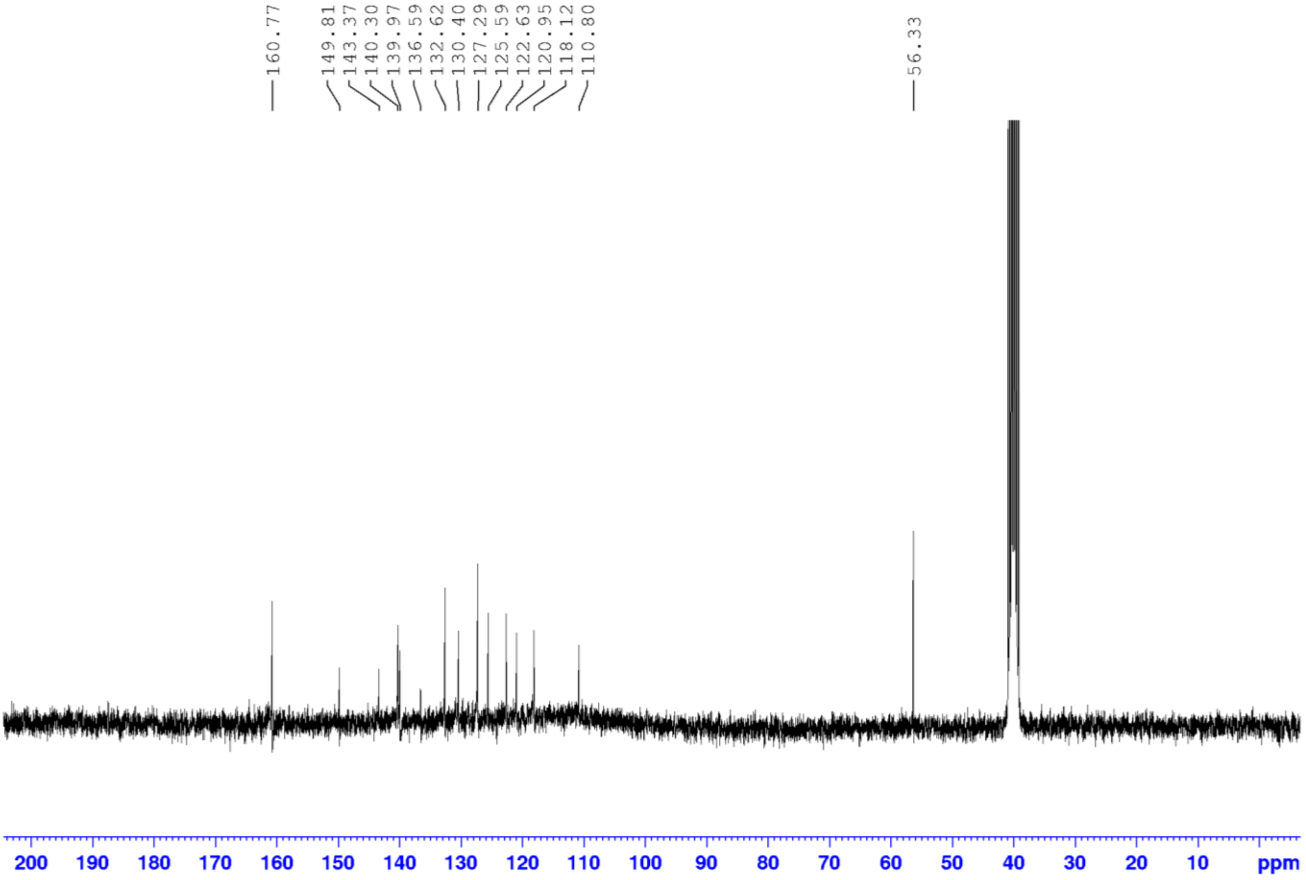

Figure S19: ^{13}C NMR spectrum of **DBA4** (dmso-d6)

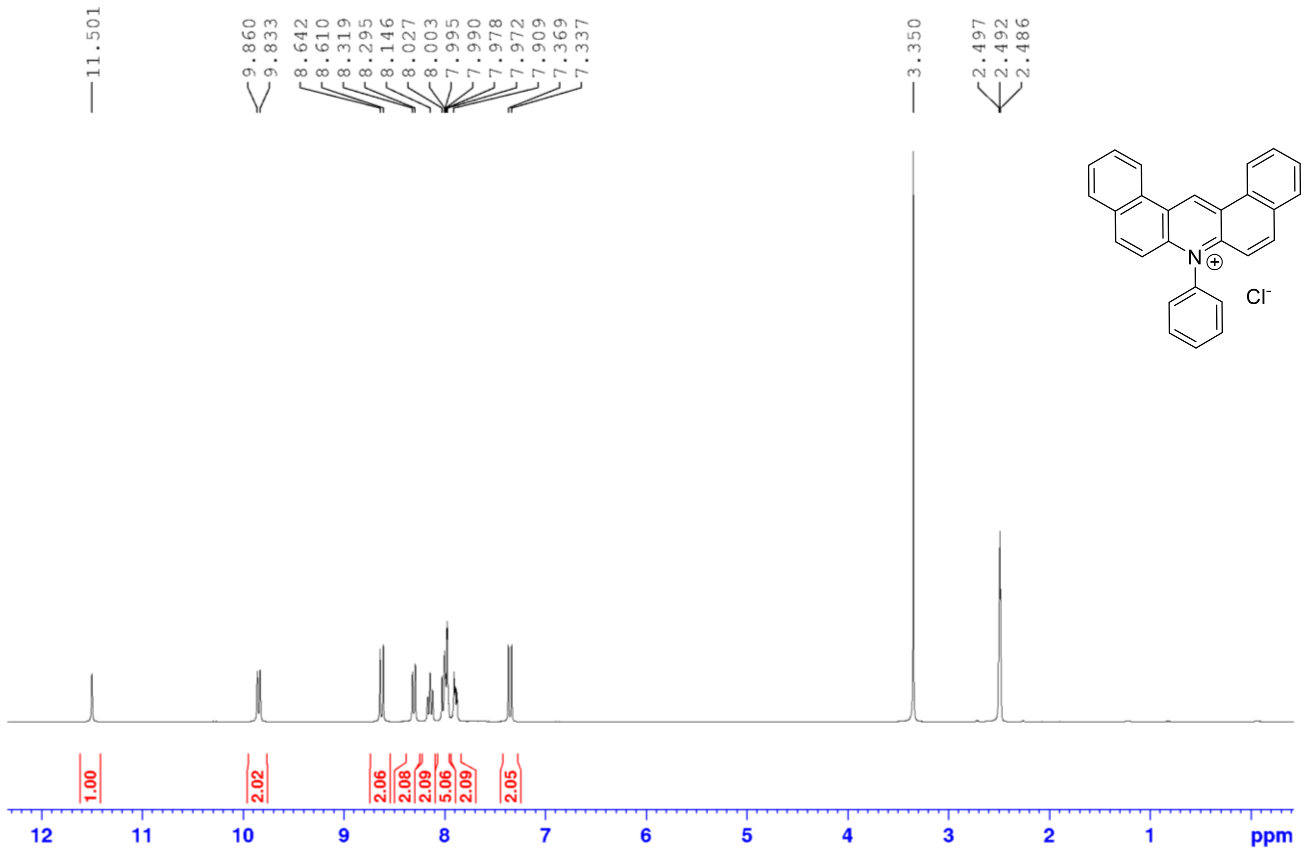

Figure S20: ^{1}H NMR spectrum of **DBA5** (dmso-d6)

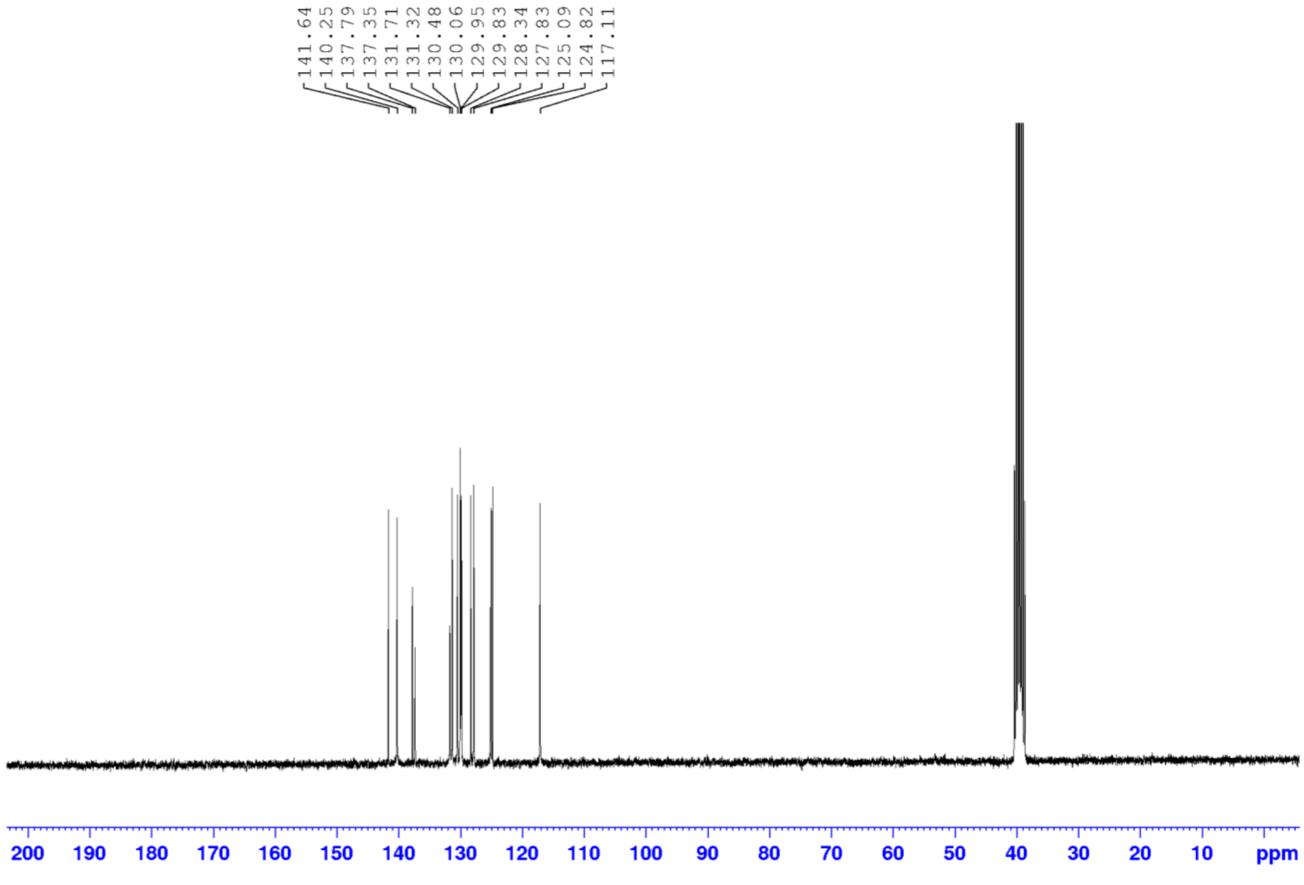

Figure S21: ^{13}C NMR spectrum of **DBA5** (dmso-d6)

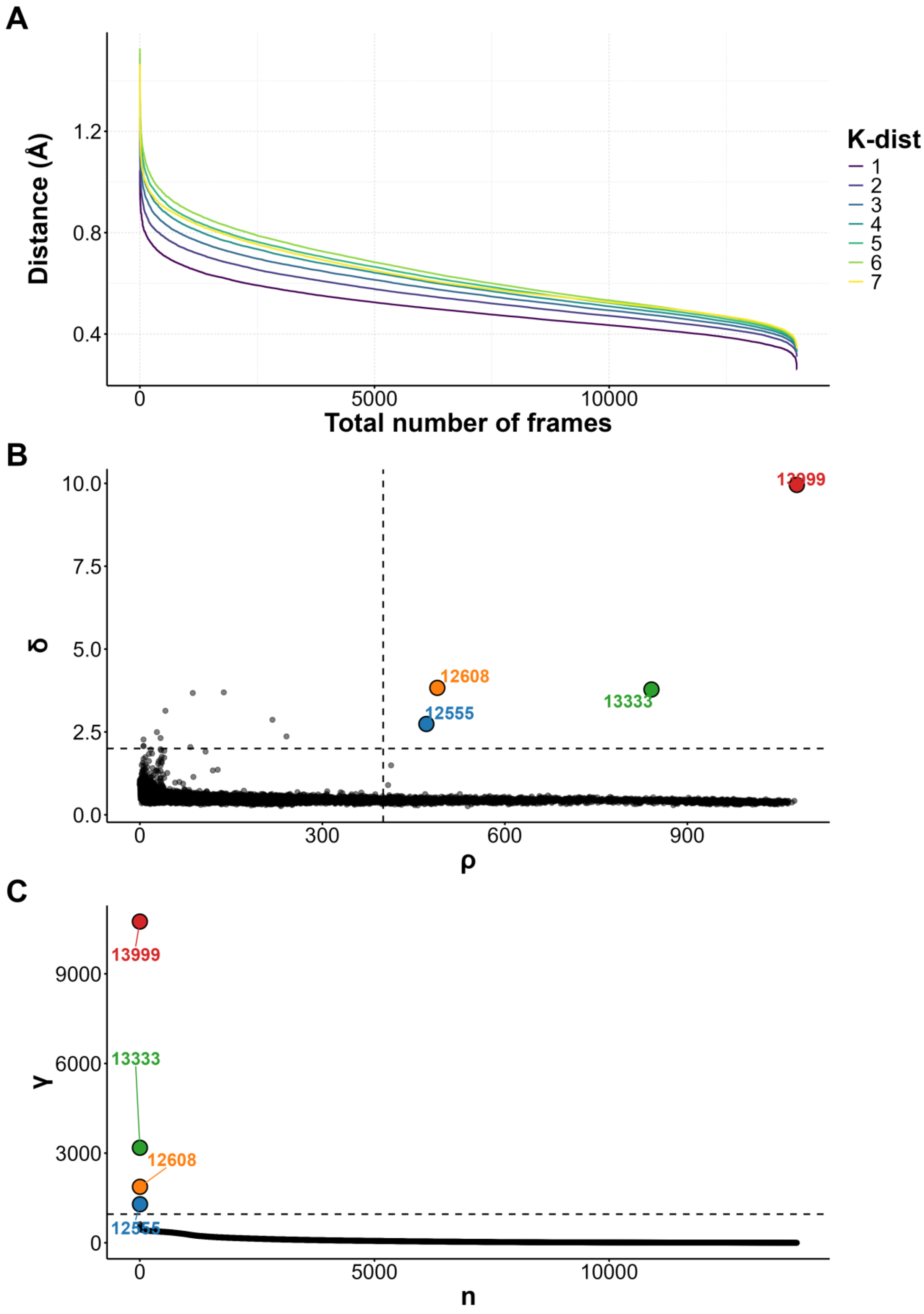

Figure S22: A. K-dist plot showing the Kth farthest distance of each frame, sorted by decreasing distance, obtained with the Dbscan algorithm. B. Decision plot of the DBpeaks algorithm, showing the selected distance (δ) and density (ρ) chosen, as well as the centers colored by cluster. C. $\gamma = \delta\rho$ of each frame sorted in decreasing order, growing very significantly below for clusters ≤ 4, confirming the choice of cut-offs from panel B.
